# Chromosome-level genome assembly of the female western mosquitofish (*Gambusia affinis*)

**DOI:** 10.1093/gigascience/giaa092

**Published:** 2020-08-27

**Authors:** Feng Shao, Arne Ludwig, Yang Mao, Ni Liu, Zuogang Peng

**Affiliations:** Key Laboratory of Freshwater Fish Reproduction and Development (Ministry of Education), Southwest University School of Life Sciences, No. 2 Tiansheng Road, Beibei, Chongqing 400715, China; Department of Evolutionary Genetics, Leibniz-Institute for Zoo and Wildlife Research, Alfred-Kowalke-Straße 17, 10315 Berlin, Germany; Albrecht Daniel Thaer-Institute, Faculty of Life Sciences, Humboldt University Berlin, Invalidenstraße 42, 10115 Berlin, Germany; Key Laboratory of Freshwater Fish Reproduction and Development (Ministry of Education), Southwest University School of Life Sciences, No. 2 Tiansheng Road, Beibei, Chongqing 400715, China; Key Laboratory of Freshwater Fish Reproduction and Development (Ministry of Education), Southwest University School of Life Sciences, No. 2 Tiansheng Road, Beibei, Chongqing 400715, China; Key Laboratory of Freshwater Fish Reproduction and Development (Ministry of Education), Southwest University School of Life Sciences, No. 2 Tiansheng Road, Beibei, Chongqing 400715, China

**Keywords:** Gambusia affinis, Nanopore sequencing, Hi-C, genome assembly, sex chromosome differentiation

## Abstract

**Background:**

The western mosquitofish (*Gambusia affinis*) is a sexually dimorphic poeciliid fish known for its worldwide biological invasion and therefore an important research model for studying invasion biology. This organism may also be used as a suitable model to explore sex chromosome evolution and reproductive development in terms of differentiation of ZW sex chromosomes, ovoviviparity, and specialization of reproductive organs. However, there is a lack of high-quality genomic data for the female *G. affinis*; hence, this study aimed to generate a chromosome-level genome assembly for it.

**Results:**

The chromosome-level genome assembly was constructed using Oxford nanopore sequencing, BioNano, and Hi-C technology. *G. affinis* genomic DNA sequences containing 217 contigs with an N50 length of 12.9 Mb and 125 scaffolds with an N50 length of 26.5 Mb were obtained by Oxford nanopore and BioNano, respectively, and the 113 scaffolds (90.4% of scaffolds containing 97.9% nucleotide bases) were assembled into 24 chromosomes (pseudo-chromosomes) by Hi-C. The Z and W chromosomes of *G. affinis* were identified by comparative genomic analysis of female and male *G. affinis*, and the mechanism of differentiation of the Z and W chromosomes was explored. Combined with transcriptome data from 6 tissues, a total of 23,997 protein-coding genes were predicted and 23,737 (98.9%) genes were functionally annotated.

**Conclusions:**

The high-quality female *G. affinis* reference genome provides a valuable omics resource for future studies of comparative genomics and functional genomics to explore the evolution of Z and W chromosomes and the reproductive developmental biology of *G. affinis*.

## Background

The western mosquitofish (*Gambusia affinis*) is a well-known invasive species of the Poeciliidae family, native to North America. To date, *G. affinis* has invaded many countries worldwide, competing successfully with native fish everywhere and destroying the ecological balance, leading to recognition by the World Conservation Union as one of the world's top 100 invasive alien species.

Although invasive western mosquitofish is a harmful species with regard to the ecological environment, they are useful as model organisms in multiple life science studies, e.g., studies on behavior [1, 2], ecology [3, 4], toxicology [5, 6], and population genetics [7–9]. In addition, western mosquitofish have many interesting biological features. For example, the ZZ/ZW sex determination system in female *G. affinis* contains a W chromosome that is much longer than the Z chromosome [10], which is in contrast to the ZW chromosomes found in many birds and reptiles [11]. Additionally, in terms of reproductive development, female *G. affinis* is an ovoviviparous fish, fertilized in the body and developed in the body; however, without a placenta, the nutrients for the development of the fertilized egg come from yolk and not from maternal supply. Male *G. affinis* horn fins are specialized gonopodium for *in vivo* fertilization. These biological characteristics are generally of interest for biologists because they can provide insights into the evolution of vertebrate sex chromosomes, such as the mechanism of Z and W sex chromosome differentiation. Moreover, on the basis of the reproductive characteristics of female *G. affinis*, this organism may serve as a model for the study of the transition from oviparity to viviparity and provide new perspectives and clues for issues, such as physiological, morphological, and immunological changes to the female reproductive tract.

Male *G. affinis* (ZZ type) scaffold-level genome data have been published [12], and they serve as resources for comparative genomics among poeciliids and teleosts. However, recently released data are not sufficient to explore the evolution of ZW sex chromosomes and the reproduction mode of female *G. affinis*. Further studies are necessary to overcome the lack of high-quality genomic data for female *G. affinis* (ZW type).

Accordingly, in this research, a chromosome (pseudo-chromosomes)-level genome assembly of female *G. affinis* was generated using Oxford Nanopore Technologies (ONT), BioNano, and Hi-C technology. We used genomic data for male *G. affinis* and identified Z and W sex chromosomes. Combined with comparative genomic analysis of the Z and W chromosomes, the high-quality genome produced in this study is expected to provide the foundation for research on the differentiation of sex chromosomes. These data may also help explore the molecular basis of the morphological differences between the *G. affinis* females and males and the characteristics of ovoviviparous reproduction and may aid further studies on functional genomics.

## Methods

### Sample collection

**Figure 1:**
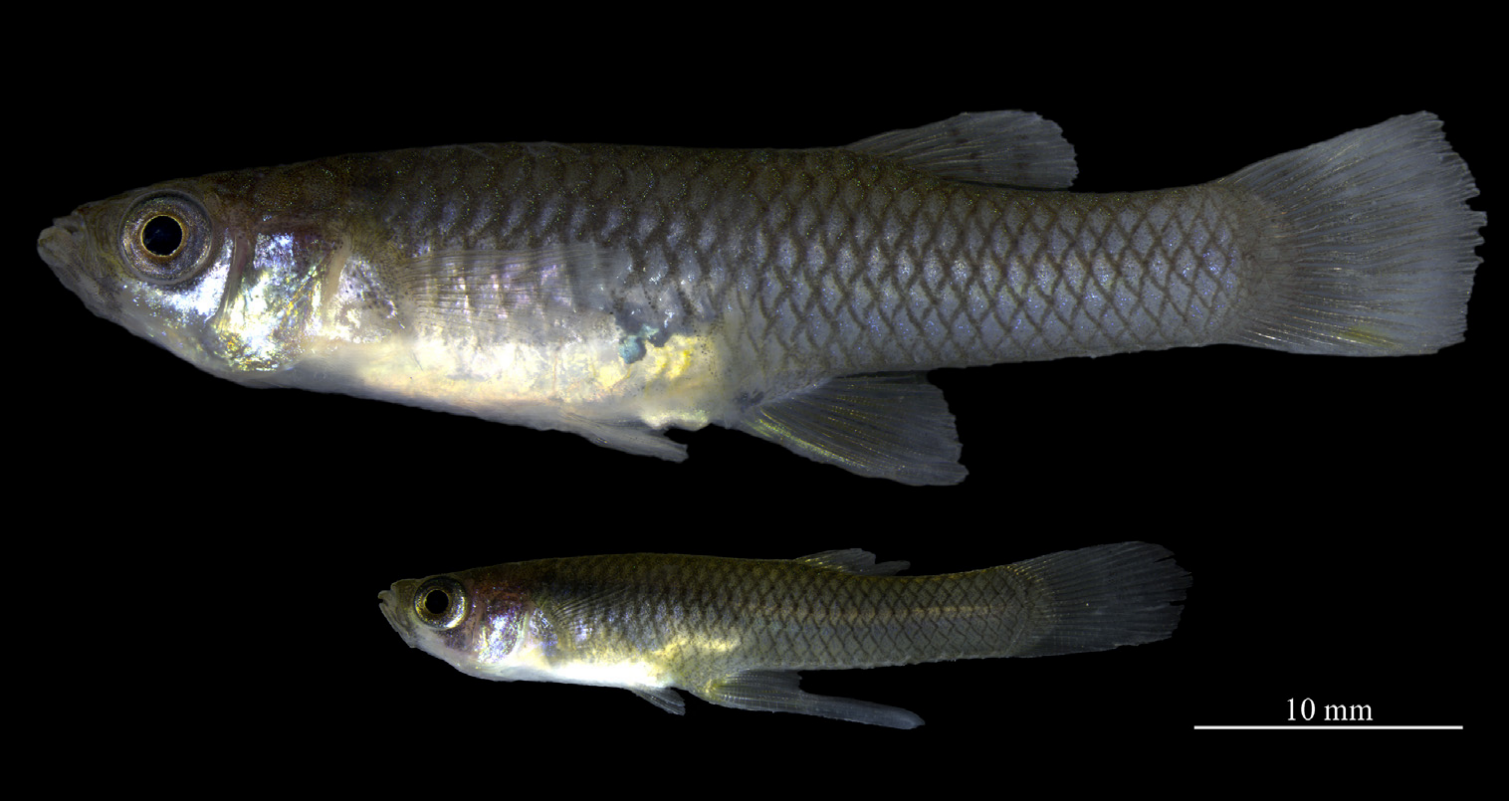
Image of female (top) and male (bottom) *G. affinis*.

Samples for genome sequencing of female *G. affinis* (Fig. 1; sexual dimorphism is pronounced in *G. affinis*: the anal fin of adult females resembles the dorsal fins, while the anal fin of adult males is pointy and specialized for gonopodium) (Fishbase ID: 3215; NCBI:txid33528) were collected from Chongde Lake at the Southwest University in Chongqing, China. The whole body (excluding the gut), brain, liver, heart, gills, gonads, and muscles were collected and quickly frozen in liquid nitrogen. Whole-body samples were used for DNA sequencing, BioNano, and Hi-C for genome assembly, whereas other tissues were used for transcriptome sequencing. Animal research has been approved by the ethics committee of the Southwest University (IACUC No. Approved: IACUC-20190226–19), China.

### DNA library construction and sequencing

Genomic DNA was extracted from the whole body (excluding the gut) using a Qiagen GenomicTip100 (Qiagen, Hilden, Germany). The Illumina TruSeq Nano DNA Library Prep Kit (Illumina, CA, USA) was used to construct an Illumina library with insert sizes of 350 bp, which were then sequenced on an Illumina NovaSeq platform (150-bp paired-end reads). The raw data were filtered using the following strategies: (i) filtered reads with adapters; (ii) removing reads with ≥10% unidentified nucleotides (N); (iii) removing reads with >50% of bases having a phred quality of <5; (iv) removing reads with >10 nt (nucleotide) aligned to the adapter, allowing ≤10% mismatches; and (v) removing putative PCR duplicates generated by PCR amplification in the library construction process. Clean reads were used for subsequent *k-*mer analysis and nanopore data correction.

Approximately 8 μg of genomic DNA was prepared; Blue Pippin (Sage Science, Beverly, MA, USA) and Ligation sequencing 1D kit (SQK-LSK108; ONT, UK) were used for size selection (>10 kb) and nanopore library construction according to the manufacturer's instructions. Two nanopore libraries were constructed and sequenced on 2 different FlowCells using the PromethION sequencer (ONT). Subread quality control was subsequently executed on fast5 files using ONT Albacore software (v0.8.4) [13], and the “passed filter” reads (higher quality reads) were used for subsequent analysis.

### RNA library construction and sequencing

For RNA analyses, 6 tissues (brain, liver, heart, gills, gonads, and muscles) were extracted using an RNeasy Plus Mini Kit (Qiagen) from 5 individuals. The RNA purity, degradation/contamination, concentration, and integrity were measured using NanoDrop One (Thermo Fisher Scientific, MA, USA), 1% agarose gels, Qubit RNA Assay Kit with a Qubit 3.0 Fluorometer (Life Technologies, CA, USA), and the RNA Nano 6000 Assay Kit with a Bioanalyzer 2100 system (Agilent Technologies, CA, USA), respectively. The RNA quality criteria for the RNA samples were as follows: RNA integrity number >8.0 and OD 260/280 between 2.0 and 2.2. Validated RNA samples (from brain, liver, heart, gill, gonad, and muscle tissues) were used for Illumina library construction and sequencing and Pacific Biosciences (PacBio) library preparation (pooled samples), construction, and sequencing.

For Illumina paired-end sequencing (Illumina Novaseq platform, 150-bp), the complementary DNA (cDNA) library was prepared using a TruSeq Sample Preparation Kit (Illumina). The clean data were obtained by removing reads containing adapters, reads containing poly-N, and low-quality reads from the raw data. Qualified RNA from 6 tissues were mixed in equal amounts, reverse-transcribed using a Clontech SMARTer PCR cDNA Synthesis Kit (TaKaRa, Beijing, China), and subjected to PCR amplification using a PrimeSTAR GXL DNA polymerase, and the obtained 0.5–6-kb fragments were retained for PacBio sequencing library construction using a SMRTbell Template Prep Kit (PacBio, CA, USA). Finally, a library for single-molecule real-time sequencing (SMRT) cell was sequenced using polymerase and V2.1 chemistry on a PacBio Sequel platform with 10 h of movie time.

### Genomic features from *k*-mer analysis and nanopore assembly building

Clean reads obtained from the Illumina NovaSeq platform were applied to estimate the genome size and heterozygosity of the western mosquitofish by *k-*mer analysis (17-mer frequency distribution) using jellyfish (jellyfish, RRID:SCR_005491) v2.0 [14].

Filtered ONT sequencing data were corrected by Nextdenovo [15] and using the following parameters: read cutoff = 3k, seed cutoff = 25k, block size = 2 g. Then, ONT sequencing data were assembled using wtdbg (wtdbg, RRID:SCR_017225) v1.2.8 [16]; the pipeline and parameters were as follows: wtdbg-1.2.8 -k 0 -p 23 -S 2, wtdbg-cns -c 3 -k 15, kbm-1.2.8 -k 0 -p 21 -S 2 -O 0, wtdbg-cns -k 13 -c 3.

BWA (BWA, RRID:SCR_010910) v0.7.12 [17] and Pilon (Pilon, RRID:SCR_014731) v1.21 [18] were used to further improve the accuracy of the assembly, based on 3 rounds of mapping the Illumina reads back to the genome. Then, we used BUSCO (BUSCO, RRID:SCR_015008) v3.0.1 [19] to evaluate the completeness of the genome assembly by searching for annotated genes in the assembly.

### Genome scaffolding with BioNano auxiliary assembly

High molecular weight DNA was isolated from the whole body (excluding the gut) and then labeled with Labeling Master Mix and DLE-1; next, the DNA was imaged automatically with a BioNano Saphyr system. BioNano raw BNX files were *de novo* assembled into genome maps with BioNano Solve [20]. The sorted and autodenoised single molecules were subjected to pairwise comparisons by RefAligner [21] to identify molecule overlaps, and consensus maps were constructed. All molecules were then mapped back to the consensus maps, and the maps were recursively refined and extended (2 times).

### Chromosomal-level genome assembly by Hi-C

The Hi-C library was prepared following a previously described procedure [22] with some modifications. Briefly, fresh whole-body samples (excluding the gut) were cut into 2-cm pieces and treated with 1% formaldehyde for 10 min at room temperature to induce cross-linking. The reaction was quenched by adding 2.5 M glycine to a final concentration of 0.2 M for 5 min. Nuclei were digested, marked, and ligated using DpnII, biotin-14-dCTP (Invitrogen, Carlsbad, CA, USA), and T4 DNA Ligase, respectively. After incubation overnight for reverse cross-linking, the ligated DNA was sheared into 300–600-bp fragments. The DNA fragments were blunt-end repaired and A-tailed, followed by purification through biotin-streptavidin–mediated pulldown. Finally, the Hi-C libraries were quantified and sequenced using an Illumina Hiseq platform (150-bp paired-end reads).

In total, 556 million paired-end reads were generated from the Hi-C libraries. Low-quality reads (quality scores <15), adapter sequences, N ratio >5% reads, and reads shorter than 30 bp were filtered out using fastp (fastp, RRID:SCR_016962) v0.12.6 [23], and the clean paired-end reads (549 million paired-end reads; 81,713,239,521 bp) were then mapped to the draft assembled sequence using bowtie2 v2.3.2 [24] to yield unique mapped paired-end reads.

As a result, 141 million uniquely mapped paired-end reads were generated, of which 76.82% were valid interaction pairs. Combined with the valid Hi-C data, we subsequently used the LACHESIS (LACHESIS, RRID:SCR_017644) [25] *de novo* assembly pipeline to produce chromosome-level sequences with the following parameters: (1) CLUSTER MIN. RE SITES = 150; (2) CLUSTER MAX. LINK DENSITY = 2.5; (3) CLUSTER NONINFORMATIVE RATIO = 1.4; (4) ORDER MIN. N RES. IN TRUNK = 60; and (5) ORDER MIN. N RES. IN SHREDS = 60. The interaction heat map of the initial assembly results of LACHESIS was drawn, according to the interaction between different scaffolds, the position and direction of the scaffolds that obviously did not meet the chromosome interaction characteristics in the figure were adjusted. Of note, if there were situations in a scaffold itself that did not meet the chromosome interaction characteristics, the scaffold was interrupted. Next, the scaffolds were adjusted separately until the overall heat map conformed to the characteristics of chromosome interaction.

We used the same method to assemble the genome of a published male *G. affinis* ( GCA_003097735.1) and obtained chromosomal-level genomic data.

### Annotation of repetitive elements

Simple sequence repeat (SSR) sequences in the genome were analyzed by MISA (MISA, RRID:SCR_010765) [26]. For transposable elements (TEs), we first used RepeatModeler (RepeatModeler, RRID:SCR_015027) v2.0.1 [27], LTR_FINDER (LTR_FINDER, RRID:SCR_015247) [28], and MITE-Hunter software [29], based on the principle of *de novo* methods and TE-specific architecture to build a *G. affinis* TE sequence library. The data were then combined with Repbase [30] to construct the final database. Finally, RepeatMasker (RepeatMasker, RRID:SCR_012954) v4.0.5 [31] was used to predict the TEs in male and female *G. affinis* according to the final constructed TE database.

### Gene prediction and function annotation

First, for homology-based prediction, the RNA-seq bam file from mapped reads to the genome by HISAT2 (HISAT2, RRID:SCR_015530) v2.1.0 [32] and protein sequences from 5 sequenced vertebrates, *Danio rerio* (GCA_000002035.4), *Oryzias latipes* (GCA_002234715.1), *Nothobranchius furzeri* (GCA_001465895.2), *Xiphophorus maculatus* (GCA_002775205.2), and *Poecilia formosa* (GCA_000485575.1), were used to predict *G. affinis* genes by GeMoMa (GeMoMa, RRID:SCR_017646) v2.3 [33]. Second, we used Augustus (Augustus, RRID:SCR_008417) v2.5.5 [34] for *ab initio* prediction, a training set generated from the GeMoMa results and transcripts of *G. affinis*, and transcripts obtained from high-throughput data using HISAT2 combined with Stringtie (Stringtie, RRID:SCR_016323) v1.3.5 [35]. Full-length transcriptome data were used to construct consensus sequences through clustering with IsoSeq3 [36]. These sequences were then compared with reference genomes using GMAP (GMAP, RRID:SCR_008992) [37]; next, both transcripts were integrated to remove redundancy, and the results were then processed with PASA (PASA, RRID:SCR_014656) [38] to obtain the final results. Augustus' predictions were compared with the Pfam database [39] to remove genes without domains, and the results of Augustus and GeMoMa were further removed by alternative splicing and integrated. Finally, the TransposonPSI [40] alignment was used to remove sequences containing transposons, yielding the final results.

Functional annotation of the predicted genes of *G. affinis* was performed by alignment to the SwissProt [41], TrEMBL [41], KEGG [42], and Gene Ontology (GO) [43] databases using BLAST (BLAST, RRID:SCR_004870) v2.3.0 and KAAS (v2.1) [44]. Motifs and domains were annotated using InterProScan (InterProScan, RRID:SCR_005829) v5.24 [45].

### Noncoding RNA prediction

Ribosomal RNAs (rRNAs), small nuclear RNAs (snRNAs), microRNAs, and transfer RNAs (tRNAs) were identified by adopting Infernal v1.1.2 [46] using the Rfam database (release 13.0) [47] for the *G. affinis* genome using BLASTN (BLASTN, RRID:SCR_001598) E-value ≤ 1e−5 [48]. tRNAs were predicted using tRNAscan-SE (tRNAscan-SE, RRID:SCR_010835) v1.3.1 [49] with default parameters for eukaryotes. The rRNAs and their subunits were predicted using RNAmmer (RNAmmer, RRID:SCR_017075) v1.2 [50].

### Evolutionary and comparative genomic analyses

We used OrthoMCL (OrthoMCL, RRID:SCR_007839) version 2.0.9 [51] to cluster the female *G. affinis* annotated genes with an E-value cutoff of 1e–5 and Markov chain clustering with default inflation parameters for an all-to-all BLASTP (BLASTP, RRID:SCR_001010) analysis of entries for the reference genomes of 11 fishes, including *G. affinis* in this study and 10 other published fishes reported to date (*Poecilia reticulata, P. formosa, Poecilia latipinna, Poecilia mexicana, Xiphophorus couchianus, N. furzeri, Cyprinodon variegatus, Fundulus heteroclitus, Lepisosteus oculatus*, and *Oreochromis niloticus*). CAFE (CAFE, RRID:SCR_005983) v4.0.1 [52] was used to identify expanded and contracted gene families in *G. affinis*, and these data were then used for GO and KEGG enrichment analyses.

The orthologous genes obtained from the above analyses were subjected to multiple sequence alignment using Mafft (Mafft, RRID:SCR_011811) v7.313 [53] and Gblocks (Gblocks, RRID:SCR_015945) v0.91b [54] to extract conserved sites based on the GTRGAMMA model and RAxML (RAxML, RRID:SCR_006086) v8.2.11 [55]. Using this tree, MCMCTREE in PAML (PAML, RRID:SCR_014932) v4.9e [56] was applied to estimate the 95% confidence intervals of the differentiation times, where the published timings for the divergence of difference species were obtained with the TimeTree database [57].

The orthologous genes were then used in PAML codon substitution models and likelihood ratio tests (codeml) based on the branch-site model to calculate Ka and Ks, yielding positively selected genes, which were then used for GO and KEGG enrichment analyses.

### Recognition and comparison of the W and Z chromosomes of *G. affinis*

Mummer (Mummer, RRID:SCR_018171) v3.0 [58] was used for aligning entire genomic DNA sequences from *X. couchianus*, and male and female *G. affinis*, to make the chromosome numbering system of both species the same. The W chromosome was identified by the specificity of the female *G. affinis* W chromosome length, and then, the Z chromosome in the male *G. affinis* was identified on the basis of synteny. Mummer was also used for aligning entire genomic DNA sequences from the Z and W chromosomes, and Circos plot distributions of homologous sequence pairs among the Z and W chromosome pairs were plotted using Circos (Circos, RRID:SCR_011798) v0.69–6 [59].

According to the results of the RepeatMasker analysis of female and male *G. affinis* genomes, the length and distribution of TEs on chromosomes Z and W were compared. OrthoFinder (OrthoFinder, RRID:SCR_017118) v2.3.8 [60] was used to compare the genes on chromosomes Z and W.

### TE insertion time analyses

We calculated female and male *G. affinis* TE insertion times in genomes using the algorithm *T* = *K*/2*r*, where *K* is the Kimura distance-based copy divergence of TEs and *r* is the nucleic acid substitution rate. The *K*-value was obtained from RepeatMasker. To estimate *r*-values for *G. affinis*, we used LASTZ (LASTZ, RRID:SCR_018556) v1.04.00 [61], chainNet (v2) [62], and MULTIZ (v11.2) [63], along with genomes used in evolutionary analyses and the female *G. affinis* genome as a reference sequence. With the whole-genome alignments, we used the msa_view tool in the PHAST package (PHAST, RRID:SCR_003204) v1.2.1 [64] to extract 4D site alignments, based on the female *G. affinis* gene annotations. The phyloFit program in the PHAST package was used to estimate the phylogenetic model, with tree topology (result of evolutionary analyses) as an input parameter. The branch length results were represented as units of substitutions per site. We calculated the root-to-tip substitution rates from the most recent common ancestor of selected species to each fish lineage, and then divided the root-to-tip substitution rates by the divergence time (314.47 million years ago [Mya]) of most recent common ancestor of selected species.

## Results and Discussion

### Female *G. affinis* genome initial characteristics

A total of 30.5 Gb Illumina clean reads were used for analyzing female *G. affinis* genome size and heterozygosity using *k-*mer analysis. Based on 26,474,864,304 17-mers and a peak 17-mer depth of 37 (Supplementary Fig. S1), the estimated heterozygosity rate was ~0.42%, and the estimated genome size of female *G. affinis* was ~715 Mb. Of note, the estimated genome size is similar to that of the nuclear DNA content estimated in a previous study using flow cytometry (0.75 pg, ~733 Mb) [65].

### *De novo* assembly of a female *G. affinis* reference genome

Next, 74.4 Gb (2,866,145 reads, average read length of 25.98 kb, N50 35.86 kb, and longest read length of 273 kb) ONT clean long reads were used to construct the reference genome. We obtained a 662-Mb genomic DNA sequence by assembly with a contig N50 length of 12.9 Mb.

**Figure 2:**
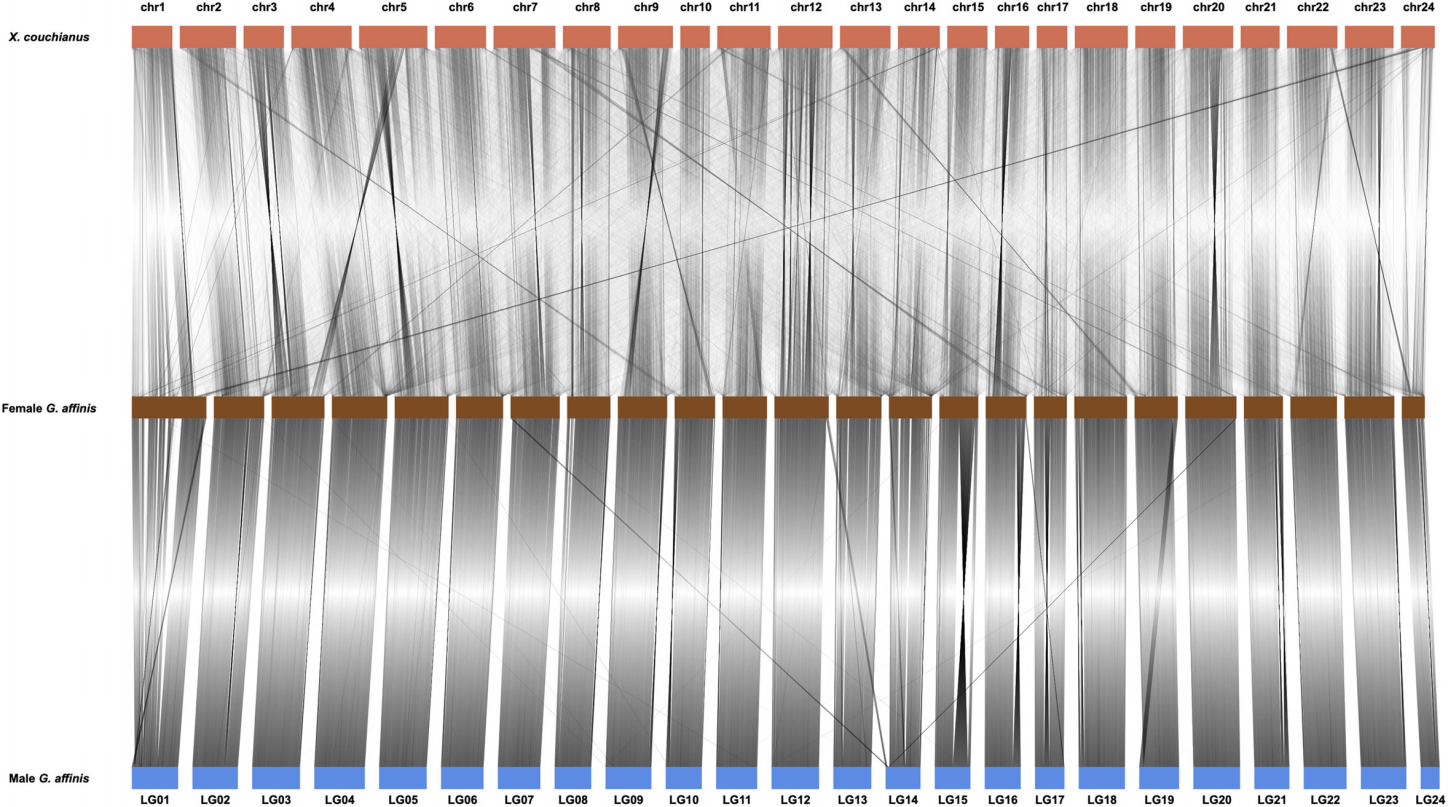
Genomic synteny of *X. couchianus*, female *G. affinis*, and male *G. affinis*. Female *G. affinis* LG01 represents the W chromosome and male *G. affinis* LG01 represents the Z chromosome.

The long-reads assembly result consisted of 217 contigs, and the longest contig was 28.6 Mb. Then, BUSCO was used to assess the completeness of the assembled genome. Approximately 97.2% of the complete genes could be detected in the genome of female *G. affinis*, confirming the completeness of the genome. Assembly results of long-reads scaffolds obtained using optical maps were assembled with 80-Gb BioNano molecules. The final assembly contained 125 scaffolds with a scaffold N50 size of 26.4 Mb. Finally, we used the Hi-C technique to anchor the assembly scaffolds in 24 chromosomes of female *G. affinis* (Supplementary Table S1). We found that 141,528,181 unique mapped paired-end reads were generated and occupied ~51.5% of the total clean paired-end reads (274,658,176). Then, the frequency of scaffold interactions was estimated on the basis of the pairs mapped to the scaffolds. We found that 113 scaffolds were successfully anchored in 24 chromosomes (Fig. 2, Supplementary Fig. S2a), consistent with the records of the chromosome number by cytogenetic analysis [10, 66], representing 90.4% of all scaffolds and 97.9% of all scaffold nucleotide bases. The total assembly size of the chromosomes was ~679.4 Mb (Table 1). In the male *G. affinis* genomic DNA sequences, 734 scaffolds were successfully anchored in 24 chromosomes (Fig. 2, Supplementary Fig. S2b), and the total assembly size was 592.7 Mb.

**Table 1:** Genome assembly statistics of *Gambusia affinis*

Statistic	ONT	BioNano	Hi-C	
			Female^#^	Male^##^
Total assembly size of contig/scaffold/chromosome (bp)	662,579,534	680,140,492	679,423,294*	592,666,412*
No. of contig/scaffold/chromosome	217	125	24	24
N50 contig/scaffold/chromosome length (bp)	12,906,370	26,455,434	29,761,488	25,946,590
N90 contig/scaffold/chromosome length (bp)	1,629,223	18,394,109	23,709,503	21,272,223
Longest contig/scaffold/chromosome (bp)	28,665,999	31,542,956	45,125,082	30,583,032

* The length of 24 chromosomes, excluding the length of the unanchored sequences.

# Based on the data generated in this study.

## Based on published data [12].

### Genome annotation

Assembled chromosome-level genome of female *G. affinis* was used to predict repeat sequences. In total, 5,630,271 SSRs were identified, including 5,478,552 mono-, 100,272 di-, 28,431 tri-, 19,721 tetra-, 2,048 penta-, and 1,247 hexa-nucleotide repeats. Overall, the combined homology-based and *de novo* prediction results indicated that TEs accounted for 22.54% of the assembly genome (Supplementary Table S2). Additionally, class I TEs (RNA transposons) occupied ~5.15% of the assembly genome. The most abundant RNA transposons found in the *G. affinis* assembly genome were long interspersed nuclear elements, which constituted 54.37% of all identified RNA transposons. The female *G. affinis* genome was very rich in class II TEs (DNA transposons), which occupied 11.83% of genome content.

For genome annotation, 23,997 protein-coding genes were predicted in the female *G. affinis* genome. Compared with other existing published poeciliid fish annotated information, the number of genes in female *G. affinis* was similar to those in *P. formosa* (23,615 genes) and *X. maculatus* (23,628 genes) (Supplementary Table S3 and Fig. S3). BUSCO gene prediction was carried out using the actinopterygii_odb9 single-copy homologous gene to predict the existing sequence of the genome. Approximately 97% of complete gene components could be found in this gene set, indicating that most of the conserved genes were well predicted and that the prediction results were relatively reliable (Supplementary Table S4). Finally, 23,737 genes were annotated in ≥1 of the databases (KOG, KEGG, NR, SwissProt, GO), and up to 98.92% of *G. affinis* genes were functionally annotated (Supplementary Table S5). Finally, 143 snRNAs, 220 rRNAs, 371 microRNAs, and 3,885 tRNAs were also identified.

### Genome evolution

**Figure 3:**
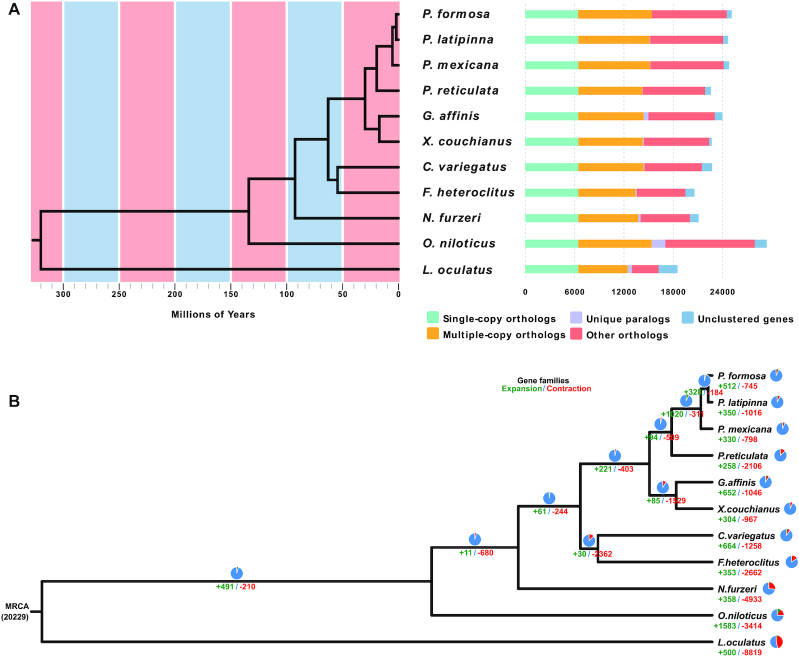
Phylogenetic and evolutionary analysis of *G. affinis*. (A) Divergence time estimates and gene clusters in *G. affinis* and other species. (B) Expansion and contraction of *G. affinis* gene families. MRCA: most recent common ancestor; pie charts and numbers below represent the proportion and specific values of the gene families of expansion (green) and contraction (red), respectively.

To determine the evolutionary relationships between *G. affinis* and other vertebrates, a phylogenetic tree was reconstructed on the basis of 6,457 single-copy orthologous genes from 10 other vertebrate genomes (Fig. 3a). *L. oculatus* and *O. niloticus* were used as outgroups. As a species of the family Poeciliidae, *G. affinis* clustered into 1 branch with other fish from Poeciliidae. Compared with 6 other sequenced members of the Poeciliidae family, *G. affinis* had a closer relationship with *X. couchianus*, consistent with previously published phylogenies [67]. Next, a timetree was created on the basis of the above 6,457 single-copy orthologous genes, and the estimated divergence time between *G. affinis* and *X. couchianus* was found to be ~16.57 Mya (Fig. 3; Supplementary Fig. S3). In addition, the divergence time between *G. affinis* and 4 other members of the Poeciliidae family was ~22.75 Mya.

To examine the evolutionary history of gene families, we performed gene family expansion and contraction analysis with the female *G. affinis* genes. We found 652 expansion gene families and 1,046 contraction gene families (Fig. 3b). Expansion gene families were enriched in 44 GO (Supplementary Table S6) categories and 34 KEGG pathways (Supplementary Table S7), most of which were related to oxygen metabolism, olfactory pathways, and visual pathways. Next, codeml was used to calculate the average Ka/Ks values and conduct branch-site likelihood ratio analyses to detect positively selected genes in the female *G. affinis* genome. The results showed that there were 590 positively selected genes in the female *G. affinis* genome. The positively selected genes were enriched in 12 GO categories (aspartic-type endopeptidase activity, DNA repair, microtubule binding, insulin-like growth factor binding, tRNA aminoacylation for protein translation, microtubule motor activity, rRNA processing, microtubule-based movement, protein dephosphorylation, protein tyrosine phosphatase activity, chromatin binding, and nucleus) and 3 KEGG pathways (complement and coagulation cascades, peroxisome, and platelet activation).

### Recognition and evolution of sex chromosomes

Genetic-controlled sex determination systems in fish are variable, ranging from XX/XY to ZZ/ZW [68]. Fish generally do not have highly morphologically differentiated sex chromosomes, making it difficult to distinguish between autosomes and sex chromosomes. Hence, there are only a few fish species for which there is known information on sex determination mechanisms and sex chromosome systems. Therefore, a suitable experimental model is required for the identification and elucidation of the mechanisms of fish sex chromosome evolution, and the female *G. affinis* is a suitable and consistent model.

**Figure 4:**
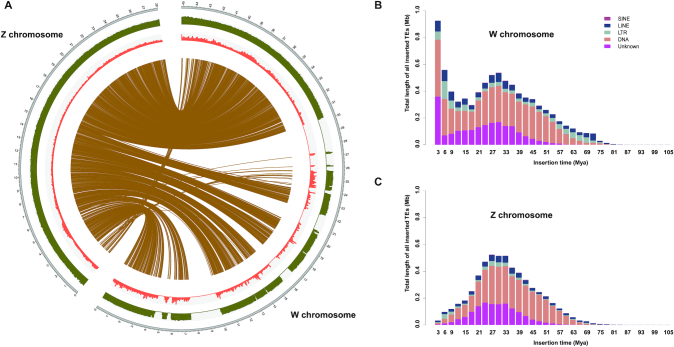
Comparative genomic analysis of the Z and W chromosomes. (A) Circos plot of Z and W chromosome alignment; the red region represents the repeat sequence density, and the green region represents the GC density. (B) Distribution of the transposon activity time for the W chromosome. (C) Distribution of transposon activity time for the Z chromosome. LINE: long interspersed nuclear element; LTR: long terminal repeat; SINE: short interspersed nuclear element.

Early karyotype analysis demonstrated that female *G. affinis* shows heterogamy of the ZW type, and its W chromosome is much longer than other chromosomes [10, 66]. The longest sequence was selected from the assembly results at the chromosome level as the W candidate chromosome, and 1 female-specific DNA marker [69] was used for confirmation. In the end, the marker has been aligned to the W candidate chromosome but was not found in the genomic DNA sequences of the male *G. affinis*. Analysis of the synteny of the whole genomes of female and male mosquitofish by Mummer demonstrated that the Z chromosome was also present in the male *G. affinis* genomic DNA sequences (Fig. 2). By comparing the Z and W chromosomes (Fig. 4a), we found the length of the W and Z chromosome repeat sequences to be ~8.5 and 5.0 Mb, respectively. Among them, the length and content of the *Helitron* superfamily of the 2 chromosomes (W: 591,639 bp, 1.3%; Z: 66,868 bp, 0.24%) were significantly different (results have been submitted to GigaDB). There were 1,279 and 1,027 genes on the W and Z chromosomes, respectively. Homology analysis showed that there were 794 one-to-one pairs. There were 118 and 85 genes on the W and Z chromosomes unassigned to any gene groups, and the others were of the one-to-many and many-to-many types; these results provide research directions for our future analyses on functional genomics (results have been submitted to GigaDB).

Some researchers have studied the role of transposons in sex chromosome differentiation, and they found that TEs seem to play an important role in the evolution of sex chromosomes, with their accumulation and loss having huge effects on the lengths of sex chromosomes [70–72]. However, differences in TE contents between Z and W chromosomes alone cannot determine the true course of differentiation, e.g., whether the increased length of the W chromosome compared with that of the Z chromosome is caused by extension of the W chromosome or by degeneration of the Z chromosome. There is no substantial evidence to explain this observation. Therefore, the introduction of the time factor is extremely important. *Gambusia holbrooki* and *G. affinis* are so closely related that for a long time, biologists thought they were the same species. Phylogenetic analyses estimated that their divergence time was ~2–7 Mya [69, 73, 74], and other researchers showed that the XY and ZW sex determination mechanisms had independent origins in *G. holbrooki* and *G. affinis*, respectively [68]. Therefore, we speculate that the differentiation of Z and W sex chromosomes is a very recent event. Additionally, previous studies suggested that this process may be enriched on the W chromosome by TEs, leading to an increase in the sex chromosome size during the early phase of differentiation and the subsequent reduction in size later during evolution [75]. If this hypothesis is correct, then we should be able to observe a large number of transposons inserted in the W chromosome in the recent past (between 2 and 7 Mya). Indeed, our results indicated very recent mass insertion events of TEs into the W chromosome (Fig. 4b), and the insertion time characteristics of the TEs into the W chromosome were specific because its insertion time trends were dramatically different from those of autosomal and Z-chromosomal TEs (Fig. 4c and Supplementary Fig. S5). Moreover, we speculate that most of the long gaps (Fig. 4a) on the W chromosome were also caused by the aggregation of too many highly similar TE sequences to form TE clusters through the recent activation of TEs. Thus, we expected that the TE content of the W chromosome of *G. affinis* should be much higher than that observed to date. Accordingly, our results showed that the cause of sex chromosome differentiation in female *G. affinis* was likely to be related to extension of the W chromosome.

## Conclusions

In this study, we assembled the chromosome-level female western mosquitofish genome using the most mainstream technology available. In terms of parameters such as contig N50, scaffold N50, and gene annotation number, these are high-quality genomic data. Evolutionary analysis provides ideas for future work; e.g., oxygen transport in mosquitofish deserves attention. We conducted a preliminary study on W and Z sex chromosome differentiation based on the specificity of the sex chromosome in female western mosquitofish and provided data to support the previous hypothesis that a longer W chromosome is associated with the activity (insertion) of TEs. In conclusion, our high-quality genomic data lay the foundation for the study of chromosome evolution, reproductive characteristics, and sexual dimorphism in western mosquitofish.

## Availability of Supporting Data and Materials

The raw genome and RNA sequencing data were deposited in the SRA under Bioproject No. PRJNA599452. The chromosome-level genome, annotation, and other supporting data are also available via the *GigaScience* database, GigaDB [76].

## Additional Files

**Table S1:** Result of female and male *Gambusia affinis* genomic assembly at chromosome level.

**Table S2:** Transposable elements (TEs) annotation in the female *Gambusia affinis* genome.

**Table S3:** Comparative analysis of the annotated gene set of female *Gambusia affinis* with those of 5 teleosts.

**Table S4:** Assessment of female *Gambusia affinis* genome completeness by BUSCO.

**Table S5:** Statistics for gene function annotation in female *Gambusia affinis* genome.

**Table S6:** Expansion gene families of female *Gambusia affinis* were enriched in 44 GO categories.

**Table S7:** Expansion gene families of female *Gambusia affinis* were enriched in 34 KEGG pathways.

**Figure S1:** Frequency distribution of the 17-mer graph analysis used to estimate the size of female *Gambusia affinis*.

**Figure S2:** Western mosquitofish genome scaffold contact matrix using Hi-C data. (a) Female western mosquitofish. (b) Male western mosquitofish. The color bar indicates the contact density from red (high) to white (low).

**Figure S3:** The comparisons of coding sequence length, exon length, exon number, gene length, intron length, and intron number in the genomes of female *Gambusia affinis* and other teleosts.

**Figure S4:** Divergence time of *Gambusia affinis* and other fish species.

**Figure S5:** Distribution of transposon activity time for different autosomes of female *Gambusia affinis*.

**Additional File:** All genes located on the Z and W sex chromosomes with their locations.

giaa092_GIGA-D-20-00179_Original_Submission

giaa092_GIGA-D-20-00179_Revision_1

giaa092_GIGA-D-20-00179_Revision_2

giaa092_Response_to_Reviewer_Comments_Original_Submission

giaa092_Response_to_Reviewer_Comments_Revision_1

giaa092_Reviewer_1_Report_Original_Submission Shigehiro Kuraku -- 7/11/2020 Reviewed

giaa092_Reviewer_2_Report_Original_Submission LÃ¡szlÃ3 Orban, Ph.D. -- 7/13/2020 Reviewed

giaa092_Supplemental_Files

## Abbreviations

BLAST: Basic Local Alignment Search Tool; bp: base pairs; BUSCO: Benchmarking Universal Single-Copy Orthologs; BWA: Burrows-Wheeler Aligner; CAFE: Computational Analysis of gene Family Evolution; cDNA: complementary DNA; GeMoMa: Gene Model Mapper; Gb: gigabase pairs; GO: Gene Ontology; Hi-C: High-throughput chromosome conformation capture; IACUC: Institutional Animal Care and Use Committee; KAAS: KEGG Automatic Annotation Server; kb: kilobase pairs; KEGG: Kyoto Encyclopedia of Genes and Genomes; KOG: EuKaryotic Orthologous Groups; LACHESIS: Ligating Adjacent Chromatin Enables Scaffolding *In Situ*; Mb: megabase pairs; MRCA: most recent common ancestor; Mya: million years ago; LTR: long terminal repeat; NCBI: National Center for Biotechnology Information; ONT: Oxford Nanopore Technologies; PacBio: Pacific Biosciences; PAML: Phylogenetic Analysis by Maximum Likelihood; PASA: Program to Assemble Spliced Alignments; RAxML: Randomized Accelerated Maximum Likelihood; RNA-seq: RNA sequencing; rRNA: ribosomal RNA; SMRT: single-molecule real-time sequencing; snRNA: small nuclear RNA; SRA: Sequence Read Archive; SSR: simple sequence repeat; TE: transposable element; tRNA: transfer RNA.

## Competing Interests

The authors declare that they have no competing interests.

## Authors' Contributions

F.S. performed the major part of data analysis and drafted the manuscript. Y.M. and N.L. contributed to sample collection and drafted the manuscript. Z.P. and A.L. contributed to research design and final edits to the manuscript. All authors read and approved the final manuscript.
