# Supplementary material for: Chromosome-level genome assembly of the female western mosquitofish (Gambusia affinis)
Source: Gigascience. 2020 Aug 27;9(8):giaa092. doi: 10.1093/gigascience/giaa092 (PMC7450667; doi:10.1093/gigascience/giaa092)
Supplement: giaa092_GIGA-D-20-00179_Original_Submission [file giaa092_giga-d-20-00179_original_submission.pdf]

GigaScience

Chromosome-level genome assembly of the female western mosquitofish (Gambusia affinis)

--Manuscript Draft--

**Manuscript Number:**	GIGA-D-20-00179
**Full Title:**	Chromosome-level genome assembly of the female western mosquitofish (Gambusia affinis)
**Article Type:**	Data Note
**Funding Information:**	National Key Research and Development Program of China (2018YFD0900805) — Prof. Zuogang Peng<br>National Natural Science Foundation of China (31872204) — Prof. Zuogang Peng<br>Fundamental Research Funds for the Central Universities (XDJK2020C043) — Dr. Feng Shao<br>Natural Science Foundation of Chongqing Postdoctoral Science Foundation (cstc2019jcyj-bshX0071) — Dr. Feng Shao
**Abstract:**	Background<br>The western mosquitofish (Gambusia affinis) is a sexual dimorphism poeciliid fish known for its worldwide biological invasion and is an important research model for studying invasion biology. This organism may also be used as a suitable model to explore sex chromosome evolution and reproductive development in terms of differentiation of ZW sex chromosomes, ovoviviparity, and specialization of reproductive organs. However, there is a lack of high-quality genomic data for female G. affinis; hence, this study aimed to generate a chromosome-level genome assembly of the female G. affinis.<br>Results<br>The chromosome-level genome assembly was constructed using Oxford nanopore sequencing, BioNano, and Hi-C technology. A G. affinis genome containing 217 contigs with an N50 length of 12.9 Mb and 125 scaffolds with an N50 length of 26.5 Mb was obtained by Oxford nanopore and BioNano, respectively, and the 113 scaffolds (90.4% of scaffolds containing 97.9% nucleotide bases) were assembled into 24 chromosomes by Hi-C. The Z and W chromosomes of G. affinis were identified by comparative genomic analysis of female and male G. affinis, and the mechanism of differentiation of the Z and W chromosomes was explored. Combined with transcriptome data from six tissues, a total of 23,997 protein-coding genes were predicted and 23,737 (98.9%) genes were functionally annotated.<br>Conclusions<br>The high-quality female G. affinis genome obtained in this study provides a valuable omics resource for future studies of comparative genomics and functional genomics to explore the evolution of Z and W chromosomes and the reproductive developmental biology of G. affinis.
**Corresponding Author:**	Zuogang Peng, Ph.D.<br>Southwest University<br>Chongqing, CHINA
**Corresponding Author Secondary Information:**	
**Corresponding Author's Institution:**	Southwest University
**Corresponding Author's Secondary Institution:**	
**First Author:**	Feng Shao
**First Author Secondary Information:**	

Powered by Editorial Manager® and ProduXion Manager® from Aries Systems Corporation

**Order of Authors:**	Feng Shao
	Arne Ludwig
	Yang Mao
	Ni Liu
	Zuogang Peng
**Order of Authors Secondary Information:**	
**Additional Information:**	
**Question**	**Response**
Are you submitting this manuscript to a special series or article collection?	No
**Experimental design and statistics**<br><br>Full details of the experimental design and statistical methods used should be given in the Methods section, as detailed in our Minimum Standards Reporting Checklist. Information essential to interpreting the data presented should be made available in the figure legends.<br><br>Have you included all the information requested in your manuscript?	Yes
**Resources**<br><br>A description of all resources used, including antibodies, cell lines, animals and software tools, with enough information to allow them to be uniquely identified, should be included in the Methods section. Authors are strongly encouraged to cite Research Resource Identifiers (RRIDs) for antibodies, model organisms and tools, where possible.<br><br>Have you included the information requested as detailed in our Minimum Standards Reporting Checklist?	Yes
**Availability of data and materials**<br><br>All datasets and code on which the conclusions of the paper rely must be	Yes

either included in your submission or deposited in publicly available repositories (where available and ethically appropriate), referencing such data using a unique identifier in the references and in the "Availability of Data and Materials" section of your manuscript.<br><br>Have you have met the above requirement as detailed in our Minimum Standards Reporting Checklist?	

Manuscript Click here to download Manuscript Manuscript.docx

Chromosome-level genome assembly of the female western mosquitofish (*Gambusia affinis*)

Feng Shao[1], Arne Ludwig[2,3], Yang Mao[1], Ni Liu[1], Zuogang Peng[1, *]

[1] Key Laboratory of Freshwater Fish Reproduction and Development (Ministry of Education), Southwest University School of Life Sciences, Chongqing 400715, China

[2] Department of Evolutionary Genetics, Leibniz-Institute for Zoo and Wildlife Research, 10315 Berlin, Germany

[3] Albrecht Daniel Thaer-Institute, Faculty of Life Sciences, Humboldt University Berlin, 10115 Berlin, Germany

Running title: Genome assembly in *G. affinis*

*Correspondence: pzg@swu.edu.cn

Abstract

Background

The western mosquitofish (*Gambusia affinis*) is a sexual dimorphism poeciliid fish known for its worldwide biological invasion and is an important research model for studying invasion biology. This organism may also be used as a suitable model to explore sex chromosome evolution and reproductive development in terms of differentiation of ZW sex chromosomes, ovoviviparity, and specialization of reproductive organs. However, there is a lack of high-quality genomic data for female *G. affinis*; hence, this study aimed to generate a chromosome-level genome assembly of the female *G. affinis.*

Results

The chromosome-level genome assembly was constructed using Oxford nanopore sequencing, BioNano, and Hi-C technology. A *G. affinis* genome containing 217 contigs with an N50 length of 12.9 Mb and 125 scaffolds with an N50 length of 26.5 Mb was obtained by Oxford nanopore and BioNano, respectively, and the 113 scaffolds (90.4% of scaffolds containing 97.9% nucleotide bases) were assembled into 24 chromosomes by Hi-C. The Z and W chromosomes of *G. affinis* were identified by comparative genomic analysis of female and male *G. affinis*, and the mechanism of differentiation of the Z and W chromosomes was explored. Combined with transcriptome data from six tissues, a total of 23,997 protein-coding genes were predicted and 23,737 (98.9%) genes were functionally annotated.

Conclusions

The high-quality female *G. affinis* genome obtained in this study provides a valuable omics resource for future studies of comparative genomics and functional genomics to explore the evolution of Z and W chromosomes and the reproductive developmental biology of *G. affinis*.

Keywords: *Gambusia affinis*, Nanopore sequencing, Hi-C, genome assembly, sex chromosome differentiation

Background

The western mosquitofish (*Gambusia affinis*) is a well-known invasive species of the Poeciliidae family, native to North America. To date, *G. affinis* has invaded many countries worldwide, competing successfully with native fish everywhere and destroying the ecological balance, leading to recognition by the World Conservation Union as one of the world's top 100 invasive alien species (http://www.iucngisd.org/gisd/100_worst.php).

Although invasive western mosquitofish are harmful species with regard to the ecological environment, they are useful as model organisms in multiple life science studies, e.g., studies on behavior [1, 2], ecology [3, 4], toxicology [5, 6], and population genetics [7-9]. In addition, western mosquitofish have many interesting biological features. For example, the ZZ/ZW sex determination system in female *G. affinis* contains a W chromosome that is much longer than the Z chromosome [10], which is in contrast to the ZW chromosomes found in many birds and reptiles [11]. Additionally, in terms of reproductive development, female *G. affinis* is an oviparous fish (without a placenta), fertilized in the body, and developed in the body; however, without a placenta, the nutrients for the development of the fertilized egg come from the yolk, and not from the maternal supply. Male *G. affinis* horn fins are specialized gonopodium for *in vivo* fertilization. These biological characteristics are generally of interest to biologists, as they can provide insights into the evolution of vertebrate sex chromosomes, such as the mechanism of Z and W sex chromosome differentiation. Moreover, based on the reproductive characteristics of female *G. affinis*, this organism may serve as a model for the study of the transition from oviparity to viviparity and provide new perspectives and clues for such studies, such as physiological, morphological, and immunological changes to the female reproductive tract.

Male *G. affinis* (ZZ type) scaffold level genome data have been published [12], and they serve as resources for comparative genomics among poecilids and teleosts. However, recently

released data are not sufficient to explore the evolution of ZW sex chromosomes and the reproduction mode of female *G. affinis*. Further studies are needed to overcome the lack of high-quality genomic data for female *G. affinis* (ZW type).

Accordingly, in this research, a chromosome-level genome assembly of female *G. affinis* was generated using Oxford nanopore, BioNano, and Hi-C technology. We used genomic data for male *G. affinis* and identified Z and W sex chromosomes. Combined with comparative genomic analysis of the Z and W chromosomes, the high-quality genome produced in this study is expected to provide the foundation for research on the differentiation of sex chromosomes, morphological differences between the female and male *G. affinis*, and molecular basis for the characteristics of ovoviviparous reproduction, and meanwhile, aid our subsequent studies on functional genomics.

Methods

Sample collection

Samples for genome sequencing of female *G. affinis* (NCBI taxonomy ID: 33528) were collected from the Chongde Lake at the Southwest University in Chongqing, China. The whole body (excluding the gut), brain, liver, heart, gills, gonads, and muscles were collected and quickly frozen in liquid nitrogen. Whole body samples were used for DNA sequencing, BioNano, and Hi-C for genome assembly, whereas other tissues were used for transcriptome sequencing. All animal experimental procedures were approved by the ethics committee of the Southwest University (IACUC No. Approved: IACUC-20190226-19).

DNA library construction and sequencing

Genomic DNA was extracted from the whole body (excluding the gut) using a Qiagen GenomicTip100 (Qiagen, Hilden, Germany). Illumina TruSeq Nano DNA Library Prep Kit

(Illumina, USA) was used to construct an Illumina library with insert sizes of 350 bp, which were then sequenced on an Illumina NovaSeq platform (150-bp paired-end reads). The raw data were filtered using the following strategies: (i) filtered reads with adapters; (ii) removing reads with greater than or equal to 10% unidentified nucleotides (N); (iii) removing reads with more than 50% of bases having a phred quality of less than 5; (iv) removing reads with more than 10 nt aligned to the adapter, allowing less than or equal to 10% mismatches; and (v) removing putative polymerase chain reaction (PCR) duplicates generated by PCR amplification in the library construction process. Clean reads were used for subsequent *K-mer* analysis and nanopore data correction.

Approximately 8 μg of gDNA was prepared; Blue Pippin (Sage Science, Beverly, MA, USA) and Ligation sequencing 1D kit (SQK-LSK108; ONT, UK) were used for size selection (more than 10 kb) and nanopore library construction according to the manufacturer's instructions. Two nanopore libraries were constructed and sequenced on two different FlowCells using the PromethION sequencer (ONT). Subreads quality control was subsequently executed on fast5 files using ONT Albacore software (v0.8.4), and the "passed filter" reads (higher quality reads) were used for subsequent analysis.

RNA library construction and sequencing

For RNA analyses, six tissues (brain, liver, heart, gills, gonads, and muscles) were extracted using an RNeasy Plus Mini Kit (Qiagen) from five individuals. The RNA purity, degradation/contamination, concentration, and integrity were measured using NanoDrop One (Thermo Fisher Scientific, USA), 1% agarose gels, Qubit RNA Assay Kit with a Qubit 3.0 Fluorometer (Life Technologies, CA, USA), and the RNA Nano 6000 Assay Kit with a Bioanalyzer 2100 system (Agilent Technologies, CA, USA), respectively. The RNA quality criteria for the RNA samples were as follows: RNA integrity number more than 8.0 and OD

260/280 between 2.0 and 2.2. Validated RNA samples (from brain, liver, heart, gill, gonad, and muscle tissues) were used for Illumina library construction and sequencing and PacBio library preparation (pooled samples), construction, and sequencing.

For Illumina paired-end sequencing (Illumina Novaseq platform, 150-bp), the cDNA library was prepared using a TruSeq Sample Preparation Kit (Illumina). The clean data were obtained by removing reads containing adapters, reads containing poly-N, and low-quality reads from the raw data. Qualified RNA from six tissues were mixed in equal amounts, reverse-transcribed using a Clontech SMARTer PCR cDNA Synthesis Kit (TaKaRa), and subjected to PCR amplification using a PrimeSTAR GXL DNA polymerase, and the obtained 0.5–6-kb fragments were retained for PacBio sequencing library construction using a SMRTbell Template Prep Kit (Pacific Biosciences). Finally, a library for SMRT cell was sequenced using polymerase and V2.1 chemistry on a PacBio Sequel platform with 10 h of movie time.

Genomic features from K-mer analysis and nanopore assembly building

Clean reads obtained from the Illumina NovaSeq platform were applied to estimate the genome size and heterozygosity of the western mosquitofish by *K-mer* analysis (*17-mer* frequency distribution) using jellyfish [13].

Filtered Oxford nanopore sequencing data were corrected by Nextdenovo (https://github.com/Nextomics/NextDenovo) and using the following parameters: read cutoff = 3k, seed cutoff = 25k, block size = 2 g. Then, Oxford nanopore sequencing data were assembled using wtdbg [14]; the pipeline and parameters were as follows: wtdbg-1.2.8 -k 0 -p 23 -S 2, wtdbg-cns -c 3 -k 15, kbm-1.2.8 -k 0 -p 21 -S 2 -O 0, wtdbg-cns -k 13 -c 3.

BWA v0.7.12 [15] and Pilon [16] were used to further improve the accuracy of the assembly, based on three rounds of mapping the Illumina reads back to the genome. Then, we

used Benchmarking Universal Single-Copy Orthologs v3.0.1 (BUSCO) [17] to evaluate the completeness of the genome assembly by searching for annotated genes in the assembly.

Genome scaffolding with BioNano auxiliary assembly

High-molecular-weight DNA was isolated from the whole body (excluding the gut) and then labeled with Labeling Master Mix and DLE-1; next, the DNA was imaged automatically with a BioNano Saphyr system. BioNano raw BNX files were *de novo* assembled into genome maps with BioNano Solve (http://bnxinstall.com/solve/BionanoSolveInstall.html). The sorted and autodenoised single molecules were subjected to pairwise comparisons by RefAligner (https://bionanogenomics.com/wp-content/uploads/2017/03/RefAligner.msi_.zip) to identify molecule overlaps, and consensus maps were constructed. All molecules were then mapped back to the consensus maps, and the maps were recursively refined and extended (two times).

Chromosomal-level genome assembly by Hi-C

The Hi-C library was prepared following a previously described procedure [18] with some modifications. Briefly, fresh whole-body samples (excluding the gut) were cut into 2-cm pieces and treated with 1% formaldehyde for 10 min at room temperature to induce crosslinking. The reaction was quenched by adding 2.5 M glycine to a final concentration of 0.2 M for 5 min. Nuclei were digested, marked, and ligated using 100 U *Dpn*II, biotin-14-dCTP (Invitrogen, Carlsbad, CA, USA), and T4 DNA Ligase, respectively. After incubation overnight for reverse crosslinking, the ligated DNA was sheared into 300–600-bp fragments. The DNA fragments were blunt-end repaired and A-tailed, followed by purification through biotin-streptavidin-mediated pulldown. Finally, the Hi-C libraries were quantified and sequenced using an Illumina Hiseq platform (Illumina).

In total, 556 million paired-end reads were generated from the Hi-C libraries. Low-quality reads (quality scores less than 15), adaptor sequences, N ratio greater than 5% reads, and reads shorter than 30-bp were filtered out using fastp v0.12.6 [19], and the clean paired-end reads were then mapped to the draft assembled sequence using bowtie2 v2.3.2 [20] to yield unique mapped paired-end reads.

As a result, 141 million uniquely mapped pair-end reads were generated, of which 76.82% were valid interaction pairs. Combined with the valid Hi-C data, we subsequently used the LACHESIS (ligating adjacent chromatin enables scaffolding *in situ*) [21] *de novo* assembly pipeline to produce chromosome-level sequences with the following parameters: (1) CLUSTER MIN. RE SITES = 150; (2) CLUSTER MAX. LINK DENSITY = 2.5; (3) CLUSTER NONINFORMATIVE RATIO = 1.4; (4) ORDER MIN. N RES. IN TRUNK = 60; and (5) ORDER MIN. N RES. IN SHREDS = 60.

We used the same method to assemble the genome of a published male *G. affinis* (https://www.ncbi.nlm.nih.gov/assembly/GCA_003097735.1) and obtained chromosomal-level genomic data.

Annotation of repetitive elements

Simple sequence repeat (SSR) sequences in the genome were analyzed by MISA [22]. For transposable elements (TEs), we first used RepeatModeler (www.repeatmasker.org/RepeatModeler/), LTR_FINDER [23], and MITE-Hunter software [24], based on the principle of *de novo* methods and TE-specific architecture to build a *G. affinis* TE sequence library. The data were then combined with Repbase [25] to construct the final database. Finally, RepeatMasker software (www.repeatmasker.org) was used to predict the TEs in male and female *G. affinis* according to the final constructed TE database.

Gene prediction and function annotation

First, for homology-based prediction, the RNA-seq bam file from mapped reads to the genome by HISAT2 [26] and protein sequences from five sequenced vertebrates, *Danio rerio* (ftp://ftp.ensembl.org/pub/release-96/fasta/danio_rerio/dna/), *Oryzias latipes* (ftp://ftp.ensembl.org/pub/release-96/fasta/oryzias_latipes_hni/dna/), *Nothobranchius furzeri* (http://nfingb.leibniz-fli.de/), *Xiphophorus maculatus* (ftp://ftp.ensembl.org/pub/release-96/fasta/xiphophorus_maculatus/dna/), and *Poecilia formosa* (ftp://ftp.ensembl.org/pub/release-96/fasta/poecilia_formosa/dna/) were used to predict *G. affinis* genes by GeMoMa [27]. Second, we used Augustus for *ab initio* prediction, a training set generated from the GeMoMa results and transcripts of *G. affinis*, and transcripts obtained from high-throughput data using HISAT2 combined with Stringtie [28]. Full-length transcriptome data were used to construct consensus sequences through clustering with IsoSeq3 (https://github.com/PacificBiosciences/IsoSeq3). These sequences were then compared with reference genomes using GMAP [29]; next, both transcripts were integrated to remove redundancy, and the results were then processed with PASA to obtain the final results. Augustus' predictions were compared with the Pfam database (http://pfam.sanger.ac.uk/) to remove genes without domains, and the results of Augustus and GeMoMa were further removed by alternative splicing and integrated. Finally, the TransposonPSI (http://transposonpsi.sourceforge.net/) alignment was used to remove sequences containing transposons, yielding the final results.

Functional annotation of the predicted genes of *G. affinis* was performed by alignment to the SwissProt (https://www.uniprot.org/), TrEMBL (https://www.uniprot.org/), Kyoto Encyclopedia of Genes and Genomes (KEGG; https://www.genome.jp/kegg/), and gene ontology (GO) databases using BLAST (https://blast.ncbi.nlm.nih.gov/Blast.cgi) and KAAS (v2.1) [30]. Motifs and domains were annotated using InterProScan v5.24 [31].

Noncoding RNA prediction

rRNAs, small nuclear RNAs (snRNAs), microRNAs (miRNAs), and tRNAs, were identified by adopting Infernal v1.1.2 [32] using the Rfam database (release 13.0) [33] for the *G. affinis* genome using BLASTN (E-value ≤ 1e−5) [34]. tRNAs were predicted using tRNAscan-SE v1.3.1 software [35] with default parameters for eukaryotes. The rRNAs and their subunits were predicted using RNAmmer v1.2 [36].

Evolutionary and comparative genomic analyses

We use OrthoMCL (version 2.0.9) [37] to cluster the female *G. affinis* annotated genes with an e-value cutoff of 1e–5 and Markov Chain Clustering with default inflation parameters for an all-to-all BLASTP analysis of entries for the reference genomes of 11 fishes, including *G. affinis* in this study and other 10 published fishes reported to date (*P. reticulate*, *P. formosa*, *P. latipinna*, *P. mexicana*, *X. couchianus*, *N. furzeri*, *Cyprinodon variegatus*, *Fundulus heteroclitus*, *Lepisosteus oculatus*, and *Oreochromis niloticus*). Computational Analysis of gene Family Evolution (CAFE; version 4.0.1) [38] was used to identify expanded and contracted gene families in *G. affinis*, and these data were then used for GO and KEGG enrichment analyses.

The orthologous genes obtained from the above analyses was subjected to multiple sequence alignment using Mafft (v7.313) (https://mafft.cbrc.jp/alignment/software/) and Gblocks v0.91b (http://molevol.cmima.csic.es/castresana/Gblocks.html) to extract conserved sites based on the GTRGAMMA model and RAxML (v 8.2.11) [39]. Using this tree, MCMCTREE in PAML v4.9e [40] was applied to estimate the 95% confidence intervals of the differentiation times, where the published timings for the divergence of difference species were obtained with the TimeTree database (http://www.timet ree.org/).

The orthologous genes were then used in PAML codon substitution models and likelihood ratio tests (codeml) based on the branch-site model to calculate Ka and Ks, yielding positively selected genes, which were then utilized for GO and KEGG enrichment analyses.

Recognition and comparison of the W and Z chromosomes of G. affinis

Mummer [41] was used for aligning entire genomes from *X. couchianus*, and male and female *G. affinis*, to make the chromosome numbering system of both species the same. The W chromosome was identified by the specificity of the female *G. affinis* W chromosome length, and then, the Z chromosome in the male *G. affinis* was identified based on synteny. Mummer [41] was also used for aligning entire genomes from the Z and W chromosomes, and circos plot distributions of homologous sequence pairs among the Z and W chromosome pairs were plotted using Circos [42].

According to the results of the RepeatMasker analysis of female and male *G. affinis* genomes, the length and distribution of TEs on chromosomes Z and W were compared. OrthoFinder [43] was used to compare the genes on chromosomes Z and W.

TE insertion time analyses

We calculated female and male *G. affinis* TE insertion times in genomes using the algorithm T = K / 2r, where K is the Kimura distance-based copy divergence of TEs and r is the nucleic acid substitution rate. The K value was obtained from RepeatMasker. To estimate r values for *G. affinis*, we used LASTZ (v1.04.00) (http://www.bx.psu.edu/~rsharris/lastz/), chainNet (v2) [44], and MULTIZ (v11.2) [45], along with genomes used in evolutionary analyses and the female *G. affinis* genome as a reference sequence. With the whole-genome alignments, we used the msa_view tool in the PHAST package (v1.2.1) [46] to extract 4D site alignments, based on the female *G. affinis* gene annotations. The phyloFit program in the PHAST package

was used to estimate the phylogenetic model, with tree topology (result of evolutionary analyses) as an input parameter. The branch length results were represented as units of substitutions per site. We calculated the root-to-tip substitution rates from the most recent common ancestor of selected species to each fish lineage, and then divided the root-to-tip substitution rates by the divergence time (Divergence time: 314.47 Mya, 314.47 Million years ago) of most recent common ancestor of selected species.

Results and Discussion

Female G. affinis genome initial characteristics

A total of 30.5 Gb Illumina clean reads were used for analyzing female *G. affinis* genome size and heterozygosity using *K-mer* analysis. Based on 26,474,864,304 *17-mers* and a peak *17-mer* depth of 37 (Figure S1), the estimated genome size of female *G. affinis* was about 715 Mb, which is similar to that of the nuclear DNA content estimated in a previous study using flow cytometry (0.75 pg, approximately 733 Mb) [47], and the estimated heterozygosity rate was approximately 0.42%.

De novo assembly of a female G. affinis reference genome

Next, 74.4 Gb (2,866,145 reads, average read length of 25.98 Kb, N50 35.86 Kb, and longest read length of 273 Kb) Oxford nanopore clean long reads were used to construct the reference genome. We obtained a 662-Mb genome by assembly with a contig N50 length of 12.9 Mb.

The genome consisted of 217 contigs, and the longest contig was 28.6 Mb. Then, BUSCO [17] was used to assess the completeness of the assembled genome. Approximately 97.2% of the complete genes could be detected in the genome of female *G. affinis*, confirming the completeness of the genome. Assembly results of long reads scaffolds obtained using optical maps were assembled with 80-Gb BioNano molecules. The final assembly contained 125

scaffolds with a scaffold N50 size of 26.4 Mb. Finally, we used the Hi-C technique to anchor the assembly scaffolds in 24 chromosomes of female *G. affinis* (Table S1). We found that 141,528,181 unique mapped paired-end reads were generated and occupied approximately 51.5% of the total clean paired-end reads (274,658,176). Then, the frequency of scaffold interactions was estimated on the basis of the pairs mapped to the scaffolds. We found that 113 scaffolds were successfully anchored in 24 chromosomes (Figure 1, Figure S2a), consistent with the records of the chromosome number by cytogenetic analysis [10, 48], representing 90.4% of all scaffolds and 97.9% of all genome nucleotide bases. The total assembly size of the chromosomes was approximately 679.4 Mb (Table 1). In the male *G. affinis* genome, 734 scaffolds were successfully anchored in 24 chromosomes (Figure 1, Figure S2b), and the total assembly size was 592.7 Mb.

Genome annotation

Assembled chromosome-level genome of female *G. affinis* were used to predict repeat sequences. In total, 5,630,271 SSRs were identified, including 5,478,552 mono-, 100,272 di-, 28,431 tri-, 19,721 tetra-, 2,048 penta-, and 1,247 hexa-nucleotide repeats. Overall, the combined the homology-based and *de novo* prediction results indicated that TEs accounted for 22.54% of the genome (Table S2). Additionally, class I TEs (RNA transposons) occupied approximately 5.15% of the assembly genome. The most abundant RNA transposons found in the *G. affinis* genome were long interspersed nuclear elements, which constituted 54.37% of all identified RNA transposons. The female *G. affinis* genome was very rich in class II TEs (DNA transposons), which occupied almost 11.83% of genome content.

For genome annotation, 23,997 protein-coding genes were predicted in the female *G. affinis* genome. Compared with other existing published poeciliid fish annotated information, a number of genes in female *G. affinis* was similar to those in *P. formosa* (23,615 genes) and

X. maculatus (23,628 genes) (Table S3 and Figure S3). BUSCO gene prediction was carried out using the actinopterygii_odb9 single-copy homologous gene to predict the existing sequence of the genome. Approximately 97% of complete gene components could be found in this gene set, indicating that most of the conserved genes were well predicted and that the prediction results were relatively reliable (Table S4). Finally, 23,737 genes were annotated in at least one of the databases (KOG, KEGG, NR, SwissProt, GO), and up to 98.92% of *G. affinis* genes were functionally annotated (Table S5). Finally, 143 snRNAs, 220 rRNAs, 371 miRNAs, and 3885 tRNAs were also identified.

Genome evolution

In order to determine the evolutionary relationships between *G. affinis* and other vertebrates, a phylogenetic tree was reconstructed on the basis of 6,457 single-copy orthologous genes from 10 other vertebrate genomes (Figure 2a). *L. oculatus* and *O. niloticus* were used as outgroups. As a species of the family Poeciliidae, *G. affinis* clustered into one branch with other fish from Poeciliidae. Compared with six other sequenced members of the Poeciliidae family, *G. affinis* had a closer relationship with *X. couchianus*, consistent with previously published phylogenies [49]. Next, a timetree was created based on the above 6,457 single-copy orthologous genes, and the estimated divergence time between *G. affinis* and *X. couchianus* was found to be approximately 16.57 million years ago (Mya; Figure 2; Figure S3). In addition, the divergence time between *G. affinis* and four other members of the Poeciliidae family was approximately 22.75 Mya.

In order to examine the evolutionary history of gene families, we performed gene family expansion and contraction analysis with the female *G. affinis* genome. We found 652 expansion gene families and 1,046 contraction gene families (Figure 2b). Expansion gene families were enriched in 44 GO (Table S6) categories and 34 KEGG pathways (Table S7), most of which

were related to oxygen metabolism, olfactory pathways, and visual pathways. Next, codeml was used to calculate the average Ka/Ks values and conduct branch-site likelihood ratio analyses to detect positively selected genes in the female *G. affinis* genome. The results showed that there were 590 positively selected genes in the female *G. affinis* genome. The positively selected genes were enriched in 12 GO categories (aspartic-type endopeptidase activity, DNA repair, microtubule binding, insulin-like growth factor binding, tRNA aminoacylation for protein translation, microtubule motor activity, rRNA processing, microtubule-based movement, protein dephosphorylation, protein tyrosine phosphatase activity, chromatin binding, and nucleus) and three KEGG pathways (complement and coagulation cascades, peroxisome, and platelet activation).

Recognition and evolution of sex chromosomes

Sex determination systems in fish are variable, ranging from XX/XY to ZZ/ZW [50]. Fish generally do not have highly morphologically differentiated sex chromosomes, making it difficult to distinguish between autosomes and sex chromosomes. Hence, there are only a few fish species for which there is known information on sex determination mechanisms and sex chromosome systems. Therefore, a suitable experimental model is required for the identification and elucidation of the mechanisms of fish sex chromosome evolution, and the female *G. affinis* is a suitable and consistent model.

Early karyotype analysis demonstrated that female *G. affinis* shows heterogamy of the ZW type, and its W chromosome is much longer than other chromosomes [10, 48]. The longest sequence was selected from the assembly results at the chromosome level as the W candidate chromosome, and one female-specific DNA marker [51] was used for confirmation. In the end, the results showed that the marker could be aligned to the W candidate chromosome, but was not found in the genome of the male *G. affinis*. Analysis of the synteny of the whole genomes

of female and male mosquitofish by Mummer [41] showed that the Z chromosome was also present in the male *G. affinis* genome (Figure 1). By comparing the Z and W chromosomes (Figure 3a), we found the length of the W and Z chromosome repeat sequences to be approximately 5.0 Mb and 8.5 Mb, respectively. Among them, the length and content of the *Helitron* superfamily of the two chromosomes (Z: 591,639 bp, 1.3%; W: 6,6868 bp, 0.24%) were significantly different (The results had submitted to GigaDB). There were 1279 and 1027 genes on the W and Z chromosomes, respectively. Homologous analysis showed that there were 712 one-to-one pairs. There were 118 and 203 genes on the W and Z chromosomes unassigned to any gene groups, and the others were of the one-to-many and many-to-many types; these results provide research directions for our future analyses on functional genomics (the results have been submitted to GigaDB).

Some researchers have studied the role of transposons in sex chromosome differentiation, and they found that TEs were shown to be play an important role in the evolution of sex chromosomes, with their accumulation and loss having huge effects on the lengths of sex chromosomes [52-54]. However, differences in TE contents between Z and W chromosomes alone cannot determine the true course of differentiation, e.g., whether the longer length of the W chromosome compared with the Z chromosome is caused by extension of the W chromosome or by degeneration of the Z chromosome. There is no substantial evidence to explain this observation. Therefore, the introduction of the time factor is extremely important. *G. holbrooki* and *G. affinis* are so closely related that for a long time, biologists thought they were the same species. Phylogenetic analyses estimated that their divergence time was approximately 2–7 Mya [51, 55, 56], and other researchers showed that the XY and ZW sex determination mechanisms had independent origins in *G. holbrooki* and *G. affinis,* respectively [50]. Therefore, we speculate that the differentiation of Z and W sex chromosomes is a very recent event. Additionally, previous studies suggested that this process may be enriched on the

W chromosome by TEs, leading to an increase in the sex chromosome size during the early phase of differentiation and the subsequent reduction in size later during evolution [57]. If this hypothesis is correct, then we should be able to observe a large number of transposon insertions in the W chromosome in the recent past (later than 2–7 Mya). Indeed, our results showed the presence of very recent mass insertion events of TEs on the W chromosome (Figure 3b), and the insertion time characteristics of the TEs on the W chromosome were specific because the insertion time trends of autosomal and Z-chromosomal TEs differed dramatically (Figure S5). Moreover, we speculate that most of the long gaps (Figure 3a) on the W chromosome were also caused by the aggregation of too many highly similar TE sequences to form TE clusters through the recent activation of TEs. Thus, we expected that the TE content of the W chromosome of *G. affinis* should be much higher than that observed till date. Accordingly, our results showed that the cause of sex chromosome differentiation in female *G. affinis* was likely to be related to extension of the W chromosome.

Conclusions

In this study, we assembled the chromosome-level female western mosquitofish genome using the most mainstream technology available. In terms of parameters such as contig N50, scaffold N50, and gene annotation number, these are high-quality genomic data. Evolutionary analysis provides some ideas for future work, for example, oxygen transport in mosquitofish deserves attention. We conducted a preliminary study on the sex chromosome based on the specificity of the sex chromosome in female western mosquitofish, and provided data to support the previous hypothesis that a longer W chromosome is associated with the activity (insertion) of TEs. In conclusion, the high-quality genomic data generated in this study lay the foundation for the study of chromosome evolution, reproductive characteristics, and sexual dimorphism in western mosquitofish.

Availability of supporting data

The raw genome and RNA sequencing data were deposited in the SRA under Bioproject number PRJNA599452. The chromosome-level genome, annotation, and other supporting data are also available via the GigaScience database, *GigaDB*.

Abbreviations

BLAST: Basic Local Alignment Search Tool; bp: base pairs; BUSCO: Benchmarking Universal Single-Copy Orthologs; BWA: Burrows-Wheeler Aligner; CAFE: Computational Analysis of gene Family Evolution ; GeMoMa: Gene Model Mapper; Gb: gigabase pairs; GO: Gene Ontology; Hi-C: High-throughput chromosome conformation capture; KASS: KEGG Automatic Annotation Server; kb: kilobase pairs; KEGG: Kyoto Encyclopedia of Genes and Genomes; Mb: megabase pairs; MRCA: most recent common ancestor; LTR: long terminal repeat; NCBI: National Center for Biotechnology Information; PAML: Phylogenetic Analysis by Maximum Likelihood; PASA: Program to Assemble Spliced Alignments; RAxML: Randomized Axelerated Maximum Likelihood; RNA-seq: RNA sequencing; TE: transposable element.

Competing Interests

The authors declare that there have no competing interests.

Author Contributions

F.S. performed the major part of data analysis and drafted the manuscript. Y.M. and N.L. contributed to samples collection and drafted the manuscript. Z.P. and L.A. contributed to research design and final edits to the manuscript. All authors read and approved the final manuscript.

Acknowledgements

We are grateful to Drs. Aurélie Kapusta, Guojie Zhang, and Cai Li for help with estimating substitution rate and transposon insertion time. We also thank Drs. Wen Wang, Lei Chen, Kun Wang, Ru Zhang, Botong Zhou, Zeshan Lin, and Bao Wang for discussion. This work was supported by the grants from the National Key Research and Development Program of China (2018YFD0900805), the National Natural Science Foundation of China (31872204), the Fundamental Research Funds for the Central Universities (XDJK2020C043), and the Natural Science Foundation of Chongqing Postdoctoral Science Foundation (cstc2019jcyj-bshX0071).

References

1. Russo G, Chou A, Rettig JE and Smith GR. Foraging responses of mosquitofish (*Gambusia affinis*) to items of different sizes and colors. J Freshw Ecol. 2008;23 4:677-8. doi:10.1080/02705060.2008.9664256.
2. Cote J, Fogarty S, Weinersmith K, Brodin T and Sih A. Personality traits and dispersal tendency in the invasive mosquitofish (*Gambusia affinis*). Proc Biol Sci. 2010;277 1687:1571-9. doi:10.1098/rspb.2009.2128.
3. Smith GR and Smith LE. Effects of Western Mosquitofish (*Gambusia affinis*) on tadpole production of Gray Treefrogs (*Hyla versicolor*). Herpetol Conserv Biol. 2015;10 2:723-7.
4. Merkley SS, Rader RB and Schaalje GB. Introduced Western Mosquitofish (*Gambusia affinis*) reduce the emergence of aquatic insects in a desert spring. Freshw Sci. 2015;34 2:564-73. doi:10.1086/680381.
5. Raut SA and Angus RA. Triclosan has endocrine- disrupting effects in male western mosquitofish, *Gambusia affinis*. Environ Toxicol Chem. 2010;29 6:1287-91. doi:10.1002/etc.150.
6. Brockmeier EK, Ogino Y, Iguchi T, Barber DS and Denslow ND. Effects of 17beta-trenbolone on Eastern and Western mosquitofish (*Gambusia holbrooki* and *G. affinis*) anal fin growth and gene expression patterns. Aquat Toxicol. 2013;128-129:163-70. doi:10.1016/j.aquatox.2012.12.007.
7. Vera M, Díez- del- Molino D and García- Marín JL. Genomic survey provides insights into the evolutionary changes that occurred during European expansion of the invasive mosquitofish (*Gambusia holbrooki*). Mol Ecol. 2016;25 5:1089-105. doi:10.1111/mec.13545.

8. Diez-del-Molino D, Carmona-Catot G, Araguas RM, Vidal O, Sanz N, Garcia-Berthou E, et al. Gene flow and maintenance of genetic diversity in invasive mosquitofish (*Gambusia holbrooki*). PLoS ONE. 2013;8 12:e82501. doi:10.1371/journal.pone.0082501.

9. Vidal O, Sanz N, Araguas RM, Fernández- Cebrian R, Diez- del- Molino D and García- Marín JL. SNP diversity in introduced populations of the invasive *Gambusia holbrooki*. Ecol Freshw Fish. 2012;21 1:100-8. doi:10.1111/j.1600- 0633.2011.00527.x.

10. Black DA and Howell WM. The North American mosquitofish, *Gambusia affinis*: a unique case in sex chromosome evolution. Copeia. 1979:509-13. doi:10.2307/1443231.

11. Irwin DE. Sex chromosomes and speciation in birds and other ZW systems. Mol Ecol. 2018;27 19:3831-51. doi:10.1111/mec.14537.

12. Hoffberg SL, Troendle NJ, Glenn TC, Mahmud O, Louha S, Chalopin D, et al. A High-Quality Reference Genome for the Invasive Mosquitofish *Gambusia affinis* Using a Chicago Library. G3-Genes Genom Genet. 2018;8 6:1855-61. doi:10.1534/g3.118.200101.

13. Marcais G and Kingsford C. A fast, lock-free approach for efficient parallel counting of occurrences of *k-mers*. Bioinformatics. 2011;27 6:764-70. doi:10.1093/bioinformatics/btr011.

14. Ruan J and Li H. Fast and accurate long-read assembly with wtdbg2. Nat Methods. 2019. doi:10.1038/s41592-019-0669-3.

15. Li H and Durbin R. Fast and accurate short read alignment with Burrows-Wheeler transform. Bioinformatics. 2009;25 14:1754-60. doi:10.1093/bioinformatics/btp324.

16. Walker BJ, Abeel T, Shea T, Priest M, Abouelliel A, Sakthikumar S, et al. Pilon: an integrated tool for comprehensive microbial variant detection and genome assembly improvement. PLoS ONE. 2014;9 11:e112963. doi:10.1371/journal.pone.0112963.

17. Simao FA, Waterhouse RM, Ioannidis P, Kriventseva EV and Zdobnov EM. BUSCO: assessing genome assembly and annotation completeness with single-copy orthologs. Bioinformatics. 2015;31 19:3210-2. doi:10.1093/bioinformatics/btv351.

18. Belton JM, McCord RP, Gibcus JH, Naumova N, Zhan Y and Dekker J. Hi-C: a comprehensive technique to capture the conformation of genomes. Methods. 2012;58 3:268-76. doi:10.1016/j.ymeth.2012.05.001.

19. Chen S, Zhou Y, Chen Y and Gu J. fastp: an ultra-fast all-in-one FASTQ preprocessor. Bioinformatics. 2018;34 17:i884-i90. doi:10.1093/bioinformatics/bty560.

20. Langmead B and Salzberg SL. Fast gapped-read alignment with Bowtie 2. Nat Methods. 2012;9 4:357-9. doi:10.1038/nmeth.1923.

21. Burton JN, Adey A, Patwardhan RP, Qiu R, Kitzman JO and Shendure J. Chromosome-scale scaffolding of de novo genome assemblies based on chromatin interactions. Nat Biotechnol. 2013;31 12:1119-25. doi:10.1038/nbt.2727.

22. Beier S, Thiel T, Munch T, Scholz U and Mascher M. MISA-web: a web server for microsatellite prediction. Bioinformatics. 2017;33 16:2583-5. doi:10.1093/bioinformatics/btx198.

23. Xu Z and Wang H. LTR_FINDER: an efficient tool for the prediction of full-length LTR retrotransposons. Nucleic Acids Res. 2007;35:W265-8. doi:10.1093/nar/gkm286.

24. Han Y and Wessler SR. MITE-Hunter: a program for discovering miniature inverted-repeat transposable elements from genomic sequences. Nucleic Acids Res. 2010;38 22:e199. doi:10.1093/nar/gkq862.

25. Bao W, Kojima KK and Kohany O. Repbase Update, a database of repetitive elements in eukaryotic genomes. Mob DNA. 2015;6:11. doi:10.1186/s13100-015-0041-9.

26. Kim D, Langmead B and Salzberg SL. HISAT: a fast spliced aligner with low memory requirements. Nat Methods. 2015;12 4:357-60. doi:10.1038/nmeth.3317.

27. Keilwagen J, Wenk M, Erickson JL, Schattat MH, Grau J and Hartung F. Using intron position conservation for homology-based gene prediction. Nucleic Acids Res. 2016;44 9:e89-e. doi:10.1093/nar/gkw092.

28. Pertea M, Pertea GM, Antonescu CM, Chang TC, Mendell JT and Salzberg SL. StringTie enables improved reconstruction of a transcriptome from RNA-seq reads. Nat Biotechnol. 2015;33 3:290-5. doi:10.1038/nbt.3122.

29. Wu TD and Watanabe CK. GMAP: a genomic mapping and alignment program for mRNA and EST sequences. Bioinformatics. 2005;21 9:1859-75. doi:10.1093/bioinformatics/bti310.

30. Moriya Y, Itoh M, Okuda S, Yoshizawa AC and Kanehisa M. KAAS: an automatic genome annotation and pathway reconstruction server. Nucleic Acids Res. 2007;35:W182-5. doi:10.1093/nar/gkm321.

31. Jones P, Binns D, Chang HY, Fraser M, Li W, McAnulla C, et al. InterProScan 5: genome-scale protein function classification. Bioinformatics. 2014;30 9:1236-40. doi:10.1093/bioinformatics/btu031.

32. Nawrocki EP and Eddy SR. Infernal 1.1: 100-fold faster RNA homology searches. Bioinformatics. 2013;29 22:2933-5. doi:10.1093/bioinformatics/btt509.

33. Kalvari I, Argasinska J, Quinones-Olvera N, Nawrocki EP, Rivas E, Eddy SR, et al. Rfam 13.0: shifting to a genome-centric resource for non-coding RNA families. Nucleic Acids Res. 2018;46 D1:D335-D42. doi:10.1093/nar/gkx1038.

34. Camacho C, Coulouris G, Avagyan V, Ma N, Papadopoulos J, Bealer K, et al. BLAST+: architecture and applications. BMC Bioinformatics. 2009;10:421. doi:10.1186/1471-2105-10-421.

35. Lowe TM and Eddy SR. tRNAscan-SE: a program for improved detection of transfer RNA genes in genomic sequence. Nucleic Acids Res. 1997;25 5:955-64. doi:10.1093/nar/25.5.955.
36. Lagesen K, Hallin P, Rodland EA, Staerfeldt HH, Rognes T and Ussery DW. RNAmmer: consistent and rapid annotation of ribosomal RNA genes. Nucleic Acids Res. 2007;35 9:3100-8. doi:10.1093/nar/gkm160.
37. Li L, Stoeckert CJ, Jr. and Roos DS. OrthoMCL: identification of ortholog groups for eukaryotic genomes. Genome Res. 2003;13 9:2178-89. doi:10.1101/gr.1224503.
38. De Bie T, Cristianini N, Demuth JP and Hahn MW. CAFE: a computational tool for the study of gene family evolution. Bioinformatics. 2006;22 10:1269-71. doi:10.1093/bioinformatics/btl097.
39. Stamatakis A. RAxML version 8: a tool for phylogenetic analysis and post-analysis of large phylogenies. Bioinformatics. 2014;30 9:1312-3. doi:10.1093/bioinformatics/btu033.
40. Yang Z. PAML: a program package for phylogenetic analysis by maximum likelihood. Comput Appl Biosci. 1997;13 5:555-6. doi:10.1093/bioinformatics/13.5.555.
41. Kurtz S, Phillippy A, Delcher AL, Smoot M, Shumway M, Antonescu C, et al. Versatile and open software for comparing large genomes. Genome Biol. 2004;5 2:R12. doi:10.1186/gb-2004-5-2-r12.
42. Krzywinski M, Schein J, Birol I, Connors J, Gascoyne R, Horsman D, et al. Circos: an information aesthetic for comparative genomics. Genome Res. 2009;19 9:1639-45. doi:10.1101/gr.092759.109.
43. Emms DM and Kelly S. OrthoFinder: phylogenetic orthology inference for comparative genomics. Genome Biol. 2019;20 1:238. doi:10.1186/s13059-019-1832-y.

44. Kent WJ, Baertsch R, Hinrichs A, Miller W and Haussler D. Evolution's cauldron: duplication, deletion, and rearrangement in the mouse and human genomes. Proc Natl Acad Sci U S A. 2003;100 20:11484-9. doi:10.1073/pnas.1932072100.

45. Blanchette M, Kent WJ, Riemer C, Elnitski L, Smit AF, Roskin KM, et al. Aligning multiple genomic sequences with the threaded blockset aligner. Genome Res. 2004;14 4:708-15. doi:10.1101/gr.1933104.

46. Hubisz MJ, Pollard KS and Siepel A. PHAST and RPHAST: phylogenetic analysis with space/time models. Brief Bioinform. 2011;12 1:41-51. doi:10.1093/bib/bbq072.

47. Tiersch TR, Chandler RW, Wachtel SS and Elias S. Reference-Standards for Flow-Cytometry and Application in Comparative Studies of Nuclear-DNA Content. Cytometry. 1989;10 6:706-10. doi:10.1002/cyto.990100606.

48. Chen T and Ebeling A. Karyological evidence of female heterogamety in the mosquitofish, *Gambusia affinis*. Copeia. 1968:70-5. doi:10.2307/1441552.

49. Furness AI, Pollux BJA, Meredith RW, Springer MS and Reznick DN. How conflict shapes evolution in poeciliid fishes. Nat Commun. 2019;10 1:3335. doi:10.1038/s41467-019-11307-5.

50. Kottler VA, Feron R, Nanda I, Klopp C, Du K, Kneitz S, et al. Independent Origin of XY and ZW Sex Determination Mechanisms in Mosquitofish Sister Species. Genetics. 2020;214 1:193-209; doi:10.1534/genetics.119.302698.

51. Lamatsch DK, Adolfsson S, Senior AM, Christiansen G, Pichler M, Ozaki Y, et al. A transcriptome derived female-specific marker from the invasive Western mosquitofish (*Gambusia affinis*). PLoS ONE. 2015;10 2:e0118214. doi:10.1371/journal.pone.0118214.

52. Rosolen LAM, Vicari MR and Almeida MC. Accumulation of Transposable Elements in Autosomes and Giant Sex Chromosomes of *Omophoita* (Chrysomelidae: Alticinae). Cytogenet Genome Res. 2018;156 4:215-22. doi:10.1159/000495199.

53. Erlandsson R, Wilson JF and Paabo S. Sex chromosomal transposable element accumulation and male-driven substitutional evolution in humans. Mol Biol Evol. 2000;17 5:804-12. doi:10.1093/oxfordjournals.molbev.a026359.

54. Dechaud C, Volff JN, Schartl M and Naville M. Sex and the TEs: transposable elements in sexual development and function in animals. Mob DNA. 2019;10:42. doi:10.1186/s13100-019-0185-0.

55. Lydeard C, Wooten MC and Meyer A. Molecules, morphology, and area cladograms: a cladistic and biogeographic analysis of *Gambusia* (Teleostei: Poeciliidae). Syst Biol. 1995;44 2:221-36. doi:10.1093/sysbio/44.2.221.

56. Helmstetter AJ, Papadopulos AS, Igea J, Van Dooren TJ, Leroi AM and Savolainen V. Viviparity stimulates diversification in an order of fish. Nat Commun. 2016;7:11271. doi:10.1038/ncomms11271.

57. Schartl M, Schmid M and Nanda I. Dynamics of vertebrate sex chromosome evolution: from equal size to giants and dwarfs. Chromosoma. 2016;125 3:553-71. doi:10.1007/s00412-015-0569-y.

Table 1. Genome assembly statistics of *Gambusia affinis*.

	Nanopore	BioNano	Hi-C (♀)	Hi-C (♂)
Total assembly size of contig/scaffold/chromosome (bp)	662,579,534	680,140,492	679,423,294*	592,666,412*
Number of contig/scaffold/chromosome	217	125	24	24
N50 contig/scaffold/chromosome length (bp)	12,906,370	26,455,434	29,761,488	25,946,590
N90 contig/scaffold/chromosome length (bp)	1,629,223	18,394,109	23,709,503	21,272,223
Longest contig/scaffold/chromosome (bp)	28,665,999	31,542,956	45,125,082	30,583,032

* The length of 24 chromosomes, excluding the length of the unanchored sequences.

Figure Legends

Figure 1. Genomic synteny of *X. couchianus*, female *G. affinis*, and male *G. affinis*. Female *G. affinis* LG01 represents the W chromosome and male *G. affinis* LG01 represents the Z chromosome.

Figure 2. Phylogenetic and evolutionary analysis of *G. affinis.* (a) Divergence time estimates and gene clusters in *G. affinis* and other species. (b) Expansion and contraction of *G. affinis* gene families. MRCA represents most recent common ancestor, and circle diagrams and numbers below represent the proportion and specific values of the gene families of expansion and contraction, respectively (red represents contraction, green represents expansion).

Figure 3. Comparative genomic analysis of the Z and W chromosomes. (a) Circos plot of Z and W chromosome alignment; the red region represents the repeat sequence density, and the green region represents the GC density. (b) Distribution of the transposon activity time for the W chromosome. (c) Distribution of transposon activity time for the Z chromosome. The x-coordinate represents the insertion time of TEs, and the y-coordinate represents the insertion length of TEs.

Additional files

Table S1: Result of female and male *Gambusia affinis* genomic assembly at chromosome-level.

Table S2: Transposable elements (TEs) annotation in the female *Gambusia affinis* genome.

Table S3: Comparative analysis of the annotated gene set of female *Gambusia affinis* with those of five teleosts.

Table S4: Assessment of female *Gambusia affinis* genome completeness by BUSCO.

Table S5: Statistics for gene function annotation in female *Gambusia affinis* genome.

Table S6: Expansion gene families of female *Gambusia affinis* were enriched in 44 GO categories.

Table S7: Expansion gene families of female *Gambusia affinis* were enriched in 34 KEGG pathways.

Figure S1: Frequency distribution of the 17-mer graph analysis used to estimate the size of female *Gambusia affinis*.

Figure S2: Western mosquitofish genome scaffold contact matrix using Hi-C data. (a) Female western mosquitofish. (b) Male western mosquitofish. The color bar indicates the contact density from red (high) to white (low).

Figure S3: The comparisons of CDS length, exon length, exon-number, gene length, intro length, and intron number in the genomes of female *Gambusia affinis* and other teleosts.

Figure S4: Divergence time of *Gambusia affinis* and other fish species.

Figure S5: Distribution of transposon activity time for different autosomes of female *Gambusia affinis*. The x-coordinate is the insertion time of TEs, and the y-coordinate is the insertion length of TEs.

Figure 1

Click here to download Figure Figure_1.png

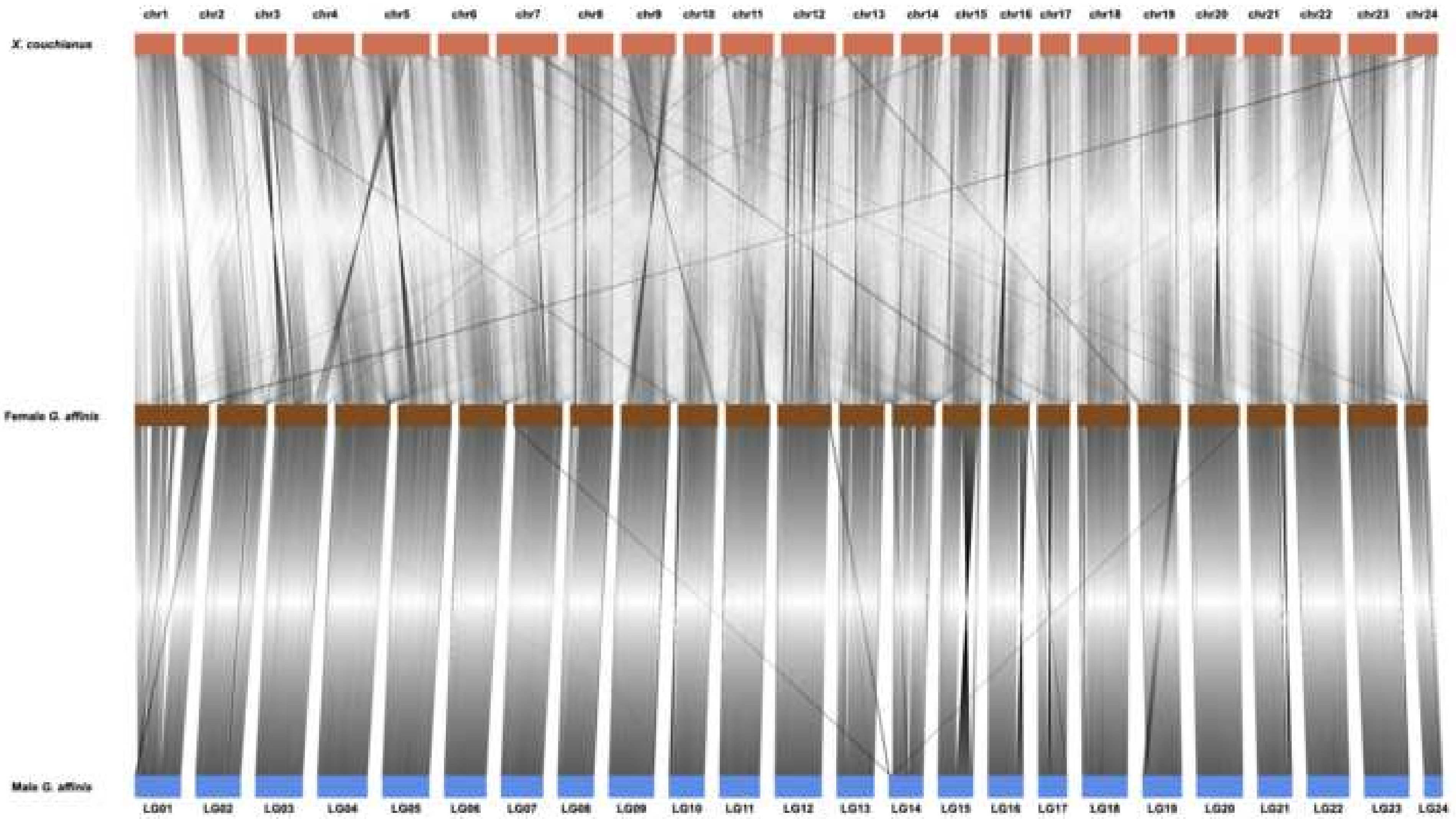

Figure 2

Click here to download Figure Figure_2.pdf

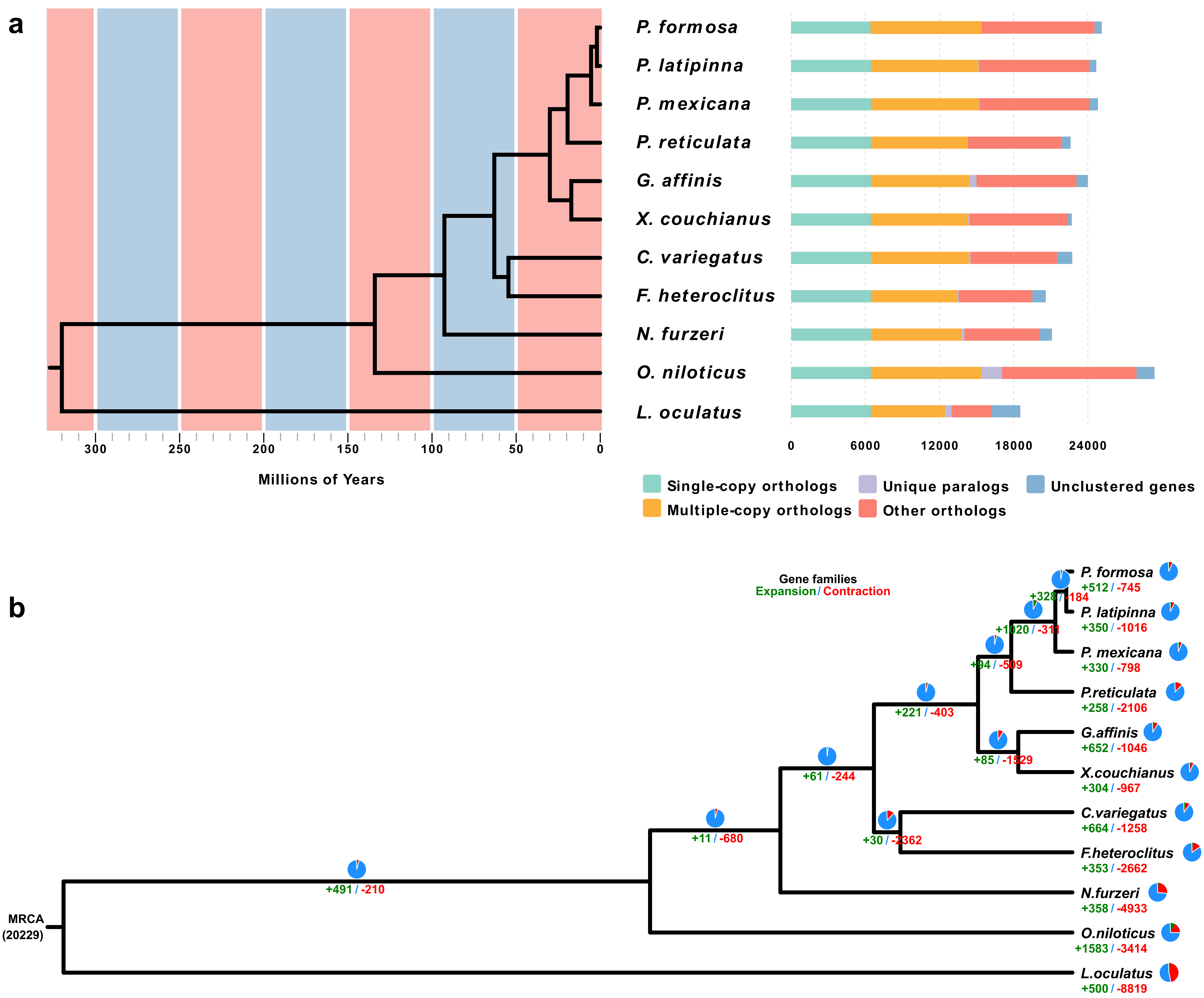

Figure 3

Click here to download Figure Figure_3.pdf

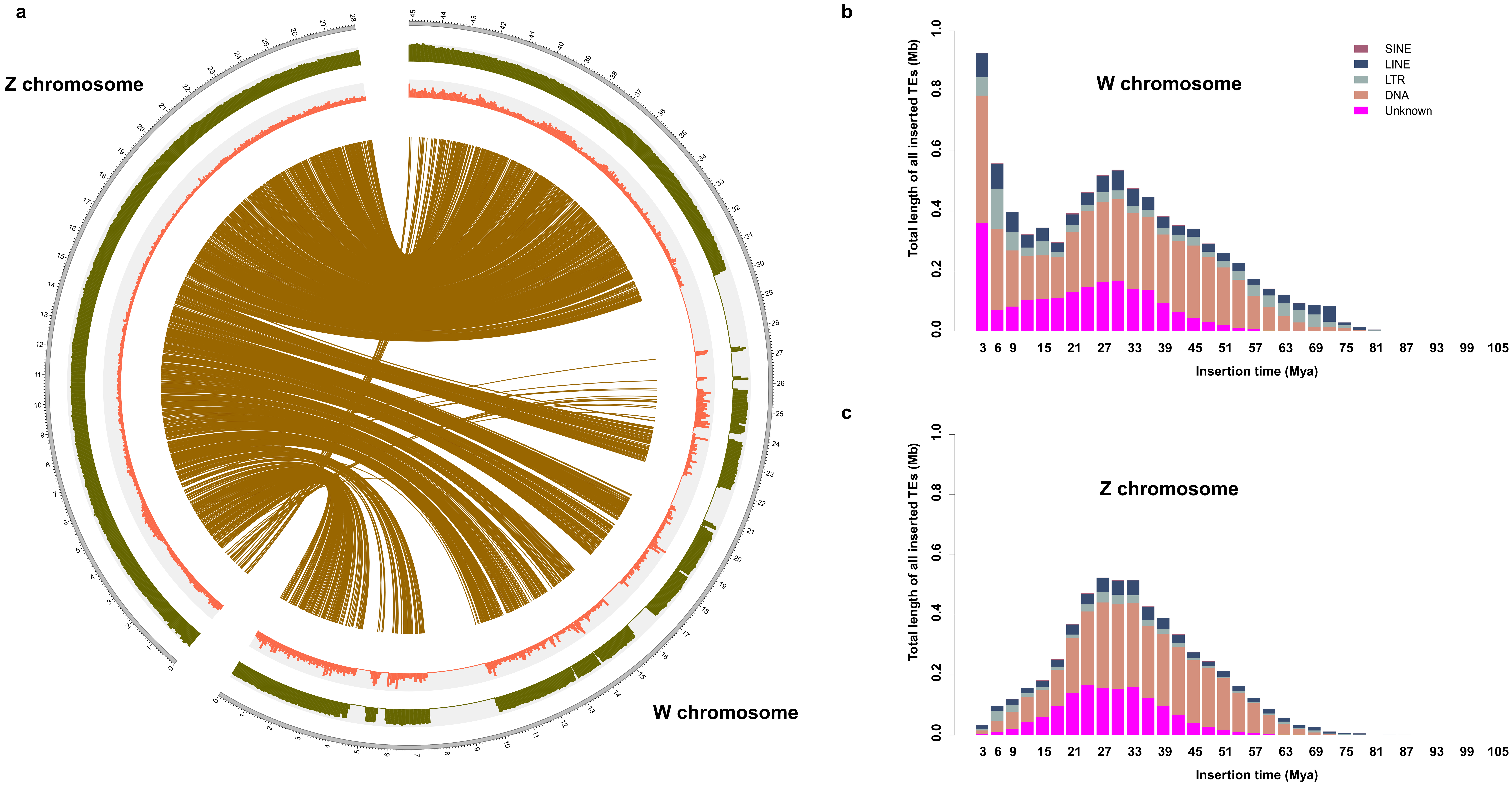

Click here to access/download
Supplementary Material
Supplemental_Information.docx

Click here to download Personal Cover Covering_Letter.doc

June 17th, 2020

Dear Dr. Hans Zauner,

I wish to re-submit our article, titled "**Chromosome-level genome assembly of the female western mosquitofish (*Gambusia affinis*)**" (previous manuscript ID: GIGA-D-20-00055) for consideration for publication in *GigaScience* as '***Data Note***'. All the data described in the manuscript have been uploaded to ftp://user39@parrot.genomics.cn. The paper was co-authored by Feng Shao, Arne Ludwig, Yang Mao, and Ni Liu.

We really appreciate you and reviewers very much for your positive and constructive comments and suggestions on our manuscript. The comments are all valuable and very helpful for revising and improving our manuscript, as well as the important guiding significance to our future studies. We have studied the comments carefully and have tried our best to revise our manuscript according to the comments. Please find the revised version, which we would like to re-submit for your kind consideration.

Thank you for your time. I look forward to hearing from you.

Sincerely,

Zuogang Peng, Ph.D. & Professor of Zoology

School of Life Sciences, Southwest University
No. 1 Tiansheng Road, Beibei, Chongqing 400715, China
Tel: +86-23-68253863; Fax: +86-23-68253005
E-mail: pzg@swu.edu.cn OR pengzuogang@gmail.com

Response to Reviewers and Editor

Reviewer #1:

This manuscript reports the first presentable genome assembly of the western mosquitofish Gambusia affinis. It lacks in-depth 'analysis' using the assembly product including, does not provide any sufficient advancement in genome science (e.g. to explain the unique phenotype of this fish), and thus does not warrant the publication, in the light of the enhanced standard of genome analysis maintained by the journal GigaScience. The product of the assembly should be evaluated by comparing with the records of the chromosome number by cytogenetic analysis and the flow cytometry-based nuclear DNA content. Apparently, N50 values provide a gauge for comparing with earlier assembly versions but do not guarantee the quality of the genome assembly.

Response: Thank you for your comments. According to the current research progress, we plan to publish this research in the form of 'Data Note' first, and conduct in-depth studies on functional genomics which actually are underway. And thanks for your suggestions, we had compared the results of the female *Gambusia affinis* genome assembly with the records of the chromosome numbers and the nuclear DNA content (i.e., Lamatsch et al., 2000; Tiersch, et al., 1989) to evaluate quality of the genome assembly.

Lamatsch D.K., Steinlein C., Schmid M., Schartl M. 2000. Noninvasive determination of genome size and ploidy level in fishes by flow cytometry: detection of triploid *Poecilia formosa*. Cytometry 39:91-95.

Tiersch T.R., Chandler R.W., Wachtel S.S., Elias S. 1989. Reference-standards for flow-cytometry and application in comparative studies of nuclear-DNA content. Cytometry 10:706-710.

Reviewer #2:

Shao, Peng and colleagues have sequenced and assembled the genome of a female Western mosquitofish /*Gambusia affinis*/. As mentioned by the authors in the Background section, this is actually the third assembly from this species, as the genome two male Western mosquitofishes have been sequenced and assembled earlier (Hoffberg et al., G3 8: 1855, 2018). According to the Abstract, the main motivation of this project was to promote the "organism as a suitable model to explore sex chromosome evolution and reproductive development in terms of the differentiation of ZW sex chromosomes, ovoviviparity, and the specialization of reproductive organs."

The authors managed to generate a high quality, chromosome/level assembly of the female Western mosquitofish genome. In the process, they have improved the metrics of the previously generated male genome and presumably added W-specific features to it, although they are not being described in detail.

This could be an interesting story that has a good chance to be read by all those, who are interested in the genomic analysis of mosquitofish reproduction, that of teleosts and even other vertebrates. Since these topics belong to some of the most fascinating areas of modern biology, they could justify special attention. Unfortunately, the presentation of the results does not entirely give justice to this expectation as the authors nearly entirely miss the opportunity to provide a detailed comparative analysis of the two sex chromosomes (see below in detail).

According to the knowledge of the referee, most claims made in the MS are novel. Some of the rest seem to be somewhat exaggerated and those would need to be toned down to provide better adherence to the data.

In general, the approaches that were taken by the authors are appropriate, although essential comparisons are missing from some of the bioinformatic analyses.

The claims are discussed in a brief, but informative manner.

In summary, this referee is on the opinion that this is a potentially valuable story that requires additional information and extensive revisions before it should be considered for publication.

Major criticisms:

* The lengthy title is potentially misleading for two reasons. First, a non-expert reader might believe that this is the first assembled genome of the species and not the third. Second, the although the species is indeed a 'potential model for sex chromosome differentiation and

reproductive development', but these aspects are not discussed in detail in the MS. The title must be revised and refocused. The referee's suggestion:
'Chromosome/level genome assembly of a female Western mosquitofish (Gambusia affinis)'.
Response: Thanks for your suggestions. We had changed the title to 'Chromosome-level genome assembly of the female western mosquitofish (*Gambusia affinis*)'.

* Although the authors claim that they "identified Z and W sex chromosomes", sequence level comparison of the two sex chromosomes of the species is completely missing from the MS. In the referee's opinion, this is an essential part of the story that must be added. Which parts of the chromosomes are similar enough to allow for meiotic pairing and recombination? How many pairs of groups of gene paralogs do they share? How does their repeat content compare? A detailed comparative map, preferably a syntenic one with as many genes as possible identified must be added to the revised MS.
Response: Thanks for your insightful comments. We had added comparative genomic analysis of Z and W chromosome to the manuscript. Please check the details in Figure 3 and *Recognition and evolution of sex chromosomes* part.

* As the authors have re-assembled the male genome and performed comparative analyses of that and their female genome, it is not obvious in the present version, which analyses were done on both genomes or just one of them. Throughout the MS, the genome used for the analyses must be clearly identified.
Response: Thanks. In the MS, we had stated the use of female and male *Gambusia affinis* genomes in different analyses.

* Data showing detailed comparative analysis of the W and Z chromosomes is missing. This must be shown in the revised version together with (i) a figure showing comparative syntenic analysis of the two sex chromosomes, and (ii) gene sets of the two sex chromosomes (all genes residing on one or both sex chromosomes must be also listed in a new Supplementary Table).
Response: Thanks. We had added this part. Please check the details in Figure 3 and *Recognition and evolution of sex chromosomes* part. And we also submitted the results to GigaDB.

* In the Results section that authors claim that "… the insertion time characteristics of the TEs on the W chromosome were specific because insertion time trends of autosomal TEs differed dramatically (Figure S4)". The referee disagrees with this statement, as most of the autosomal patterns shown on Supplementary Figure S4 (see. e.g. Chrs #02, #06, #18 and #21) also show a very pronounced peak during the 2-7 Mya time period. Taking this into consideration, the Z sex chromosome (not shown on S. Fig. S4) appears to show a very different pattern, when compared to W and the autosomes. Detailed explanation and perhaps a revised discussion of this whole phenomenon seems to be necessary.

Response: We are sorry for make the confusion. We had re-drawn this part in Figure 3 and Figure S5. The results show that the insertion time trends of autosomal and Z-chromosomal TEs are dramatically different from that of W chromosome.

* Figure legends must be able to 'stand on their own' allowing for clear interpretation of the essence of results shown even in the absence of the body text. Several legends in the current version do not comply with these requirements, as they miss essential info.

Response: Thanks. We had added more details for the figure legends.

* Supplementary tables and figures must adhere to the same set of criteria as those counterparts that have been placed into the MS proper. In the current version, several of them are messy with incomplete information. In the referee's opinion, this must be fully rectified.

Response: Thanks. We had rectified it.

* The MS contains a number of typos and grammatical errors, a few of which are noted below. A very thorough spellchecking through the use of a word processor is requested, and a correction by a native English speaker is highly recommended to bring the text to an acceptable level.

Response: Thanks. We have asked an English editing service from ‘Editage’ to make our manuscript clear and more readable.

Additional criticisms and suggestions (in the order of their appearance, not importance):

* Page 2, L25-26: As discussed above, it must be clearly spelled out in the first sentence that although this is the first female Western mosquitofish genome, but two other genomes exist from males of the same species.

Response: Thanks. We had changed it, and we had explained it in the Background part.

* P3, L48-51: This sentence does not read well and should be revised.

Response: Thanks. We had rephrased the sentence as “Although invasive western mosquitofish are harmful species with regard to the ecological environment, they are useful as model organisms in multiple life science studies, e.g., studies on behavior, ecology, toxicology, and population genetics.”

* P3, L58: The term /in vivo/ must be italicized here and throughout the MS.

Response: Thanks. We had changed it.

* P3, L63: Please revise the following definition to remove the redundancy: "intermediate transition state model for the study of the transition".

Response: Thanks. We had removed “intermediate transition state”.

* P4, L67-71: The whole paragraph needs clarification and revision.

Response: Thanks. We had rephrased the paragraph as “Male *G. affinis* (ZZ type) scaffold level genome data have been published, and they serve as resources for comparative genomics among poecilids and teleosts. However, recently released data are not sufficient to explore the evolution of ZW sex chromosomes and the reproduction mode of female *G. affinis*. Further studies are needed to overcome the lack of high-quality genomic data for female *G. affinis* (ZW type).”

* P4, L74-77: The last sentence of the paragraph is too general and as such, it overclaims the data. The current genome assembly - together with the previous ZZ ones - indeed provide valuable resources for the analysis of sex determination mechanisms in Western mosquitofish and its relatives, but for the analysis of other aspects, the previous assembly was already available.

Response: Thanks. Now we added the comparative genomic analysis of the Z and W chromosomes and emphasized its importance.

* P45, L81: Was the individual used for sequencing a mature adult? How was the sex of the fish verified? Are there signs of obvious sexual dimorphism in Western mosquitofish?

Response: Thanks. Yes, mature adult had used for sequencing, and western mosquitofish has obvious sexual dimorphism.

* P4, L87: The ID of the permit must be provided here.

Response: Thanks. We had added it.

* P5, L110: These are organs and not just tissues. Were the whole organs processed?

Response: We are sorry for making the confusion. They are tissues and we had not processed the whole organs. We had changed this part in MS.

* P9, L198-202: In the referee's opinion, it is not acceptable to base one of the most important set of calculations in the whole MS on a formula that contains a value (nucleic acid substitution rate from /X. maculatus/) from an unpublished study. Those calculations and data must be described here - or in the Supplementary Data file - in detail.

Response: Thanks. We had re-written this part in MS.

* P11, L262: In fact, this chapter must be called Results and Discussion, as the latter is missing from the end.

Response: Thanks. We had changed it as you suggested.

* P12, L280-281 & 284-285: The species has 24 pairs of chromosomes. If the Z and W sex chromosomes are distinguishable, as the authors claim, there should 25 chromosomes here, not just 24.

Response: Thanks. In female mosquitofish genome, *G. affinis* have 24 pairs of chromosomes, 23 pairs of autosomes and 1 pair of sex chromosomes, we only assembled the W chromosome, and the Z chromosome was obtained by comparing with the genomic data of the male mosquitofish.

* P12, L287: Please correct "female" to 'male'.

Response: Thanks. We had changed it.

* P13, L292: Were both genomes (i.e. ZW and ZZ) used to predict repeat sequences? If only the former, then please correct "genomes" to 'ZW genome' or 'female genome'.

Response: Thanks. We had changed it to "Assembled chromosome-level genome of female *G. affinis* were used to predict repeat sequences."

* P13, L293: Please correct to 'sequences'.

Response: Thanks. We had changed it.

* P13, L298-299: Please correct to : "transposons … were".

Response: Thanks. We had changed it.

* P13, L310-311: The complete list of annotated genes must be made available for the readers.

Response: Thanks. We had submitted it to GigaDB.

* P13, L312: All these must be provided in the form of Supplementary Excel tables or links to a database where they can be downloaded from.

Response: Thanks. We had submitted it to GigaDB.

* P14, L322: The closest species to /G. affinis/ on the phylogenetic tree is actually /X. couchianus/ and not /X. maculatus/ as indicated in the text.

Response: Thanks. We had changed "*X. maculatus*" to "*X. couchianus*".

* P14, L323: To the referee Figure 2A and Figure S3 appear to be identical.

Response: Thanks. Figure S3 shows the specific differentiation time of different nodes.

* P15, L343-348: This whole paragraph must be moved to Background. The paragraph must be revised and the grammatical error eliminated from the second and third sentences. Moreover, the third sentence must be revised and toned down, as a single model will not be enough to understand the evolution of sex chromosomes in >34k species of fishes.

Response: Thanks. We had changed this part as "Results and Discussion", and we re-wrote it, so we had not moved it to Background. We had revised sentences and changed "very important for" to "conductive to".

* P15, L349: Please correct to: '…shows heterogamy of ZW type…'.

Response: Thanks. We had corrected it.

* P15, L355-357 and Figure 3A: This data must be shown in more detail, preferably accompanied with a figure. After all, the primary motivation of the project was to learn more about the differences between the sex chromosomes of the species.

Response: Thanks for your insightful suggestions. We had added the comparative genomic analysis of Z and W chromosomes.

* Ibid: In order to be able to make this claim, LG01 of the female genome must be broken into the two sex chromosomes.

Response: We are sorry for making the confusion. From the assembly results of female mosquitofish, it seems that we only assembled W chromosome, so we constructed genomic data of male mosquitofish with chromosome level, and obtained Z chromosome through alignment.

* P15, L358: Wrong statement: TEs are not related to the evolution of sex chromosomes. The referee proposes the following: 'TEs were shown to be play an important role in the evolution of sex chromosomes…'.

Response: Thanks. We have changed to the sentence as you suggested.

* P15, L359: The second part of the sentence would benefit from more detailed explanation.

Response: Thanks. We rephrased the sentences as “Some researchers have studied the role of transposons in sex chromosome differentiation, and they found that TEs are closely related to the evolution of sex chromosomes, with their accumulation and loss having huge effects on the lengths of sex chromosomes.”

* P15, L360: Please correct to: '…have studied the role of transposons in sex chromosome differentiation.'

Response: Thanks. We had corrected it.

* P15, L361 - P16, L364: This sentence needs to be fully revised. Calling individuals containing both types of sex chromosomes as 'heterozygotes' is potentially misleading. The term 'longer length' should be replaced with 'increased length' or something similar.

Response: Thanks. We had corrected “heterozygotes” to “Z and W chromosome”. Because we want to describe the uncertainty of the difference in the length of the Z chromosome and the W chromosome, so we used ‘longer length’ instead of ‘increased length’.

* P16, L371-373: The authors propose that the process of sex chromosome differentiation "may be enriched on the W chromosome by TE, leading to an increase in sex chromosome size". Indeed, increased TE content has been observed on mammalian and avian sex chromosomes in comparison to autosomes by several studies. The referee has tried to compare the total TE content of the W chromosome and those of the autosomes based on Supplementary Figure S4. Unfortunately, this turned out to be a rather difficult task as the labels on the Y axes on the S4 panels were very hard to read. Based on that rough comparison, there does not seem to be a substantial increase in the total TE content of W in comparison of most autosomes. The authors are requested to provide these data (i.e. complete length of TEs for every chromosome) in the form of a table, and if the differences are not significant, they must also explain the lack of difference.

Response: We are sorry for making the confusion. Actually, we're talking about the differentiation of Z and W chromosomes, the comparison of sex chromosome and autosomal transposon contents cannot solve this problem (Nevertheless, we show the TE lengths of different chromosomal below). This also have been explained in the last paragraph of the Results and Discussion section (The composition of the gap). We re-drew the time distribution diagram of TEs insertion and unified the ordinate length, so that we could clearly see the specificity of W chromosome.

Chromosome NO.	TE lengths (bp)
LG01 (W)	8,452,544
LG02	6,999,148
LG03	6,975,421
LG04	7,054,596
LG05	7,838,824
LG06	6,645,040
LG07	6,633,856
LG08	5,121,971
LG09	6,310,686
LG10	5,627,849
LG11	5,832,363
LG12	7,242,956
LG13	5,988,505
LG14	6,127,517
LG15	4,934,044
LG16	5,202,068
LG17	3,882,318
LG18	7,473,457
LG19	5,846,778

LG20	6,630,312
LG21	5,196,087
LG22	6,024,825
LG23	6,328,940
LG24	2,580,328

* P16, L374: The referee suggests to replace the word 'accurate' with 'correct'.

Response: Thanks. We had corrected it.

* P16, L375-376: What exactly do the authors mean by "in the near future (later than 2-7 Mya)". This should be 'in the recent past (between 2-7 Mya)'.

Response: We are sorry for making the confusion. We had changed “near future” to “recent past”.

* P16, L376-377: The referee thinks that authors statement on "the presence of a very recent TE concentrated insertion event on the W chromosome" must be revised as the word 'concentrated' might mislead the readers to think, that these insertions happened in particular regions of the W chromosome.

Response: Thanks. We had corrected “the presence of a very recent TE concentrated insertion event on the W chromosome” to “the presence of a very recent mass insertion events of TEs on the W chromosome”.

* P16, L380: The authors mention the presence of "long gaps on the W chromosome". This must be visualized in the form of a Figure that provides comparative analysis of the assemblies of the two sex chromosomes, as mentioned above.

Response: Thanks. We had added this part to Figure3.

* P16, L380-385: This paragraph is vague and as such, it needs to be extensively revised.

Response: Thanks. We had revised this paragraph.

* P17, L388-397: The whole conclusion must be fully revised to improve the flow, eliminate redundancies and circular arguments. The last sentence must be toned down, as a genome of slightly lower quality has been available since 2018.

Response: Thanks. We had revised this paragraph. We had corrected last sentence to “In conclusion, the high-quality genomic data generated in this study lay the foundation for the

study of chromosome evolution, reproductive characteristics, and sexual dimorphism in western mosquitofish.”

* Figure 1: This figure is of confirmatory value only. Therefore, the referee proposes that it should be moved to the Supplement. LG01 of the female genome must be divided into the two sex chromosomes. Labels (i.e. female or male) should be inserted above the two panels. Labels must be moved to the upper left corner of each panel.
Response: Thanks. We had re-drawn it and moved to the Supplement.

* Figure 2: This figure contains three panels, not just two. All three must be labeled and explained in detail in the legend. There are several labels and signs here that would require explanation in the legend (e.g. MRCA, the circle diagrams, the numbers below, etc.). What is the point of having 'million years ago' estimates on the X axis to four (!!) decimals?
Response: Thanks. Actually, in Figure 2A, the phylogenetic tree and the bar chart are connected. We had added explanations in the legends. We had deleted X axis from Figure 2B.

* Figure 3A: On the chord diagram, the two halves must be clearly indicated with ZW or ZZ signs next to them. ZW01 must be divided into the two sex chromosomes: W and Z.
Response: Thanks. We had re-drawn it.

* Figure 3B-C: What exactly is shown on the Y axis? Is it the combined length of all transposons estimated to have been inserted during that time period? This should be properly explained in the legend. If yes, how does this model deal with an element that jumps in and out repeatedly?
In addition to the pattern of two sex chromosomes, either that of a typical autosome or the average pattern of all autosomes must also be shown.
The bar graphs should have the same dimensions on the Y axis (1.0). The X axis should not extend beyond 105 Mya (and not My), as there are no visible signs of insertion there. One set of shared labels would be enough, but it needs to be enlarged. The label (Z Chromosome or W chromosome) should be added above each the two bar graphs.
Response: Thanks. We had re-drawn it. In the study of TEs, the concept of jump out is very difficult to define. Since the CLASS I transposons implement “copy-to-paste” with RNA as the medium, there is no "jump"; although CLASS II is a cut-to-paste mode, the cut part will

be restored by homologous repair, so there is no "jump out" in essence. The model used in this study can only estimate the insertion time based on the state of the existing transposons in the genome.

* Table S1: The two sex chromosomes should be differentiated and labeled here too.

Response: Thanks. We had changed it.

* Table S3: The header must be revised to eliminate the grammatical error. The referee proposes: 'Comparative analysis of the annotated gene set of Western mosquitofish with those of six teleosts'. The species must be listed in alphabetic order. Also, there is no point to provide sequence lengths to two decimal precision, especially when they are several hundred or thousand base pairs long.

Response: Thanks. We had changed it.

* Table S6: Explanations for GO_Class abbreviations must be added to the bottom of the table as a footnote,

Response: Thanks. We had added it.

* Supp. Figure S1: Please correct the type in the Y-axis label.

Response: We are sorry for making the confusion. This kinds of graph is widely used in genome survey (Kataoka, et al. 2020; Stevens, et al. 2020; Tan, et al. 2020; Wang, et al. 2020). We had changed the legend as fellow: “Figure S1Frequency distribution of the 17-mer graph analysis used to estimate the size of female *Gambusia affinis*.”

Kataoka K, Minei R, Ide K, Ogura A, Takeyama H, Takeda M, Suzuki T, Yura K, Asahi T. 2020. The Draft Genome Dataset of the Asian Cricket *Teleogryllus occipitalis* for Molecular Research Toward Entomophagy. Front Genet 11:470.

Stevens L, Rooke S, Falzon LC, Machuka EM, Momanyi K, Murungi MK, Njoroge SM, Odinga CO, Ogendo A, Ogola J, et al. 2020. The Genome of *Caenorhabditis bovis*. Current Biology 30:1023-1031 e1024.

Tan MH, Gan HM, Lee YP, Grandjean F, Croft LJ, Austin CM. 2020. A Giant Genome for a Giant Crayfish (*Cherax quadricarinatus*) With Insights Into cox1 Pseudogenes in Decapod Genomes. Front Genet 11:201.

Wang P, Luo Y, Huang J, Gao S, Zhu G, Dang Z, Gai J, Yang M, Zhu M, Zhang H, et al. 2020. The genome evolution and domestication of tropical fruit mango. Genome Biology 21:60.

* Supp. Figure S2: Please enlarge the panels and label each with a letter. Please enlarge the labels on the two axes. There is no need to insert the same color code into each panel, one would be enough. Please make sure that labels above the panels and those in the legend fully agree (the distributions are compared across the species). Please correct the typo 'intro' in the legend.

Response: Thanks. This image is a mosaic of six individual images and has enough resolution to zoom in. If one color code used in this figure, additional panel is required, 7 panels are not pretty. We had changed “intro” to “intron”.

* Supp. Figure S3: This table should be moved to replace Figure 2 Panel A.

Response: Thanks. Figure S3 shows the specific differentiation time of different nodes (more intuitive data of species differentiation time), it’s very important.

* Supp. Figure S4: This Figure is useless in its current form as the labels cannot be deciphered. Please change the format from a 4X6 to a 3X8+1 (as both sex chromosomes must be shown, not just the W). Please enlarge the panels themselves, plus the numbers and labels on the axes to make sure that they are readable. One set of explanation would be enough, but it must be enlarged. Much more detailed explanation must be provided in the legend.

One more issue related to this figure: All chromosomes, including the sex chromosomes, seem to have a peak of transposons inserted between 20-40 Mya. Is this a specific feature of the Western mosquitofish genome or is that true for other teleost genomes as well? Either way, an explanation would be required here.

Response: Thanks. We had re-drawn it. Z and W chromosomes were shown in MS, autosomes were shown in Supplemental Information.

Thanks for your insightful comments. Actually, in another piece of work that hasn't been published yet, we calculated transposon insertion times for about 80 vertebrate species, different species or taxa have different peaks, and it may be related to some evolutionary process. This is another part of our work, so we won't go into details here.

==

Additional editorial advice:

===

General comments.

This looks like a nice genome assembly and it will be a good resource for comparative studies. It would have been good to have had more comparison between the male and female genomes. At times, they are treated as a single genome. Authors need to make clear which one is used at all occasions. It would be good to see a comparison of the putative Z and W chromosomes apart from the TE numbers. For example, the number of coding genes, the distribution of TE along the chromosome, etc.

Authors need to have more comparative analysis with X. couchianus. Specifically, are there translocations that distinguish the two karyotypes? Or, does every chromosome in mosquitofish correspond to a single chromosome in Xiphophorus? If it does, then it is essential that the chromosome numbering system of both species are the same. The mosquitofish ortholog of Chromosome 1 that has already been established for Xiphophorus should be called mosquitofish chromosome 1. Etc. This is essential for comparative studies. And even if there are one or two translocations that distinguish the two species, the chromosomes that do correspond must have the same numbers.

Especially, it is important to tell the reader which Xiphophorus chromosome corresponds to the sex chromosome of mosquitofish.

The English writing in this Ms. is more understandable than most Mss. that come from China.

Specific comments:

20 worldwide biological invasion and IS an important research model

Response: Thanks. We had corrected it.

110 Six RNA tissues (brain, liver, heart, gills, gonads, and muscles) were extracted

For RNA analyses, six tissues (brain, liver, heart, gills, gonads, and muscles) were extracted

Response: Thanks. We had corrected it.

127 SMRTbell Template Prep Kit (Pacific Biosciences). At last, library for SMRT cell was

127 SMRTbell Template Prep Kit (Pacific Biosciences).FINALLY, A library for SMRT cell was

Response: Thanks. We had corrected it.

68 [12], high-quality genomic data are the basis for addressing these topics.

Hoffberg et al (reference #12) has a Chicago assembly with 25 scaffolds, just one more than the number of chromosomes. Is there a reason to think it's missassembled? Was the new assembly compared to that assembly? This should be discussed. Would combining the two assemblies make an even better final assembly?

Response: Thanks. We had toned down this part. Actually, the Chicago assembly result have more than 2000 scaffolds, and without chromosome-level results.

267 and the estimated heterozygosity rate was approximately 0.42%.

Need to tell us how many different individuals went into each phase of the sequencing project and how many haploid genomes this heterozygosity rate is based on.

Response: Thanks. We had used a general method (jellyfish with *17-mer* frequency distribution) calculated heterozygosity rate by one individual, this method is widely used (Kataoka, et al. 2020; Stevens, et al. 2020; Tan, et al. 2020; Wang, et al. 2020).

Kataoka K, Minei R, Ide K, Ogura A, Takeyama H, Takeda M, Suzuki T, Yura K, Asahi T. 2020. The Draft Genome Dataset of the Asian Cricket *Teleogryllus occipitalis* for Molecular Research Toward Entomophagy. Front Genet 11:470.

Stevens L, Rooke S, Falzon LC, Machuka EM, Momanyi K, Murungi MK, Njoroge SM, Odinga CO, Ogendo A, Ogola J, et al. 2020. The Genome of *Caenorhabditis bovis*. Current Biology 30:1023-1031 e1024.

Tan MH, Gan HM, Lee YP, Grandjean F, Croft LJ, Austin CM. 2020. A Giant Genome for a Giant Crayfish (*Cherax quadricarinatus*) With Insights Into cox1 Pseudogenes in Decapod Genomes. Front Genet 11:201.

Wang P, Luo Y, Huang J, Gao S, Zhu G, Dang Z, Gai J, Yang M, Zhu M, Zhang H, et al. 2020. The genome evolution and domestication of tropical fruit mango. Genome Biology 21:60.

275 we used BUSCO [17] to assess the completeness of the assembly genome

275 we used BUSCO [17] to assess the completeness of the assemblED genome

Response: Thanks. We had corrected it.

287 approximately 679.4 Mb (Table 1). In the female G. affinis genome, 734 scaffolds were

Shouldn't it be the male? figure 1B is supposed to be the male genome assembly. Again, the original paper (ref #12) has 26 scaffolds, what's the difference to this new one? Was this new male assembly done with the raw data?

Response: Thanks. We had toned down this part. Actually, the Chicago assembly result have more than 2000 scaffolds, and without chromosome-level results.

302 For genome annotation, 23,997 protein-coding genes were predicted

The male G. affinis genome in reference #12 has 21,144 predicted genes. What is the number in the same but newly assembled male genome? What is the gene number differenfe between the male and female genomes? Is the difference due to the use of different annotation software? How about using the software in the current paper in the assembly from reference #12? Are different genes predicted? Would joining the two predictions result in a better total gene prediction?

Response: Thanks. In this study, we used PromethION sequencer to achieve long reads data, the assembly results of long reads were more complete than those of the Chicago data assembly, so we think that's the main reason for the difference in gene annotation results. Since gene annotation is very resource-intensive and time consuming, we did not conduct a comprehensive review of the published results. In the later work on functional genomics, we will conduct a careful review and functional verification of the genes we are interested.

310 reliable (Table S4). Finally, 23,737 genes were annotated in at least one of the databases

Unclear what databases are implied.

Response: Thanks. We had rephrased the sentence as following: "Finally, 23,737 genes were annotated in at least one of the databases (KOG, KEGG, NR, SwissProt, GO)".

320 of the Poeciliidae family, G. affinis had a closer relationship with X. maculatus.

How does that compare with previously published phylogenies?

Response: Thanks. We had rephrased the sentence as following: "of the Poeciliidae family, *G. affinis* had a closer relationship with *X. couchianus*, consistent with previously published phylogenies".

327 family expansion and contraction analysis

Are there differences between the male and female genomes that are not in the sex chromosomes? and are there differences in the sex chromosomes? Need to be clear here and many other places which genome authors are talking about.

Response: We are sorry for making the confusion. We had rephrased the sentence as following: "In order to examine the evolutionary history of gene families, we performed gene family expansion and contraction analysis with the female *G. affinis* genome". We had corrected it here and also other place.

334 selected genes in the G. affinis genome

Are those genes scattered across the genome or are there specific genomic regions that might be under selection?

Response: Thanks. They scattered across the genome.

343 Sex determination system in fish is variable

343 Sex determination systemS in fish ARE variable

Response: Thanks. We had corrected it.

343 Sex determination system in fish is variable

Also mention environmental mechanisms and citations.

Response: Thanks. Here, we mentioned sex determination systems rather than sex determination factors.

344 generally do not have highly differentiated sex chromosomes,

Do authors mean that few fish have sex chromosomes of any type? Or that among the fish that have sex chromosomes, few species have morphologically differentiated sex chromosomes? Needs rewriting.

Response: We had rephrased the sentence as following: “Fish generally do not have highly morphologically differentiated sex chromosomes”.

355 Analysis of the synteny of the whole genomes of female and male mosquitoes by

Mosquitoes? The fly?

Response: We are sorry for making the confusion. We had changed “mosquitoes” to “mosquitofish”.

369 and ZW sex determination mechanisms had independent origins in G. holbrooki and G.
370 affinis [43].

Need to be more explicit. Tell us explicitly which one has a ZW and which an XY system along with citation.

Response: We had rephrased the sentence as following: “XY and ZW sex determination mechanisms had independent origins in *G. holbrooki* and *G. affinis*, respectively”.

375 observe a large number of transposon insertions in the W chromosome in the near future

In the near future? Like in a million years from now? Or in the recent past, like a million years ago.

Response: We are sorry for making the confusion. We had changed “near future” to “recent past”.

377 concentrated insertion event on the W chromosome (Figure 3C),

We need a specific comparison of W and Z chromosomes.

Response: Thanks. We added the comparative genomic analysis of the Z and W chromosomes.

380 Moreover, we speculate that most of the long gaps on the W chromosome

What about gaps on the Z chromosome?

Response: Thanks. We had showed it in Figure 2.

384 Accordingly, our results showed that the cause of sex chromosome differentiation in G.

Most people think that sex chromosome differentiation is due to the lack of recombination between the two sex chromosomes. TE insertions would be quite secondary, not the cause. The TEs just make the W longer than the Z. Need to distinguish causes from responses, which are confused in the current version.

Response: Thanks. We can't agree with you more. Therefore, we specify this conclusion in the mosquitofish.

392 transport in mosquitofish deserves attention. We conducted a preliminary study on the
393 sex chromosome differentiation in specific fishes based

First, the authors didn't really compare W to Z, they just talk about the W except for length. Second, they talk about a specific fish species, not many species as the sentence suggests.

Response: Thanks. We added the comparative genomic analysis of the Z and W chromosomes. We had rephrased the sentence as following:" We conducted a preliminary study on the sex chromosome differentiation based on the specificity of the sex chromosome in female western mosquitofish".

394 chromosome in female western mosquitofish, providing data support for the previous
395 hypothesis.

Be specific. What was the previous hypothesis? What is the new hypothesis that replaces it? This is unclear.

Response: We are sorry for making the confusion. We had rephrased the sentence as following: "and provided data to support the previous hypothesis that a longer W chromosome is associated with the activity (insertion) of TEs".

402 NCBI Assembly with the accession number of JAAAQJ000000000

Be sure to update with the real number. Is the access number only for the female genome or does it also include the reassembled male genome from reference #12. Both the male and the female genomes need to be submitted for the paper to be accepted.

Response: Thanks. JAAAQJ000000000 is a real number, it just hasn't been released yet. We had submitted the male and the female genomes to GigaDB.

601 Figure 1. Western mosquitofish genome scaffold contact matrix using Hi-C data. (A) Write within the figure the sexes after the A and the B designators.

Response: Thanks. We had corrected it.
