# Supplementary material for: Chromosome-level genome assembly of the female western mosquitofish (Gambusia affinis)
Source: Gigascience. 2020 Aug 27;9(8):giaa092. doi: 10.1093/gigascience/giaa092 (PMC7450667; doi:10.1093/gigascience/giaa092)
Supplement: giaa092_GIGA-D-20-00179_Revision_2 [file giaa092_giga-d-20-00179_revision_2.pdf]

GigaScience

Chromosome-level genome assembly of the female western mosquitofish (Gambusia affinis)

--Manuscript Draft--

Manuscript Number:	GIGA-D-20-00179R2
Full Title:	Chromosome-level genome assembly of the female western mosquitofish (Gambusia affinis)
Article Type:	Data Note
Funding Information:	National Key Research and Development Program of China (2018YFD0900805) — Prof. Zuogang Peng<br>National Natural Science Foundation of China (31872204) — Prof. Zuogang Peng<br>Fundamental Research Funds for the Central Universities (XDJK2020C043) — Dr. Feng Shao<br>Natural Science Foundation of Chongqing Postdoctoral Science Foundation (cstc2019jcyj-bshX0071) — Dr. Feng Shao

Abstract:

Background

The western mosquitofish (Gambusia affinis) is a sexually dimorphic poeciliid fish known for its worldwide biological invasion and therefore an important research model for studying invasion biology. This organism may also be used as a suitable model to explore sex chromosome evolution and reproductive development in terms of differentiation of ZW sex chromosomes, ovoviviparity, and specialization of reproductive organs. However, there is a lack of high-quality genomic data for the female G. affinis ; hence, this study aimed to generate a chromosome-level genome assembly for it .

Results

The chromosome-level genome assembly was constructed using Oxford nanopore sequencing, BioNano, and Hi-C technology. G. affinis genomic DNA sequences containing 217 contigs with an N50 length of 12.9 Mb and 125 scaffolds with an N50 length of 26.5 Mb was obtained by Oxford nanopore and BioNano, respectively, and the 113 scaffolds (90.4% of scaffolds containing 97.9% nucleotide bases) were assembled into 24 chromosomes (pseudo-chromosomes) by Hi-C. The Z and W chromosomes of G. affinis were identified by comparative genomic analysis of female and male G. affinis , and the mechanism of differentiation of the Z and W chromosomes was explored. Combined with transcriptome data from six tissues, a total of 23,997 protein-coding genes were predicted and 23,737 (98.9%) genes were functionally annotated.

Conclusions

The high-quality female G. affinis reference genome provides a valuable omics resource for future studies of comparative genomics and functional genomics to explore the evolution of Z and W chromosomes and the reproductive developmental biology of G. affinis .

Corresponding Author:	Zuogang Peng, Ph.D.<br>Southwest University<br>Chongqing, CHINA
Corresponding Author Secondary Information:	
Corresponding Author's Institution:	Southwest University
Corresponding Author's Secondary Institution:	
First Author:	Feng Shao
First Author Secondary Information:	

Powered by Editorial Manager® and ProduXion Manager® from Aries Systems Corporation

**Order of Authors:**	Feng Shao
	Arne Ludwig
	Yang Mao
	Ni Liu
	Zuogang Peng
**Order of Authors Secondary Information:**	
**Response to Reviewers:**	1) Please include ORCIDs for all authors on the title page, if available.<br>Response: Thanks. We had added it on the title page.<br><br>2) In line 186, instead of the URL (https://www.ncbi.nlm.nih.gov/assembly/GCA_003097735.1) please cite just the permanent NCBI accession number (URLs are not stable and may change over time)<br>Response: Thanks. We had changed it to 'GCA_003097735.1'.<br><br>3) line 200 ff, Danio rerio and other fish genomes: Please move citations of third-party data sources (e.g. (ftp://ftp.ensembl.org/pub/release-96/fasta/danio_rerio/dna/) to the bibliography, and cite them by number in the text. Please refer to our citation guidelines for the format.<br>https://academic.oup.com/gigascience/pages/instructions_to_authors#Preparing%20Main%20Manuscript%20Text<br>(example format to cite an FTP site: [xx] Doe, J: Trivial HTTP, RFC2169. ftp://ftp.isi.edu/in-notes/rfc2169.txt (1999). Accessed 12 Nov 1999.)<br>If available, please also mention BioProject IDs or other permanent (NCBI) accession numbers for these data sources.<br>Response: Thanks. We had rephrased the sentence as 'Danio rerio (GCA_000002035.4), Oryzias latipes (GCA_002234715.1), Nothobranchius furzeri (GCA_001465895.2), Xiphophorus maculatus (GCA_002775205.2), and Poecilia formosa (GCA_000485575.1) were used to predict G. affinis genes by GeMoMa.'<br><br>4) citation of software tools used: For all software tools you mention, rather than mentioning the URL in the main text, please cite the relevant published literature (or the project home page, if no publication is available that describes the tool) from the bibliography. Also if you mention other URLs in the main text, please move them to the bibliography and treat them as a citation.<br>Response: Thanks. We had changed all software tools URL to citations.<br><br>5) Please also add RRIDs (Research Resource Identifiers) for the software tools you used, if available. You can find RRIDs here: https://dknet.org/data/source/nlx_144509-1/search. For example the RRID for Augustus is (RRID:SCR_008417).<br>Response: Thanks. We had added RRIDs for the software tools we used.<br><br>6) Please also include the fishbase ID for G. affinis.<br>Response: Thanks. We had added 'Fishbase ID: 3215' in MS.<br><br>7) If you have one, please also include an image of a female (or maybe female and male, for comparison) G. affinis specimen as Figure 1.<br>Response: Thanks. We had added image of G. affinis as Figure 1.
**Additional Information:**	
**Question**	**Response**
Are you submitting this manuscript to a special series or article collection?	No
**Experimental design and statistics**<br><br>Full details of the experimental design and statistical methods used should be given	Yes

Powered by Editorial Manager® and ProduXion Manager® from Aries Systems Corporation

in the Methods section, as detailed in our Minimum Standards Reporting Checklist. Information essential to interpreting the data presented should be made available in the figure legends.<br><br>Have you included all the information requested in your manuscript?	
**Resources**<br><br>A description of all resources used, including antibodies, cell lines, animals and software tools, with enough information to allow them to be uniquely identified, should be included in the Methods section. Authors are strongly encouraged to cite Research Resource Identifiers (RRIDs) for antibodies, model organisms and tools, where possible.<br><br>Have you included the information requested as detailed in our Minimum Standards Reporting Checklist?	Yes
**Availability of data and materials**<br><br>All datasets and code on which the conclusions of the paper rely must be either included in your submission or deposited in publicly available repositories (where available and ethically appropriate), referencing such data using a unique identifier in the references and in the “Availability of Data and Materials” section of your manuscript.<br><br>Have you have met the above requirement as detailed in our Minimum Standards Reporting Checklist?	Yes

Manuscript Click here to access/download;Manuscript;GigaScience.docx

Chromosome-level genome assembly of the female western mosquitofish (*Gambusia affinis*)

Feng Shao[1], Arne Ludwig[2,3], Yang Mao[1], Ni Liu[1], Zuogang Peng[1, *]

[1] Key Laboratory of Freshwater Fish Reproduction and Development (Ministry of Education), Southwest University School of Life Sciences, Chongqing 400715, China

[2] Department of Evolutionary Genetics, Leibniz-Institute for Zoo and Wildlife Research, 10315 Berlin, Germany

[3] Albrecht Daniel Thaer-Institute, Faculty of Life Sciences, Humboldt University Berlin, 10115 Berlin, Germany

Running title: Genome assembly in *G. affinis*

*Correspondence: pzg@swu.edu.cn

ORCID:

Feng Shao https://orcid.org/0000-0002-4648-2493

Arne Ludwig https://orcid.org/0000-0001-7249-9953

Zuogang Peng https://orcid.org/0000-0001-8810-2025

Abstract

Background

The western mosquitofish (*Gambusia affinis*) is a sexually dimorphic poeciliid fish known for its worldwide biological invasion and therefore an important research model for studying invasion biology. This organism may also be used as a suitable model to explore sex chromosome evolution and reproductive development in terms of differentiation of ZW sex chromosomes, ovoviviparity, and specialization of reproductive organs. However, there is a lack of high-quality genomic data for the female *G. affinis*; hence, this study aimed to generate a chromosome-level genome assembly for it.

Results

The chromosome-level genome assembly was constructed using Oxford nanopore sequencing, BioNano, and Hi-C technology. *G. affinis* genomic DNA sequences containing 217 contigs with an N50 length of 12.9 Mb and 125 scaffolds with an N50 length of 26.5 Mb was obtained by Oxford nanopore and BioNano, respectively, and the 113 scaffolds (90.4% of scaffolds containing 97.9% nucleotide bases) were assembled into 24 chromosomes (pseudo-chromosomes) by Hi-C. The Z and W chromosomes of *G. affinis* were identified by comparative genomic analysis of female and male *G. affinis*, and the mechanism of differentiation of the Z and W chromosomes was explored. Combined with transcriptome data from six tissues, a total of 23,997 protein-coding genes were predicted and 23,737 (98.9%) genes were functionally annotated.

Conclusions

The high-quality female *G. affinis* reference genome provides a valuable omics resource for future studies of comparative genomics and functional genomics to explore the evolution of Z and W chromosomes and the reproductive developmental biology of *G. affinis*.

Keywords: *Gambusia affinis*, Nanopore sequencing, Hi-C, genome assembly, sex chromosome differentiation

Background

The western mosquitofish (*Gambusia affinis*) is a well-known invasive species of the Poeciliidae family, native to North America. To date, *G. affinis* has invaded many countries worldwide, competing successfully with native fish everywhere and destroying the ecological balance, leading to recognition by the World Conservation Union as one of the world's top 100 invasive alien species.

Although invasive western mosquitofish are harmful species with regard to the ecological environment, they are useful as model organisms in multiple life science studies, e.g., studies on behavior [1, 2], ecology [3, 4], toxicology [5, 6], and population genetics [7-9]. In addition, western mosquitofish have many interesting biological features. For example, the ZZ/ZW sex determination system in female *G. affinis* contains a W chromosome that is much longer than the Z chromosome [10], which is in contrast to the ZW chromosomes found in many birds and reptiles [11]. Additionally, in terms of reproductive development, female *G. affinis* is an oviparous fish, fertilized in the body, and developed in the body; however, without a placenta, the nutrients for the development of the fertilized egg come from yolk, and not from maternal supply. Male *G. affinis* horn fins are specialized gonopodium for *in vivo* fertilization. These biological characteristics are generally of interest for biologists, as they can provide insights into the evolution of vertebrate sex chromosomes, such as the mechanism of Z and W sex chromosome differentiation. Moreover, based on the reproductive characteristics of female *G. affinis*, this organism may serve as a model for the study of the transition from oviparity to viviparity and provide new perspectives and clues for issues, such as physiological, morphological, and immunological changes to the female reproductive tract.

Male *G. affinis* (ZZ type) scaffold level genome data have been published [12], and they serve as resources for comparative genomics among poecilids and teleosts. However, recently released data are not sufficient to explore the evolution of ZW sex chromosomes and the reproduction mode of female *G. affinis*. Further studies are necessary to overcome the lack of high-quality genomic data for female *G. affinis* (ZW type).

Accordingly, in this research, a chromosome (pseudo-chromosomes)-level genome assembly of female *G. affinis* was generated using Oxford nanopore, BioNano, and Hi-C technology. We used genomic data for male *G. affinis* and identified Z and W sex chromosomes. Combined with comparative genomic analysis of the Z and W chromosomes, the high-quality genome produced in this study is expected to provide the foundation for research on the differentiation of sex chromosomes. These data may also help explore the molecular basis of the morphological differences between the *G. affinis* females and males and the characteristics of ovoviviparous reproduction and may aid further studies on functional genomics.

Methods

Sample collection

Samples for genome sequencing of female *G. affinis* (Figure 1; sexual dimorphism is pronounced in *G. affinis*: the anal fin of adult females resembles the dorsal fins, while the anal fin of adult males is pointy and specialized for gonopodium) (Fishbase ID: 3215; NCBI taxonomy ID: 33528) were collected from the Chongde Lake at the Southwest University in Chongqing, China. The whole body (excluding the gut), brain, liver, heart, gills, gonads, and muscles were collected and quickly frozen in liquid nitrogen. Whole body samples were used for DNA sequencing, BioNano, and Hi-C for genome assembly, whereas other tissues were used for transcriptome sequencing. Animal research has been approved by the ethics committee of the Southwest University (IACUC No. Approved: IACUC-20190226-19), China.

DNA library construction and sequencing

Genomic DNA was extracted from the whole body (excluding the gut) using a Qiagen GenomicTip100 (Qiagen, Hilden, Germany). Illumina TruSeq Nano DNA Library Prep Kit (Illumina, USA) was used to construct an Illumina library with insert sizes of 350 bp, which were then sequenced on an Illumina NovaSeq platform (150-bp paired-end reads). The raw data were filtered using the following strategies: (i) filtered reads with adapters; (ii) removing reads with greater than or equal to 10% unidentified nucleotides (N); (iii) removing reads with more than 50% of bases having a phred quality of less than 5; (iv) removing reads with more than 10 nt aligned to the adapter, allowing less than or equal to 10% mismatches; and (v) removing putative polymerase chain reaction (PCR) duplicates generated by PCR amplification in the library construction process. Clean reads were used for subsequent *K-mer* analysis and nanopore data correction.

Approximately 8 μg of gDNA was prepared; Blue Pippin (Sage Science, Beverly, MA, USA) and Ligation sequencing 1D kit (SQK-LSK108; ONT, UK) were used for size selection (more than 10 kb) and nanopore library construction according to the manufacturer's instructions. Two nanopore libraries were constructed and sequenced on two different FlowCells using the PromethION sequencer (ONT). Subreads quality control was subsequently executed on fast5 files using ONT Albacore software (v0.8.4) [13], and the "passed filter" reads (higher quality reads) were used for subsequent analysis.

RNA library construction and sequencing

For RNA analyses, six tissues (brain, liver, heart, gills, gonads, and muscles) were extracted using an RNeasy Plus Mini Kit (Qiagen) from five individuals. The RNA purity, degradation/contamination, concentration, and integrity were measured using NanoDrop One

(Thermo Fisher Scientific, USA), 1% agarose gels, Qubit RNA Assay Kit with a Qubit 3.0 Fluorometer (Life Technologies, CA, USA), and the RNA Nano 6000 Assay Kit with a Bioanalyzer 2100 system (Agilent Technologies, CA, USA), respectively. The RNA quality criteria for the RNA samples were as follows: RNA integrity number more than 8.0 and OD 260/280 between 2.0 and 2.2. Validated RNA samples (from brain, liver, heart, gill, gonad, and muscle tissues) were used for Illumina library construction and sequencing and PacBio library preparation (pooled samples), construction, and sequencing.

For Illumina paired-end sequencing (Illumina Novaseq platform, 150-bp), the cDNA library was prepared using a TruSeq Sample Preparation Kit (Illumina). The clean data were obtained by removing reads containing adapters, reads containing poly-N, and low-quality reads from the raw data. Qualified RNA from six tissues were mixed in equal amounts, reverse-transcribed using a Clontech SMARTer PCR cDNA Synthesis Kit (TaKaRa), and subjected to PCR amplification using a PrimeSTAR GXL DNA polymerase, and the obtained 0.5–6-kb fragments were retained for PacBio sequencing library construction using a SMRTbell Template Prep Kit (Pacific Biosciences). Finally, a library for SMRT cell was sequenced using polymerase and V2.1 chemistry on a PacBio Sequel platform with 10 h of movie time.

Genomic features from K-mer analysis and nanopore assembly building

Clean reads obtained from the Illumina NovaSeq platform were applied to estimate the genome size and heterozygosity of the western mosquitofish by *K-mer* analysis (*17-mer* frequency distribution) using jellyfish (v2.0; RRID:SCR_005491) [14].

Filtered Oxford nanopore sequencing data were corrected by Nextdenovo [15] and using the following parameters: read cutoff = 3k, seed cutoff = 25k, block size = 2 g. Then, Oxford nanopore sequencing data were assembled using wtdbg (v1.2.8; RRID:SCR_017225) [16]; the

pipeline and parameters were as follows: wtdbg-1.2.8 -k 0 -p 23 -S 2, wtdbg-cns -c 3 -k 15, kbm-1.2.8 -k 0 -p 21 -S 2 -O 0, wtdbg-cns -k 13 -c 3.

BWA (v0.7.12; RRID:SCR_010910) [17] and Pilon (v1.21; RRID:SCR_014731) [18] were used to further improve the accuracy of the assembly, based on three rounds of mapping the Illumina reads back to the genome. Then, we used Benchmarking Universal Single-Copy Orthologs (BUSCO v3.0.1; RRID:SCR_015008) [19] to evaluate the completeness of the genome assembly by searching for annotated genes in the assembly.

Genome scaffolding with BioNano auxiliary assembly

High-molecular-weight DNA was isolated from the whole body (excluding the gut) and then labeled with Labeling Master Mix and DLE-1; next, the DNA was imaged automatically with a BioNano Saphyr system. BioNano raw BNX files were *de novo* assembled into genome maps with BioNano Solve [20]. The sorted and autodenoised single molecules were subjected to pairwise comparisons by RefAligner [21] to identify molecule overlaps, and consensus maps were constructed. All molecules were then mapped back to the consensus maps, and the maps were recursively refined and extended (two times).

Chromosomal-level genome assembly by Hi-C

The Hi-C library was prepared following a previously described procedure [22] with some modifications. Briefly, fresh whole-body samples (excluding the gut) were cut into 2-cm pieces and treated with 1% formaldehyde for 10 min at room temperature to induce crosslinking. The reaction was quenched by adding 2.5 M glycine to a final concentration of 0.2 M for 5 min. Nuclei were digested, marked, and ligated using 100 U *Dpn*II, biotin-14-dCTP (Invitrogen, Carlsbad, CA, USA), and T4 DNA Ligase, respectively. After incubation overnight for reverse crosslinking, the ligated DNA was sheared into 300–600-bp fragments. The DNA fragments

were blunt-end repaired and A-tailed, followed by purification through biotin-streptavidin-mediated pulldown. Finally, the Hi-C libraries were quantified and sequenced using an Illumina Hiseq platform (150-bp paired-end reads).

In total, 556 million paired-end reads were generated from the Hi-C libraries. Low-quality reads (quality scores less than 15), adaptor sequences, N ratio greater than 5% reads, and reads shorter than 30-bp were filtered out using fastp (v0.12.6; RRID:SCR_016962) [23], and the clean paired-end reads (549 million paired-end reads; 81,713,239,521 bp) were then mapped to the draft assembled sequence using bowtie2 v2.3.2 [24] to yield unique mapped paired-end reads.

As a result, 141 million uniquely mapped pair-end reads were generated, of which 76.82% were valid interaction pairs. Combined with the valid Hi-C data, we subsequently used the LACHESIS (ligating adjacent chromatin enables scaffolding *in situ*; RRID:SCR_017644) [25] *de novo* assembly pipeline to produce chromosome-level sequences with the following parameters: (1) CLUSTER MIN. RE SITES = 150; (2) CLUSTER MAX. LINK DENSITY = 2.5; (3) CLUSTER NONINFORMATIVE RATIO = 1.4; (4) ORDER MIN. N RES. IN TRUNK = 60; and (5) ORDER MIN. N RES. IN SHREDS = 60. The interaction heat map of the initial assembly results of LACHESIS was drawn, according to the interaction between different scaffolds, the position and direction of the scaffolds that obviously did not meet the chromosome interaction characteristics in the figure were adjusted. Of note, if there were situations in a scaffold itself that did not meet the chromosome interaction characteristics, the scaffold was interrupted. Next, the scaffolds were adjusted separately until the overall heat map conformed to the characteristics of chromosome interaction.

We used the same method to assemble the genome of a published male *G. affinis* (GCA_003097735.1) and obtained chromosomal-level genomic data.

Annotation of repetitive elements

Simple sequence repeat (SSR) sequences in the genome were analyzed by MISA (RRID:SCR_010765) [26]. For transposable elements (TEs), we first used RepeatModeler (v2.0.1; RRID:SCR_015027) [27], LTR_FINDER (RRID:SCR_015247) [28], and MITE-Hunter software [29], based on the principle of *de novo* methods and TE-specific architecture to build a *G. affinis* TE sequence library. The data were then combined with Repbase [30] to construct the final database. Finally, RepeatMasker (v4.0.5; RRID:SCR_012954) [31] was used to predict the TEs in male and female *G. affinis* according to the final constructed TE database.

Gene prediction and function annotation

First, for homology-based prediction, the RNA-seq bam file from mapped reads to the genome by HISAT2 (v2.1.0; RRID:SCR_015530) [32] and protein sequences from five sequenced vertebrates, *Danio rerio* (GCA_000002035.4), *Oryzias latipes* (GCA_002234715.1), *Nothobranchius furzeri* (GCA_001465895.2), *Xiphophorus maculatus* (GCA_002775205.2), and *Poecilia formosa* (GCA_000485575.1) were used to predict *G. affinis* genes by GeMoMa (v2.3; RRID:SCR_017646) [33]. Second, we used Augustus (v2.5.5; RRID:SCR 008417) [34] for *ab initio* prediction, a training set generated from the GeMoMa results and transcripts of *G. affinis*, and transcripts obtained from high-throughput data using HISAT2 combined with Stringtie (v1.3.5; RRID:SCR_016323) [35]. Full-length transcriptome data were used to construct consensus sequences through clustering with IsoSeq3 [36]. These sequences were then compared with reference genomes using GMAP (RRID:SCR_008992) [37]; next, both transcripts were integrated to remove redundancy, and the results were then processed with PASA (RRID:SCR_014656) [38] to obtain the final results. Augustus' predictions were compared with the Pfam database [39] to remove genes without domains, and the results of

Augustus and GeMoMa were further removed by alternative splicing and integrated. Finally, the TransposonPSI [40] alignment was used to remove sequences containing transposons, yielding the final results.

Functional annotation of the predicted genes of *G. affinis* was performed by alignment to the SwissPro [41], TrEMBL [41], Kyoto Encyclopedia of Genes and Genomes (KEGG) [42], and gene ontology (GO) [43] databases using BLAST (v2.3.0; RRID:SCR_004870) and KAAS (v2.1) [44]. Motifs and domains were annotated using InterProScan (v5.24; RRID:SCR_005829) [45].

Noncoding RNA prediction

rRNAs, small nuclear RNAs (snRNAs), microRNAs (miRNAs), and tRNAs, were identified by adopting Infernal v1.1.2 [46] using the Rfam database (release 13.0) [47] for the *G. affinis* genome using BLASTN (E-value ≤ 1e−5; RRID:SCR_001598) [48]. tRNAs were predicted using tRNAscan-SE (v1.3.1; RRID:SCR_010835) [49] with default parameters for eukaryotes. The rRNAs and their subunits were predicted using RNAmmer (v1.2; RRID:SCR_017075) [50].

Evolutionary and comparative genomic analyses

We used OrthoMCL (version 2.0.9; RRID:SCR 007839) [51] to cluster the female *G. affinis* annotated genes with an e-value cutoff of 1e–5 and Markov Chain Clustering with default inflation parameters for an all-to-all BLASTP (RRID:SCR 001010) analysis of entries for the reference genomes of 11 fishes, including *G. affinis* in this study and other 10 published fishes reported to date (*P. reticulate*, *P. formosa*, *P. latipinna*, *P. mexicana*, *X. couchianus*, *N. furzeri*, *Cyprinodon variegatus*, *Fundulus heteroclitus*, *Lepisosteus oculatus*, and *Oreochromis niloticus*). Computational Analysis of gene Family Evolution (CAFE; v4.0.1;

RRID:SCR_005983) [52] was used to identify expanded and contracted gene families in *G. affinis*, and these data were then used for GO and KEGG enrichment analyses.

The orthologous genes obtained from the above analyses was subjected to multiple sequence alignment using Mafft (v7.313; RRID:SCR_011811) [53] and Gblocks (v0.91b; RRID:SCR_015945) [54] to extract conserved sites based on the GTRGAMMA model and RAxML (v 8.2.11; RRID:SCR_006086) [55]. Using this tree, MCMCTREE in PAML (v4.9e; RID:SCR_014932) [56] was applied to estimate the 95% confidence intervals of the differentiation times, where the published timings for the divergence of difference species were obtained with the TimeTree database [57].

The orthologous genes were then used in PAML codon substitution models and likelihood ratio tests (codeml) based on the branch-site model to calculate Ka and Ks, yielding positively selected genes, which were then utilized for GO and KEGG enrichment analyses.

Recognition and comparison of the W and Z chromosomes of G. affinis

Mummer (v3.0; RRID:SCR_018171) [58] was used for aligning entire genomic DNA sequences from *X. couchianus*, and male and female *G. affinis*, to make the chromosome numbering system of both species the same. The W chromosome was identified by the specificity of the female *G. affinis* W chromosome length, and then, the Z chromosome in the male *G. affinis* was identified based on synteny. Mummer was also used for aligning entire genomic DNA sequences from the Z and W chromosomes, and circos plot distributions of homologous sequence pairs among the Z and W chromosome pairs were plotted using Circos (v0.69-6; RRID:SCR_011798) [59].

According to the results of the RepeatMasker analysis of female and male *G. affinis* genomes, the length and distribution of TEs on chromosomes Z and W were compared.

OrthoFinder (v2.3.8; RRID:SCR_017118) [60] was used to compare the genes on chromosomes Z and W.

TE insertion time analyses

We calculated female and male *G. affinis* TE insertion times in genomes using the algorithm T = K / 2r, where K is the Kimura distance-based copy divergence of TEs and r is the nucleic acid substitution rate. The K value was obtained from RepeatMasker. To estimate r values for *G. affinis*, we used LASTZ (v1.04.00; RRID:SCR_018556) [61] , chainNet (v2) [62], and MULTIZ (v11.2) [63], along with genomes used in evolutionary analyses and the female *G. affinis* genome as a reference sequence. With the whole-genome alignments, we used the msa_view tool in the PHAST package (v1.2.1; RRID:SCR_003204) [64] to extract 4D site alignments, based on the female *G. affinis* gene annotations. The phyloFit program in the PHAST package was used to estimate the phylogenetic model, with tree topology (result of evolutionary analyses) as an input parameter. The branch length results were represented as units of substitutions per site. We calculated the root-to-tip substitution rates from the most recent common ancestor of selected species to each fish lineage, and then divided the root-to-tip substitution rates by the divergence time (Divergence time: 314.47 Mya, 314.47 Million years ago) of most recent common ancestor of selected species.

Results and Discussion

Female G. affinis genome initial characteristics

A total of 30.5 Gb Illumina clean reads were used for analyzing female *G. affinis* genome size and heterozygosity using *K-mer* analysis. Based on 26,474,864,304 *17-mers* and a peak *17-mer* depth of 37 (Figure S1), the estimated heterozygosity rate was approximately 0.42%, and the estimated genome size of female *G. affinis* was about 715 Mb. Of note, the estimated

genome size is similar to that of the nuclear DNA content estimated in a previous study using flow cytometry (0.75 pg, approximately 733 Mb) [65].

De novo assembly of a female G. affinis reference genome

Next, 74.4 Gb (2,866,145 reads, average read length of 25.98 Kb, N50 35.86 Kb, and longest read length of 273 Kb) Oxford nanopore clean long reads were used to construct the reference genome. We obtained a 662-Mb genomic DNA sequences by assembly with a contig N50 length of 12.9 Mb.

The long reads assembly result consisted of 217 contigs, and the longest contig was 28.6 Mb. Then, BUSCO was used to assess the completeness of the assembled genome. Approximately 97.2% of the complete genes could be detected in the genome of female *G. affinis*, confirming the completeness of the genome. Assembly results of long reads scaffolds obtained using optical maps were assembled with 80-Gb BioNano molecules. The final assembly contained 125 scaffolds with a scaffold N50 size of 26.4 Mb. Finally, we used the Hi-C technique to anchor the assembly scaffolds in 24 chromosomes of female *G. affinis* (Table S1). We found that 141,528,181 unique mapped paired-end reads were generated and occupied approximately 51.5% of the total clean paired-end reads (274,658,176). Then, the frequency of scaffold interactions was estimated on the basis of the pairs mapped to the scaffolds. We found that 113 scaffolds were successfully anchored in 24 chromosomes (Figure 2, Figure S2a), consistent with the records of the chromosome number by cytogenetic analysis [10, 66], representing 90.4% of all scaffolds and 97.9% of all scaffold nucleotide bases. The total assembly size of the chromosomes was approximately 679.4 Mb (Table 1). In the male *G. affinis* genomic DNA sequences, 734 scaffolds were successfully anchored in 24 chromosomes (Figure 2, Figure S2b), and the total assembly size was 592.7 Mb.

Genome annotation

Assembled chromosome-level genome of female *G. affinis* were used to predict repeat sequences. In total, 5,630,271 SSRs were identified, including 5,478,552 mono-, 100,272 di-, 28,431 tri-, 19,721 tetra-, 2,048 penta-, and 1,247 hexa-nucleotide repeats. Overall, the combined the homology-based and *de novo* prediction results indicated that TEs accounted for 22.54% of the assembly genome (Table S2). Additionally, class I TEs (RNA transposons) occupied approximately 5.15% of the assembly genome. The most abundant RNA transposons found in the *G. affinis* assembly genome were long interspersed nuclear elements, which constituted 54.37% of all identified RNA transposons. The female *G. affinis* genome was very rich in class II TEs (DNA transposons), which occupied almost 11.83% of genome content.

For genome annotation, 23,997 protein-coding genes were predicted in the female *G. affinis* genome. Compared with other existing published poeciliid fish annotated information, a number of genes in female *G. affinis* was similar to those in *P. formosa* (23,615 genes) and *X. maculatus* (23,628 genes) (Table S3 and Figure S3). BUSCO gene prediction was carried out using the actinopterygii_odb9 single-copy homologous gene to predict the existing sequence of the genome. Approximately 97% of complete gene components could be found in this gene set, indicating that most of the conserved genes were well predicted and that the prediction results were relatively reliable (Table S4). Finally, 23,737 genes were annotated in at least one of the databases (KOG, KEGG, NR, SwissProt, GO), and up to 98.92% of *G. affinis* genes were functionally annotated (Table S5). Finally, 143 snRNAs, 220 rRNAs, 371 miRNAs, and 3885 tRNAs were also identified.

Genome evolution

In order to determine the evolutionary relationships between *G. affinis* and other vertebrates, a phylogenetic tree was reconstructed on the basis of 6,457 single-copy

orthologous genes from 10 other vertebrate genomes (Figure 3a). *L. oculatus* and *O. niloticus* were used as outgroups. As a species of the family Poeciliidae, *G. affinis* clustered into one branch with other fish from Poeciliidae. Compared with six other sequenced members of the Poeciliidae family, *G. affinis* had a closer relationship with *X. couchianus*, consistent with previously published phylogenies [67]. Next, a timetree was created based on the above 6,457 single-copy orthologous genes, and the estimated divergence time between *G. affinis* and *X. couchianus* was found to be approximately 16.57 million years ago (Mya; Figure 3; Figure S3). In addition, the divergence time between *G. affinis* and four other members of the Poeciliidae family was approximately 22.75 Mya.

In order to examine the evolutionary history of gene families, we performed gene family expansion and contraction analysis with the female *G. affinis* genes. We found 652 expansion gene families and 1,046 contraction gene families (Figure 3b). Expansion gene families were enriched in 44 GO (Table S6) categories and 34 KEGG pathways (Table S7), most of which were related to oxygen metabolism, olfactory pathways, and visual pathways. Next, codeml was used to calculate the average Ka/Ks values and conduct branch-site likelihood ratio analyses to detect positively selected genes in the female *G. affinis* genome. The results showed that there were 590 positively selected genes in the female *G. affinis* genome. The positively selected genes were enriched in 12 GO categories (aspartic-type endopeptidase activity, DNA repair, microtubule binding, insulin-like growth factor binding, tRNA aminoacylation for protein translation, microtubule motor activity, rRNA processing, microtubule-based movement, protein dephosphorylation, protein tyrosine phosphatase activity, chromatin binding, and nucleus) and three KEGG pathways (complement and coagulation cascades, peroxisome, and platelet activation).

Recognition and evolution of sex chromosomes

Genetic controlled sex determination systems in fish are variable, ranging from XX/XY to ZZ/ZW [68]. Fish generally do not have highly morphologically differentiated sex chromosomes, making it difficult to distinguish between autosomes and sex chromosomes. Hence, there are only a few fish species for which there is known information on sex determination mechanisms and sex chromosome systems. Therefore, a suitable experimental model is required for the identification and elucidation of the mechanisms of fish sex chromosome evolution, and the female *G. affinis* is a suitable and consistent model.

Early karyotype analysis demonstrated that female *G. affinis* shows heterogamy of the ZW type, and its W chromosome is much longer than other chromosomes [10, 66]. The longest sequence was selected from the assembly results at the chromosome level as the W candidate chromosome, and one female-specific DNA marker [69] was used for confirmation. In the end, the marker has been aligned to the W candidate chromosome, but was not found in the genomic DNA sequences of the male *G. affinis*. Analysis of the synteny of the whole genomes of female and male mosquitofish by Mummer demonstrated that the Z chromosome was also present in the male *G. affinis* genomic DNA sequences (Figure 2). By comparing the Z and W chromosomes (Figure 4a), we found the length of the W and Z chromosome repeat sequences to be approximately 5.0 Mb and 8.5 Mb, respectively. Among them, the length and content of the *Helitron* superfamily of the two chromosomes (Z: 591,639 bp, 1.3%; W: 6,6868 bp, 0.24%) were significantly different (Results have been submitted to GigaDB). There were 1279 and 1027 genes on the W and Z chromosomes, respectively. Homologous analysis showed that there were 712 one-to-one pairs. There were 118 and 203 genes on the W and Z chromosomes unassigned to any gene groups, and the others were of the one-to-many and many-to-many types; these results provide research directions for our future analyses on functional genomics (Results have been submitted to GigaDB).

Some researchers have studied the role of transposons in sex chromosome differentiation, and they found that TEs seem to be play an important role in the evolution of sex chromosomes, with their accumulation and loss having huge effects on the lengths of sex chromosomes [70-72]. However, differences in TE contents between Z and W chromosomes alone cannot determine the true course of differentiation, e.g., whether the increased length of the W chromosome compared with that of the Z chromosome is caused by extension of the W chromosome or by degeneration of the Z chromosome. There is no substantial evidence to explain this observation. Therefore, the introduction of the time factor is extremely important. *G. holbrooki* and *G. affinis* are so closely related that for a long time, biologists thought they were the same species. Phylogenetic analyses estimated that their divergence time was approximately 2–7 Mya [69, 73, 74], and other researchers showed that the XY and ZW sex determination mechanisms had independent origins in *G. holbrooki* and *G. affinis*, respectively [68]. Therefore, we speculate that the differentiation of Z and W sex chromosomes is a very recent event. Additionally, previous studies suggested that this process may be enriched on the W chromosome by TEs, leading to an increase in the sex chromosome size during the early phase of differentiation and the subsequent reduction in size later during evolution [75]. If this hypothesis is correct, then we should be able to observe a large number of transposons inserted in the W chromosome in the recent past (between 2–7 Mya). Indeed, our results indicated very recent mass insertion events of TEs into the W chromosome (Figure 4b), and the insertion time characteristics of the TEs into the W chromosome were specific because its insertion time trends were dramatically different from those of autosomal and Z-chromosomal TEs (Figure 4c and Figure S5). Moreover, we speculate that most of the long gaps (Figure 4a) on the W chromosome were also caused by the aggregation of too many highly similar TE sequences to form TE clusters through the recent activation of TEs. Thus, we expected that the TE content of the W chromosome of *G. affinis* should be much higher than that observed till date.

Accordingly, our results showed that the cause of sex chromosome differentiation in female *G. affinis* was likely to be related to extension of the W chromosome.

Conclusions

In this study, we assembled the chromosome-level female western mosquitofish genome using the most mainstream technology available. In terms of parameters such as contig N50, scaffold N50, and gene annotation number, these are high-quality genomic data. Evolutionary analysis provides ideas for future work, for example, oxygen transport in mosquitofish deserves attention. We conducted a preliminary study on W and Z sex chromosome differentiation based on the specificity of the sex chromosome in female western mosquitofish and provided data to support the previous hypothesis that a longer W chromosome is associated with the activity (insertion) of TEs. In conclusion, our high-quality genomic data lay the foundation for the study of chromosome evolution, reproductive characteristics, and sexual dimorphism in western mosquitofish.

Availability of supporting data

The raw genome and RNA sequencing data were deposited in the SRA under Bioproject number PRJNA599452. The chromosome-level genome, annotation, and other supporting data are also available via the GigaScience database, *GigaDB* [76].

Abbreviations

BLAST: Basic Local Alignment Search Tool; bp: base pairs; BUSCO: Benchmarking Universal Single-Copy Orthologs; BWA: Burrows-Wheeler Aligner; CAFE: Computational Analysis of gene Family Evolution ; GeMoMa: Gene Model Mapper; Gb: gigabase pairs; GO: Gene Ontology; Hi-C: High-throughput chromosome conformation capture; KASS: KEGG Automatic Annotation Server; kb: kilobase pairs; KEGG: Kyoto Encyclopedia of Genes and Genomes; Mb: megabase pairs; MRCA: most recent common ancestor; LTR: long terminal repeat; NCBI: National Center for Biotechnology Information; PAML: Phylogenetic Analysis by Maximum Likelihood; PASA: Program to Assemble Spliced Alignments; RAxML: Randomized Accelerated Maximum Likelihood; RNA-seq: RNA sequencing; TE: transposable element.

Competing Interests

The authors declare that there have no competing interests.

Author Contributions

F.S. performed the major part of data analysis and drafted the manuscript. Y.M. and N.L. contributed to samples collection and drafted the manuscript. Z.P. and L.A. contributed to research design and final edits to the manuscript. All authors read and approved the final manuscript.

Acknowledgements

We are grateful to Drs. Aurélie Kapusta, Guojie Zhang, and Cai Li for help with estimating substitution rate and transposon insertion time. We thank Drs. Wen Wang, Lei Chen, Kun Wang, Ru Zhang, Botong Zhou, Zeshan Lin, and Bao Wang for discussion. We also thank Tian Lu for his help with preparing the fish image. This work was supported by the grants from the National Key Research and Development Program of China (2018YFD0900805), the National Natural Science Foundation of China (31872204), the Fundamental Research Funds for the Central Universities (XDJK2020C043), and the Natural Science Foundation of Chongqing Postdoctoral Science Foundation (cstc2019jcyj-bshX0071).

References

1. Russo G, Chou A, Rettig JE and Smith GR. Foraging responses of mosquitofish (*Gambusia affinis*) to items of different sizes and colors. J Freshw Ecol. 2008;23 4:677-8. doi:10.1080/02705060.2008.9664256.
2. Cote J, Fogarty S, Weinersmith K, Brodin T and Sih A. Personality traits and dispersal tendency in the invasive mosquitofish (*Gambusia affinis*). Proc Biol Sci. 2010;277 1687:1571-9. doi:10.1098/rspb.2009.2128.
3. Smith GR and Smith LE. Effects of Western Mosquitofish (*Gambusia affinis*) on tadpole production of Gray Treefrogs (*Hyla versicolor*). Herpetol Conserv Biol. 2015;10 2:723-7.
4. Merkley SS, Rader RB and Schaalje GB. Introduced Western Mosquitofish (*Gambusia affinis*) reduce the emergence of aquatic insects in a desert spring. Freshw Sci. 2015;34 2:564-73. doi:10.1086/680381.
5. Raut SA and Angus RA. Triclosan has endocrine‐disrupting effects in male western mosquitofish, *Gambusia affinis*. Environ Toxicol Chem. 2010;29 6:1287-91. doi:10.1002/etc.150.
6. Brockmeier EK, Ogino Y, Iguchi T, Barber DS and Denslow ND. Effects of 17beta-trenbolone on Eastern and Western mosquitofish (*Gambusia holbrooki* and *G. affinis*) anal fin growth and gene expression patterns. Aquat Toxicol. 2013;128-129:163-70. doi:10.1016/j.aquatox.2012.12.007.
7. Vera M, Díez‐del‐Molino D and García‐Marín JL. Genomic survey provides insights into the evolutionary changes that occurred during European expansion of the invasive mosquitofish (*Gambusia holbrooki*). Mol Ecol. 2016;25 5:1089-105. doi:10.1111/mec.13545.

8. Diez-del-Molino D, Carmona-Catot G, Araguas RM, Vidal O, Sanz N, Garcia-Berthou E, et al. Gene flow and maintenance of genetic diversity in invasive mosquitofish (*Gambusia holbrooki*). PLoS ONE. 2013;8 12:e82501. doi:10.1371/journal.pone.0082501.

9. Vidal O, Sanz N, Araguas RM, Fernández‐Cebrian R, Diez‐del‐Molino D and García‐Marín JL. SNP diversity in introduced populations of the invasive *Gambusia holbrooki*. Ecol Freshw Fish. 2012;21 1:100-8. doi:10.1111/j.1600- 0633.2011.00527.x.

10. Black DA and Howell WM. The North American mosquitofish, *Gambusia affinis*: a unique case in sex chromosome evolution. Copeia. 1979; 1979:509-13. doi:10.2307/1443231.

11. Irwin DE. Sex chromosomes and speciation in birds and other ZW systems. Mol Ecol. 2018;27 19:3831-51. doi:10.1111/mec.14537.

12. Hoffberg SL, Troendle NJ, Glenn TC, Mahmud O, Louha S, Chalopin D, et al. A High-Quality Reference Genome for the Invasive Mosquitofish *Gambusia affinis* Using a Chicago Library. G3-Genes Genom Genet. 2018;8 6:1855-61. doi:10.1534/g3.118.200101.

13. Albacore. https://community.nanoporetech.com/protocols/albacore-offline-basecalli/v/abec_2003_v1_revan_29nov2016/linux. Accessed May 27, 2019.

14. Marcais G and Kingsford C. A fast, lock-free approach for efficient parallel counting of occurrences of *k-mers*. Bioinformatics. 2011;27 6:764-70. doi:10.1093/bioinformatics/btr011.

15. NextDenovo. https://github.com/Nextomics/NextDenovo/. Accessed July 21, 2019.

16. Ruan J and Li H. Fast and accurate long-read assembly with wtdbg2. Nat Methods. 2019; 17 2:155-8. doi:10.1038/s41592-019-0669-3.

17. Li H and Durbin R. Fast and accurate short read alignment with Burrows-Wheeler transform. Bioinformatics. 2009;25 14:1754-60. doi:10.1093/bioinformatics/btp324.

18. Walker BJ, Abeel T, Shea T, Priest M, Abouelliel A, Sakthikumar S, et al. Pilon: an integrated tool for comprehensive microbial variant detection and genome assembly improvement. PLoS ONE. 2014;9 11:e112963. doi:10.1371/journal.pone.0112963.

19. Simao FA, Waterhouse RM, Ioannidis P, Kriventseva EV and Zdobnov EM. BUSCO: assessing genome assembly and annotation completeness with single-copy orthologs. Bioinformatics. 2015;31 19:3210-2. doi:10.1093/bioinformatics/btv351.

20. BioNano Solve. http://bnxinstall.com/solve/BionanoSolveInstall.html. Accessed August 21, 2019.

21. RefAligner. https://bionanogenomics.com/wp-content/uploads/2017/03/RefAligner.msi_.zip. Accessed August 21, 2019.

22. Belton JM, McCord RP, Gibcus JH, Naumova N, Zhan Y and Dekker J. Hi-C: a comprehensive technique to capture the conformation of genomes. Methods. 2012;58 3:268-76. doi:10.1016/j.ymeth.2012.05.001.

23. Chen S, Zhou Y, Chen Y and Gu J. fastp: an ultra-fast all-in-one FASTQ preprocessor. Bioinformatics. 2018;34 17:i884-i90. doi:10.1093/bioinformatics/bty560.

24. Langmead B and Salzberg SL. Fast gapped-read alignment with Bowtie 2. Nat Methods. 2012;9 4:357-9. doi:10.1038/nmeth.1923.

25. Burton JN, Adey A, Patwardhan RP, Qiu R, Kitzman JO and Shendure J. Chromosome-scale scaffolding of de novo genome assemblies based on chromatin interactions. Nat Biotechnol. 2013;31 12:1119-25. doi:10.1038/nbt.2727.

26. Beier S, Thiel T, Munch T, Scholz U and Mascher M. MISA-web: a web server for microsatellite prediction. Bioinformatics. 2017;33 16:2583-5. doi:10.1093/bioinformatics/btx198.

27. RepeatModeler. http://www.repeatmasker.org/RepeatModeler. Accessed July 27, 2019.
28. Xu Z and Wang H. LTR_FINDER: an efficient tool for the prediction of full-length LTR retrotransposons. Nucleic Acids Res. 2007;35:W265-8. doi:10.1093/nar/gkm286.
29. Han Y and Wessler SR. MITE-Hunter: a program for discovering miniature inverted-repeat transposable elements from genomic sequences. Nucleic Acids Res. 2010;38 22:e199. doi:10.1093/nar/gkq862.
30. Bao W, Kojima KK and Kohany O. Repbase Update, a database of repetitive elements in eukaryotic genomes. Mob DNA. 2015;6:11. doi:10.1186/s13100-015-0041-9.
31. RepeatMasker. http://www.repeatmasker.org/RMDownload.html. Accessed July 27, 2019.
32. Kim D, Langmead B and Salzberg SL. HISAT: a fast spliced aligner with low memory requirements. Nat Methods. 2015;12 4:357-60. doi:10.1038/nmeth.3317.
33. Keilwagen J, Wenk M, Erickson JL, Schattat MH, Grau J and Hartung F. Using intron position conservation for homology-based gene prediction. Nucleic Acids Res. 2016;44 9:e89. doi:10.1093/nar/gkw092.
34. Stanke M, Waack S. Gene prediction with a hidden Markov model and a new intron submodel. Bioinformatics. 2003;19(suppl 2):215-25. doi:10.1093/bioinformatics/btg1080.
35. Pertea M, Pertea GM, Antonescu CM, Chang TC, Mendell JT and Salzberg SL. StringTie enables improved reconstruction of a transcriptome from RNA-seq reads. Nat Biotechnol. 2015;33 3:290-5. doi:10.1038/nbt.3122.
36. IsoSeq3. https://github.com/PacificBiosciences/IsoSeq3. Accessed July 30, 2019.
37. Wu TD and Watanabe CK. GMAP: a genomic mapping and alignment program for mRNA and EST sequences. Bioinformatics. 2005;21 9:1859-75. doi:10.1093/bioinformatics/bti310.

38. Haas BJ, Delcher AL, Mount SM, Wortman JR, Smith RK Jr, Hannick LI, et al. Improving the Arabidopsis genome annotation using maximal transcript alignment assemblies. Nucleic Acids Res. 2003;31 19:5654-66. doi: 10.1093/nar/gkg770.

39. Finn RD, Bateman A, Clements J, Coggill P, Eberhardt RY, Eddy SR, et al. Pfam: the protein families database. Nucleic Acids Res. 2014;42:D222-30. doi: 10.1093/nar/gkt1223.

40. TransposonPSI. http://transposonpsi.sourceforge.net/. Accessed August 30, 2019.

41. Apweiler R, Bairoch A, Wu CH, et al. UniProt: the universal protein knowledgebase. Nucleic Acids Res 2017;45:D158-69. doi:10.1093/nar/gkw1099.

42. The Gene Ontology Consortium. Expansion of the Gene Ontology knowledgebase and resources. Nucleic Acids Res 2017;45:D331-8. doi:10.1093/nar/gkw1108.

43. Kanehisa M, Goto S, Sato Y, et al. Data, information, knowledge and principle: back to metabolism in KEGG. Nucleic Acids Res 2014;42:D199-205. doi:10.1093/nar/gkt1076.

44. Moriya Y, Itoh M, Okuda S, Yoshizawa AC and Kanehisa M. KAAS: an automatic genome annotation and pathway reconstruction server. Nucleic Acids Res. 2007;35:W182-5. doi:10.1093/nar/gkm321.

45. Jones P, Binns D, Chang HY, Fraser M, Li W, McAnulla C, et al. InterProScan 5: genome-scale protein function classification. Bioinformatics. 2014;30 9:1236-40. doi:10.1093/bioinformatics/btu031.

46. Nawrocki EP and Eddy SR. Infernal 1.1: 100-fold faster RNA homology searches. Bioinformatics. 2013;29 22:2933-5. doi:10.1093/bioinformatics/btt509.

47. Kalvari I, Argasinska J, Quinones-Olvera N, Nawrocki EP, Rivas E, Eddy SR, et al. Rfam 13.0: shifting to a genome-centric resource for non-coding RNA families. Nucleic Acids Res. 2018;46 D1:D335-42. doi:10.1093/nar/gkx1038.

48. Camacho C, Coulouris G, Avagyan V, Ma N, Papadopoulos J, Bealer K, et al. BLAST+: architecture and applications. BMC Bioinformatics. 2009;10:421. doi:10.1186/1471-2105-10-421.
49. Lowe TM and Eddy SR. tRNAscan-SE: a program for improved detection of transfer RNA genes in genomic sequence. Nucleic Acids Res. 1997;25 5:955-64. doi:10.1093/nar/25.5.955.
50. Lagesen K, Hallin P, Rodland EA, Staerfeldt HH, Rognes T and Ussery DW. RNAmmer: consistent and rapid annotation of ribosomal RNA genes. Nucleic Acids Res. 2007;35 9:3100-8. doi:10.1093/nar/gkm160.
51. Li L, Stoeckert CJ, Jr. and Roos DS. OrthoMCL: identification of ortholog groups for eukaryotic genomes. Genome Res. 2003;13 9:2178-89. doi:10.1101/gr.1224503.
52. De Bie T, Cristianini N, Demuth JP and Hahn MW. CAFE: a computational tool for the study of gene family evolution. Bioinformatics. 2006;22 10:1269-71. doi:10.1093/bioinformatics/btl097.
53. Yamada KD, Tomii K, Katoh K. Application of the MAFFT sequence alignment program to large data-reexamination of the usefulness of chained guide trees. Bioinformatics. 2016;32 21:3246-51. doi: 10.1093/bioinformatics/btw412.
54. Gblocks. http://molevol.cmima.csic.es/castresana/Gblocks.html. Accessed September 5, 2019.
55. Stamatakis A. RAxML version 8: a tool for phylogenetic analysis and post-analysis of large phylogenies. Bioinformatics. 2014;30 9:1312-3. doi:10.1093/bioinformatics/btu033.
56. Yang Z. PAML: a program package for phylogenetic analysis by maximum likelihood. Comput Appl Biosci. 1997;13 5:555-6. doi:10.1093/bioinformatics/13.5.555.
57. TIMETREE. http://www.timetree.org. Accessed September 10, 2019.

58. Kurtz S, Phillippy A, Delcher AL, Smoot M, Shumway M, Antonescu C, et al. Versatile and open software for comparing large genomes. Genome Biol. 2004;5 2:R12. doi:10.1186/gb-2004-5-2-r12.
59. Krzywinski M, Schein J, Birol I, Connors J, Gascoyne R, Horsman D, et al. Circos: an information aesthetic for comparative genomics. Genome Res. 2009;19 9:1639-45. doi:10.1101/gr.092759.109.
60. Emms DM and Kelly S. OrthoFinder: phylogenetic orthology inference for comparative genomics. Genome Biol. 2019;20 1:238. doi:10.1186/s13059-019-1832-y.
61. Kent WJ, Baertsch R, Hinrichs A, Miller W and Haussler D. Evolution's cauldron: duplication, deletion, and rearrangement in the mouse and human genomes. Proc Natl Acad Sci U S A. 2003;100 20:11484-9. doi:10.1073/pnas.1932072100.
62. LASTZ. http://www.bx.psu.edu/~rsharris/lastz/. Accessed September 20, 2019.
63. Blanchette M, Kent WJ, Riemer C, Elnitski L, Smit AF, Roskin KM, et al. Aligning multiple genomic sequences with the threaded blockset aligner. Genome Res. 2004;14 4:708-15. doi:10.1101/gr.1933104.
64. Hubisz MJ, Pollard KS and Siepel A. PHAST and RPHAST: phylogenetic analysis with space/time models. Brief Bioinform. 2011;12 1:41-51. doi:10.1093/bib/bbq072.
65. Tiersch TR, Chandler RW, Wachtel SS and Elias S. Reference-Standards for Flow-Cytometry and Application in Comparative Studies of Nuclear-DNA Content. Cytometry. 1989;10 6:706-10. doi:10.1002/cyto.990100606.
66. Chen T and Ebeling A. Karyological evidence of female heterogamety in the mosquitofish, *Gambusia affinis*. Copeia. 1968; 1968:70-5. doi:10.2307/1441552.
67. Furness AI, Pollux BJA, Meredith RW, Springer MS and Reznick DN. How conflict shapes evolution in poeciliid fishes. Nat Commun. 2019;10 1:3335. doi:10.1038/s41467-019-11307-5.

68. Kottler VA, Feron R, Nanda I, Klopp C, Du K, Kneitz S, et al. Independent Origin of XY and ZW Sex Determination Mechanisms in Mosquitofish Sister Species. Genetics. 2020;214 1:193-209; doi:10.1534/genetics.119.302698.

69. Lamatsch DK, Adolfsson S, Senior AM, Christiansen G, Pichler M, Ozaki Y, et al. A transcriptome derived female-specific marker from the invasive Western mosquitofish (*Gambusia affinis*). PLoS ONE. 2015;10 2:e0118214. doi:10.1371/journal.pone.0118214.

70. Rosolen LAM, Vicari MR and Almeida MC. Accumulation of Transposable Elements in Autosomes and Giant Sex Chromosomes of *Omophoita* (Chrysomelidae: Alticinae). Cytogenet Genome Res. 2018;156 4:215-22. doi:10.1159/000495199.

71. Erlandsson R, Wilson JF and Paabo S. Sex chromosomal transposable element accumulation and male-driven substitutional evolution in humans. Mol Biol Evol. 2000;17 5:804-12. doi:10.1093/oxfordjournals.molbev.a026359.

72. Dechaud C, Volff JN, Schartl M and Naville M. Sex and the TEs: transposable elements in sexual development and function in animals. Mob DNA. 2019;10:42. doi:10.1186/s13100-019-0185-0.

73. Lydeard C, Wooten MC and Meyer A. Molecules, morphology, and area cladograms: a cladistic and biogeographic analysis of *Gambusia* (Teleostei: Poeciliidae). Syst Biol. 1995;44 2:221-36. doi:10.1093/sysbio/44.2.221.

74. Helmstetter AJ, Papadopulos AS, Igea J, Van Dooren TJ, Leroi AM and Savolainen V. Viviparity stimulates diversification in an order of fish. Nat Commun. 2016;7:11271. doi:10.1038/ncomms11271.

75. Schartl M, Schmid M and Nanda I. Dynamics of vertebrate sex chromosome evolution: from equal size to giants and dwarfs. Chromosoma. 2016;125 3:553-71. doi:10.1007/s00412-015-0569-y.

76. Shao F, Ludwig A, Mao Y, Liu N and Peng Z. Supporting data for "Chromosome-level genome assembly of the female western mosquitofish (*Gambusia affinis*)". GigaScience Database. 2020. http://doi.org/10.5524/100778

Table 1. Genome assembly statistics of *Gambusia affinis*.

	Nanopore	BioNano	Hi-C (♀)[#]	Hi-C (♂)[##]
Total assembly size of contig/scaffold/chromosome (bp)	662,579,534	680,140,492	679,423,294[*]	592,666,412[*]
Number of contig/scaffold/chromosome	217	125	24	24
N50 contig/scaffold/chromosome length (bp)	12,906,370	26,455,434	29,761,488	25,946,590
N90 contig/scaffold/chromosome length (bp)	1,629,223	18,394,109	23,709,503	21,272,223
Longest contig/scaffold/chromosome (bp)	28,665,999	31,542,956	45,125,082	30,583,032

* The length of 24 chromosomes, excluding the length of the unanchored sequences.

[#] Based on the data generated in this study.

[##] Based on published data [12].

Figure Legends

Figure 1. Image of female (top) and male (bottom) *G. affinis*.

Figure 2. Genomic synteny of *X. couchianus*, female *G. affinis*, and male *G. affinis*. Female *G. affinis* LG01 represents the W chromosome and male *G. affinis* LG01 represents the Z chromosome.

Figure 3. Phylogenetic and evolutionary analysis of *G. affinis.* (a) Divergence time estimates and gene clusters in *G. affinis* and other species. (b) Expansion and contraction of *G. affinis* gene families. MRCA represents most recent common ancestor, and circle diagrams and numbers below represent the proportion and specific values of the gene families of expansion and contraction, respectively (red represents contraction, green represents expansion).

Figure 4. Comparative genomic analysis of the Z and W chromosomes. (a) Circos plot of Z and W chromosome alignment; the red region represents the repeat sequence density, and the green region represents the GC density. (b) Distribution of the transposon activity time for the W chromosome. (c) Distribution of transposon activity time for the Z chromosome.

Additional files

Table S1: Result of female and male *Gambusia affinis* genomic assembly at chromosome-level.

Table S2: Transposable elements (TEs) annotation in the female *Gambusia affinis* genome.

Table S3: Comparative analysis of the annotated gene set of female *Gambusia affinis* with those of five teleosts.

Table S4: Assessment of female *Gambusia affinis* genome completeness by BUSCO.

Table S5: Statistics for gene function annotation in female *Gambusia affinis* genome.

Table S6: Expansion gene families of female *Gambusia affinis* were enriched in 44 GO categories.

Table S7: Expansion gene families of female *Gambusia affinis* were enriched in 34 KEGG pathways.

Figure S1: Frequency distribution of the 17-mer graph analysis used to estimate the size of female *Gambusia affinis*.

Figure S2: Western mosquitofish genome scaffold contact matrix using Hi-C data. (a) Female western mosquitofish. (b) Male western mosquitofish. The color bar indicates the contact density from red (high) to white (low).

Figure S3: The comparisons of CDS length, exon length, exon-number, gene length, intro length, and intron number in the genomes of female *Gambusia affinis* and other teleosts.

Figure S4: Divergence time of *Gambusia affinis* and other fish species.

Figure S5: Distribution of transposon activity time for different autosomes of female *Gambusia affinis*.

Additional File: All genes located on the Z and W sex chromosomes with their locations.

Manuscript Click here to access/download;Manuscript;GigaScience_track change.docx

Chromosome-level genome assembly of the female western mosquitofish (*Gambusia affinis*)

Feng Shao[1], Arne Ludwig[2,3], Yang Mao[1], Ni Liu[1], Zuogang Peng[1, *]

[1] Key Laboratory of Freshwater Fish Reproduction and Development (Ministry of Education), Southwest University School of Life Sciences, Chongqing 400715, China

[2] Department of Evolutionary Genetics, Leibniz-Institute for Zoo and Wildlife Research, 10315 Berlin, Germany

[3] Albrecht Daniel Thaer-Institute, Faculty of Life Sciences, Humboldt University Berlin, 10115 Berlin, Germany

Running title: Genome assembly in *G. affinis*

*Correspondence: ~~pzg@swu.edu.cn~~pzg@swu.edu.cn

ORCID:

Feng Shao https://orcid.org/0000-0002-4648-2493

Arne Ludwig https://orcid.org/0000-0001-7249-9953

Zuogang Peng https://orcid.org/0000-0001-8810-2025

Abstract

Background

The western mosquitofish (*Gambusia affinis*) is a sexually dimorphic poeciliid fish known for its worldwide biological invasion and therefore an important research model for studying invasion biology. This organism may also be used as a suitable model to explore sex chromosome evolution and reproductive development in terms of differentiation of ZW sex chromosomes, ovoviviparity, and specialization of reproductive organs. However, there is a lack of high-quality genomic data for the female *G. affinis*; hence, this study aimed to generate a chromosome-level genome assembly for it.

Results

The chromosome-level genome assembly was constructed using Oxford nanopore sequencing, BioNano, and Hi-C technology. *G. affinis* genomic DNA sequences containing 217 contigs with an N50 length of 12.9 Mb and 125 scaffolds with an N50 length of 26.5 Mb was obtained by Oxford nanopore and BioNano, respectively, and the 113 scaffolds (90.4% of scaffolds containing 97.9% nucleotide bases) were assembled into 24 chromosomes (pseudo-chromosomes) by Hi-C. The Z and W chromosomes of *G. affinis* were identified by comparative genomic analysis of female and male *G. affinis*, and the mechanism of differentiation of the Z and W chromosomes was explored. Combined with transcriptome data from six tissues, a total of 23,997 protein-coding genes were predicted and 23,737 (98.9%) genes were functionally annotated.

Conclusions

The high-quality female *G. affinis* reference genome provides a valuable omics resource for future studies of comparative genomics and functional genomics to explore the evolution of Z and W chromosomes and the reproductive developmental biology of *G. affinis*.

Keywords: *Gambusia affinis*, Nanopore sequencing, Hi-C, genome assembly, sex chromosome differentiation

Background

The western mosquitofish (*Gambusia affinis*) is a well-known invasive species of the Poeciliidae family, native to North America. To date, *G. affinis* has invaded many countries worldwide, competing successfully with native fish everywhere and destroying the ecological balance, leading to recognition by the World Conservation Union as one of the world's top 100 invasive alien species (http://www.iucngisd.org/gisd/100_worst.php)..

Although invasive western mosquitofish are harmful species with regard to the ecological environment, they are useful as model organisms in multiple life science studies, e.g., studies on behavior [1, 2], ecology [3, 4], toxicology [5, 6], and population genetics [7-9]. In addition, western mosquitofish have many interesting biological features. For example, the ZZ/ZW sex determination system in female *G. affinis* contains a W chromosome that is much longer than the Z chromosome [10], which is in contrast to the ZW chromosomes found in many birds and reptiles [11]. Additionally, in terms of reproductive development, female *G. affinis* is an oviparous fish, fertilized in the body, and developed in the body; however, without a placenta, the nutrients for the development of the fertilized egg come from yolk, and not from maternal supply. Male *G. affinis* horn fins are specialized gonopodium for *in vivo* fertilization. These biological characteristics are generally of interest for biologists, as they can provide insights into the evolution of vertebrate sex chromosomes, such as the mechanism of Z and W sex chromosome differentiation. Moreover, based on the reproductive characteristics of female *G. affinis*, this organism may serve as a model for the study of the transition from oviparity to viviparity and provide new perspectives and clues for issues, such as physiological, morphological, and immunological changes to the female reproductive tract.

Male *G. affinis* (ZZ type) scaffold level genome data have been published [12], and they serve as resources for comparative genomics among poecilids and teleosts. However, recently released data are not sufficient to explore the evolution of ZW sex chromosomes and the reproduction mode of female *G. affinis*. Further studies are necessary to overcome the lack of high-quality genomic data for female *G. affinis* (ZW type).

Accordingly, in this research, a chromosome (pseudo-chromosomes)-level genome assembly of female *G. affinis* was generated using Oxford nanopore, BioNano, and Hi-C technology. We used genomic data for male *G. affinis* and identified Z and W sex chromosomes. Combined with comparative genomic analysis of the Z and W chromosomes, the high-quality genome produced in this study is expected to provide the foundation for research on the differentiation of sex chromosomes. These data may also help explore the molecular basis of the morphological differences between the *G. affinis* females and males and the characteristics of ovoviviparous reproduction and may aid further studies on functional genomics.

Methods

Sample collection

Samples for genome sequencing of female *G. affinis* (Figure 1; sexual dimorphism is pronounced in *G. affinis*: the anal fin of adult females resembles the dorsal fins, while the anal fin of adult males is pointy and specialized for gonopodium) (Fishbase ID: 3215; NCBI taxonomy ID: 33528) were collected from the Chongde Lake at the Southwest University in Chongqing, China. The whole body (excluding the gut), brain, liver, heart, gills, gonads, and muscles were collected and quickly frozen in liquid nitrogen. Whole body samples were used for DNA sequencing, BioNano, and Hi-C for genome assembly, whereas other tissues were used for transcriptome sequencing. Animal research has been approved by the ethics committee of the Southwest University (IACUC No. Approved: IACUC-20190226-19), China.

DNA library construction and sequencing

Genomic DNA was extracted from the whole body (excluding the gut) using a Qiagen GenomicTip100 (Qiagen, Hilden, Germany). Illumina TruSeq Nano DNA Library Prep Kit (Illumina, USA) was used to construct an Illumina library with insert sizes of 350 bp, which were then sequenced on an Illumina NovaSeq platform (150-bp paired-end reads). The raw data were filtered using the following strategies: (i) filtered reads with adapters; (ii) removing reads with greater than or equal to 10% unidentified nucleotides (N); (iii) removing reads with more than 50% of bases having a phred quality of less than 5; (iv) removing reads with more than 10 nt aligned to the adapter, allowing less than or equal to 10% mismatches; and (v) removing putative polymerase chain reaction (PCR) duplicates generated by PCR amplification in the library construction process. Clean reads were used for subsequent *K-mer* analysis and nanopore data correction.

Approximately 8 μg of gDNA was prepared; Blue Pippin (Sage Science, Beverly, MA, USA) and Ligation sequencing 1D kit (SQK-LSK108; ONT, UK) were used for size selection (more than 10 kb) and nanopore library construction according to the manufacturer’s instructions. Two nanopore libraries were constructed and sequenced on two different FlowCells using the PromethION sequencer (ONT). Subreads quality control was subsequently executed on fast5 files using ONT Albacore software (v0.8.4~~),~~) [13], and the “passed filter” reads (higher quality reads) were used for subsequent analysis.

RNA library construction and sequencing

For RNA analyses, six tissues (brain, liver, heart, gills, gonads, and muscles) were extracted using an RNeasy Plus Mini Kit (Qiagen) from five individuals. The RNA purity, degradation/contamination, concentration, and integrity were measured using NanoDrop One

(Thermo Fisher Scientific, USA), 1% agarose gels, Qubit RNA Assay Kit with a Qubit 3.0 Fluorometer (Life Technologies, CA, USA), and the RNA Nano 6000 Assay Kit with a Bioanalyzer 2100 system (Agilent Technologies, CA, USA), respectively. The RNA quality criteria for the RNA samples were as follows: RNA integrity number more than 8.0 and OD 260/280 between 2.0 and 2.2. Validated RNA samples (from brain, liver, heart, gill, gonad, and muscle tissues) were used for Illumina library construction and sequencing and PacBio library preparation (pooled samples), construction, and sequencing.

For Illumina paired-end sequencing (Illumina Novaseq platform, 150-bp), the cDNA library was prepared using a TruSeq Sample Preparation Kit (Illumina). The clean data were obtained by removing reads containing adapters, reads containing poly-N, and low-quality reads from the raw data. Qualified RNA from six tissues were mixed in equal amounts, reverse-transcribed using a Clontech SMARTer PCR cDNA Synthesis Kit (TaKaRa), and subjected to PCR amplification using a PrimeSTAR GXL DNA polymerase, and the obtained 0.5–6-kb fragments were retained for PacBio sequencing library construction using a SMRTbell Template Prep Kit (Pacific Biosciences). Finally, a library for SMRT cell was sequenced using polymerase and V2.1 chemistry on a PacBio Sequel platform with 10 h of movie time.

Genomic features from K-mer analysis and nanopore assembly building

Clean reads obtained from the Illumina NovaSeq platform were applied to estimate the genome size and heterozygosity of the western mosquitofish by *K-mer* analysis (*17-mer* frequency distribution) using jellyfish ~~[13~~(v2.0; RRID:SCR_005491) [14].

Filtered Oxford nanopore sequencing data were corrected by Nextdenovo ~~(https://github.com/Nextomics/NextDenovo)~~[15] and using the following parameters: read cutoff = 3k, seed cutoff = 25k, block size = 2 g. Then, Oxford nanopore sequencing data were assembled using wtdbg ~~[14~~(v1.2.8; RRID:SCR_017225) [16]; the pipeline and parameters

were as follows: wtdbg-1.2.8 -k 0 -p 23 -S 2, wtdbg-cns -c 3 -k 15, kbm-1.2.8 -k 0 -p 21 -S 2 -O 0, wtdbg-cns -k 13 -c 3.

BWA (v0.7.12 ~~[15~~; RRID:SCR_010910) [17] and Pilon ~~[16~~(v1.21; RRID:SCR_014731) [18] were used to further improve the accuracy of the assembly, based on three rounds of mapping the Illumina reads back to the genome. Then, we used Benchmarking Universal Single-Copy Orthologs (BUSCO v3.0.1 ~~(BUSCO) [17~~; RRID:SCR_015008) [19] to evaluate the completeness of the genome assembly by searching for annotated genes in the assembly.

Genome scaffolding with BioNano auxiliary assembly

High-molecular-weight DNA was isolated from the whole body (excluding the gut) and then labeled with Labeling Master Mix and DLE-1; next, the DNA was imaged automatically with a BioNano Saphyr system. BioNano raw BNX files were *de novo* assembled into genome maps with BioNano Solve ~~(http://bnxinstall.com/solve/BionanoSolveInstall.html).~~[20]. The sorted and autodenoised single molecules were subjected to pairwise comparisons by RefAligner ~~(https://bionanogenomics.com/wp-content/uploads/2017/03/RefAligner.msi_.zip)~~[21] to identify molecule overlaps, and consensus maps were constructed. All molecules were then mapped back to the consensus maps, and the maps were recursively refined and extended (two times).

Chromosomal-level genome assembly by Hi-C

The Hi-C library was prepared following a previously described procedure [~~18~~22] with some modifications. Briefly, fresh whole-body samples (excluding the gut) were cut into 2-cm pieces and treated with 1% formaldehyde for 10 min at room temperature to induce crosslinking. The reaction was quenched by adding 2.5 M glycine to a final concentration of 0.2 M for 5 min. Nuclei were digested, marked, and ligated using 100 U *Dpn*II, biotin-14-dCTP (Invitrogen,

Carlsbad, CA, USA), and T4 DNA Ligase, respectively. After incubation overnight for reverse crosslinking, the ligated DNA was sheared into 300–600-bp fragments. The DNA fragments were blunt-end repaired and A-tailed, followed by purification through biotin-streptavidin-mediated pulldown. Finally, the Hi-C libraries were quantified and sequenced using an Illumina Hiseq platform (150-bp paired-end reads).

In total, 556 million paired-end reads were generated from the Hi-C libraries. Low-quality reads (quality scores less than 15), adaptor sequences, N ratio greater than 5% reads, and reads shorter than 30-bp were filtered out using fastp (v0.12.6~~ [19~~; RRID:SCR_016962) [23], and the clean paired-end reads (549 million paired-end reads; 81,713,239,521 bp) were then mapped to the draft assembled sequence using bowtie2 v2.3.2 [~~20~~24] to yield unique mapped paired-end reads.

As a result, 141 million uniquely mapped pair-end reads were generated, of which 76.82% were valid interaction pairs. Combined with the valid Hi-C data, we subsequently used the LACHESIS (ligating adjacent chromatin enables scaffolding *in situ*~~) [21~~; RRID:SCR_017644) [25] *de novo* assembly pipeline to produce chromosome-level sequences with the following parameters: (1) CLUSTER MIN. RE SITES = 150; (2) CLUSTER MAX. LINK DENSITY = 2.5; (3) CLUSTER NONINFORMATIVE RATIO = 1.4; (4) ORDER MIN. N RES. IN TRUNK = 60; and (5) ORDER MIN. N RES. IN SHREDS = 60. The interaction heat map of the initial assembly results of LACHESIS was drawn, according to the interaction between different scaffolds, the position and direction of the scaffolds that obviously did not meet the chromosome interaction characteristics in the figure were adjusted. Of note, if there were situations in a scaffold itself that did not meet the chromosome interaction characteristics, the scaffold was interrupted. Next, the scaffolds were adjusted separately until the overall heat map conformed to the characteristics of chromosome interaction.

We used the same method to assemble the genome of a published male *G. affinis* ~~(https://www.ncbi.nlm.nih.gov/assembly/GCA_003097735.1)~~(GCA_003097735.1) and obtained chromosomal-level genomic data.

Annotation of repetitive elements

Simple sequence repeat (SSR) sequences in the genome were analyzed by MISA ~~[22].~~(RRID:SCR_010765) [26]. For transposable elements (TEs), we first used RepeatModeler ~~(www.repeatmasker.org/RepeatModeler/),~~(v2.0.1; RRID:SCR_015027) [27], LTR_FINDER ~~[23~~(RRID:SCR_015247) [28], and MITE-Hunter software [~~24~~29], based on the principle of *de novo* methods and TE-specific architecture to build a *G. affinis* TE sequence library. The data were then combined with Repbase [~~25~~30] to construct the final database. Finally, RepeatMasker ~~software (www.repeatmasker.org)~~(v4.0.5; RRID:SCR_012954) [31] was used to predict the TEs in male and female *G. affinis* according to the final constructed TE database.

Gene prediction and function annotation

First, for homology-based prediction, the RNA-seq bam file from mapped reads to the genome by HISAT2 ~~[26~~(v2.1.0; RRID:SCR_015530) [32] and protein sequences from five sequenced vertebrates, *Danio rerio* (~~ftp://ftp.ensembl.org/pub/release-96/fasta/danio_rerio/dna/),~~GCA_000002035.4), *Oryzias latipes* (~~ftp://ftp.ensembl.org/pub/release-96/fasta/oryzias_latipes_hni/dna/),~~GCA_002234715.1), *Nothobranchius furzeri* ~~(http://nfingb.leibniz-fli.de/),~~(GCA_001465895.2), *Xiphophorus maculatus* (~~ftp://ftp.ensembl.org/pub/release-96/fasta/xiphophorus_maculatus/dna/),~~GCA_002775205.2), and *Poecilia formosa* (~~ftp://ftp.ensembl.org/pub/release-96/fasta/poecilia_formosa/dna/)~~GCA_000485575.1) were

used to predict *G. affinis* genes by GeMoMa ~~[27].~~(v2.3; RRID:SCR_017646) [33]. Second, we used Augustus (v2.5.5; RRID:SCR_008417) [34] for *ab initio* prediction, a training set generated from the GeMoMa results and transcripts of *G. affinis*, and transcripts obtained from high-throughput data using HISAT2 combined with Stringtie ~~[28].~~(v1.3.5; RRID:SCR_016323) [35]. Full-length transcriptome data were used to construct consensus sequences through clustering with IsoSeq3 ~~(https://github.com/PacificBiosciences/IsoSeq3).~~[36]. These sequences were then compared with reference genomes using GMAP ~~[29~~(RRID:SCR_008992) [37]; next, both transcripts were integrated to remove redundancy, and the results were then processed with PASA (RRID:SCR_014656) [38] to obtain the final results. Augustus' predictions were compared with the Pfam database ~~(http://pfam.sanger.ac.uk/)~~[39] to remove genes without domains, and the results of Augustus and GeMoMa were further removed by alternative splicing and integrated. Finally, the TransposonPSI ~~(http://transposonpsi.sourceforge.net/)~~[40] alignment was used to remove sequences containing transposons, yielding the final results.

Functional annotation of the predicted genes of *G. affinis* was performed by alignment to the ~~SwissProt (https://www.uniprot.org/),~~SwissPro [41], TrEMBL ~~(https://www.uniprot.org/),~~[41], Kyoto Encyclopedia of Genes and Genomes (KEGG~~; https://www.genome.jp/kegg/),~~) [42], and gene ontology (GO) [43] databases using BLAST ~~(https://blast.ncbi.nlm.nih.gov/Blast.cgi)~~(v2.3.0; RRID:SCR_004870) and KAAS (v2.1) [~~30~~44]. Motifs and domains were annotated using InterProScan (v5.24~~ [31;~~ RRID:SCR_005829) [45].

Noncoding RNA prediction

rRNAs, small nuclear RNAs (snRNAs), microRNAs (miRNAs), and tRNAs, were identified by adopting Infernal v1.1.2 [~~32~~46] using the Rfam database (release 13.0) [~~33~~47] for the *G.*

affinis genome using BLASTN (E-value ≤ 1e−5~~) [34].~~; RRID:SCR_001598) [48]. tRNAs were predicted using tRNAscan-SE (v1.3.1~~ software [35~~; RRID:SCR_010835) [49] with default parameters for eukaryotes. The rRNAs and their subunits were predicted using RNAmmer (v1.2~~ [36~~; RRID:SCR_017075) [50].

Evolutionary and comparative genomic analyses

We used OrthoMCL (version 2.0.9~~) [37~~; RRID:SCR_007839) [51] to cluster the female *G. affinis* annotated genes with an e-value cutoff of 1e–5 and Markov Chain Clustering with default inflation parameters for an all-to-all BLASTP (RRID:SCR_001010) analysis of entries for the reference genomes of 11 fishes, including *G. affinis* in this study and other 10 published fishes reported to date (*P. reticulate*, *P. formosa*, *P. latipinna*, *P. mexicana*, *X. couchianus*, *N. furzeri*, *Cyprinodon variegatus*, *Fundulus heteroclitus*, *Lepisosteus oculatus*, and *Oreochromis niloticus*). Computational Analysis of gene Family Evolution (CAFE; ~~version 4~~v4.0.1~~) [38~~; RRID:SCR_005983) [52] was used to identify expanded and contracted gene families in *G. affinis*, and these data were then used for GO and KEGG enrichment analyses.

The orthologous genes obtained from the above analyses was subjected to multiple sequence alignment using Mafft (v7.313~~) (https://mafft.cbrc.jp/alignment/software/)~~; RRID:SCR_011811) [53] and Gblocks (v0.91b ~~(http://molevol.cmima.csic.es/castresana/Gblocks.html)~~; RRID:SCR_015945) [54] to extract conserved sites based on the GTRGAMMA model and RAxML (v 8.2.11~~) [39].~~; RRID:SCR_006086) [55]. Using this tree, MCMCTREE in PAML (v4.9e~~ [40~~; RID:SCR_014932) [56] was applied to estimate the 95% confidence intervals of the differentiation times, where the published timings for the divergence of difference species were obtained with the TimeTree database ~~(http://www.timet ree.org/).~~[57].

The orthologous genes were then used in PAML codon substitution models and likelihood ratio tests (codeml) based on the branch-site model to calculate Ka and Ks, yielding positively selected genes, which were then utilized for GO and KEGG enrichment analyses.

Recognition and comparison of the W and Z chromosomes of G. affinis

Mummer ~~[41~~(v3.0; RRID:SCR_018171) [58] was used for aligning entire genomic DNA sequences from *X. couchianus*, and male and female *G. affinis*, to make the chromosome numbering system of both species the same. The W chromosome was identified by the specificity of the female *G. affinis* W chromosome length, and then, the Z chromosome in the male *G. affinis* was identified based on synteny. Mummer~~ [41]~~ was also used for aligning entire genomic DNA sequences from the Z and W chromosomes, and circos plot distributions of homologous sequence pairs among the Z and W chromosome pairs were plotted using Circos ~~[42~~(v0.69-6; RRID:SCR_011798) [59].

According to the results of the RepeatMasker analysis of female and male *G. affinis* genomes, the length and distribution of TEs on chromosomes Z and W were compared. OrthoFinder ~~[43~~(v2.3.8; RRID:SCR_017118) [60] was used to compare the genes on chromosomes Z and W.

TE insertion time analyses

We calculated female and male *G. affinis* TE insertion times in genomes using the algorithm T = K / 2r, where K is the Kimura distance-based copy divergence of TEs and r is the nucleic acid substitution rate. The K value was obtained from RepeatMasker. To estimate r values for *G. affinis*, we used LASTZ (v1.04.00~~) (http://www.bx.psu.edu/~rsharris/lastz/),~~; RRID:SCR_018556) [61] , chainNet (v2) [~~44~~62], and MULTIZ (v11.2) [~~45~~63], along with genomes used in evolutionary analyses and the female *G. affinis* genome as a reference

sequence. With the whole-genome alignments, we used the msa_view tool in the PHAST package (v1.2.1~~) [46~~; RRID:SCR_003204) [64] to extract 4D site alignments, based on the female *G. affinis* gene annotations. The phyloFit program in the PHAST package was used to estimate the phylogenetic model, with tree topology (result of evolutionary analyses) as an input parameter. The branch length results were represented as units of substitutions per site. We calculated the root-to-tip substitution rates from the most recent common ancestor of selected species to each fish lineage, and then divided the root-to-tip substitution rates by the divergence time (Divergence time: 314.47 Mya, 314.47 Million years ago) of most recent common ancestor of selected species.

Results and Discussion

Female G. affinis genome initial characteristics

A total of 30.5 Gb Illumina clean reads were used for analyzing female *G. affinis* genome size and heterozygosity using *K-mer* analysis. Based on 26,474,864,304 *17-mers* and a peak *17-mer* depth of 37 (Figure S1), the estimated heterozygosity rate was approximately 0.42%, and the estimated genome size of female *G. affinis* was about 715 Mb. Of note, the estimated genome size is similar to that of the nuclear DNA content estimated in a previous study using flow cytometry (0.75 pg, approximately 733 Mb) [~~47~~65].

De novo assembly of a female G. affinis reference genome

Next, 74.4 Gb (2,866,145 reads, average read length of 25.98 Kb, N50 35.86 Kb, and longest read length of 273 Kb) Oxford nanopore clean long reads were used to construct the reference genome. We obtained a 662-Mb genomic DNA sequences by assembly with a contig N50 length of 12.9 Mb.

The long reads assembly result consisted of 217 contigs, and the longest contig was 28.6 Mb. Then, BUSCO ~~[17]~~ was used to assess the completeness of the assembled genome. Approximately 97.2% of the complete genes could be detected in the genome of female *G. affinis*, confirming the completeness of the genome. Assembly results of long reads scaffolds obtained using optical maps were assembled with 80-Gb BioNano molecules. The final assembly contained 125 scaffolds with a scaffold N50 size of 26.4 Mb. Finally, we used the Hi-C technique to anchor the assembly scaffolds in 24 chromosomes of female *G. affinis* (Table S1). We found that 141,528,181 unique mapped paired-end reads were generated and occupied approximately 51.5% of the total clean paired-end reads (274,658,176). Then, the frequency of scaffold interactions was estimated on the basis of the pairs mapped to the scaffolds. We found that 113 scaffolds were successfully anchored in 24 chromosomes (Figure ~~1~~2, Figure S2a), consistent with the records of the chromosome number by cytogenetic analysis [10, ~~48~~66], representing 90.4% of all scaffolds and 97.9% of all scaffold nucleotide bases. The total assembly size of the chromosomes was approximately 679.4 Mb (Table 1). In the male *G. affinis* genomic DNA sequences, 734 scaffolds were successfully anchored in 24 chromosomes (Figure ~~1~~2, Figure S2b), and the total assembly size was 592.7 Mb.

Genome annotation

Assembled chromosome-level genome of female *G. affinis* were used to predict repeat sequences. In total, 5,630,271 SSRs were identified, including 5,478,552 mono-, 100,272 di-, 28,431 tri-, 19,721 tetra-, 2,048 penta-, and 1,247 hexa-nucleotide repeats. Overall, the combined the homology-based and *de novo* prediction results indicated that TEs accounted for 22.54% of the assembly genome (Table S2). Additionally, class I TEs (RNA transposons) occupied approximately 5.15% of the assembly genome. The most abundant RNA transposons found in the *G. affinis* assembly genome were long interspersed nuclear elements, which

constituted 54.37% of all identified RNA transposons. The female *G. affinis* genome was very rich in class II TEs (DNA transposons), which occupied almost 11.83% of genome content.

For genome annotation, 23,997 protein-coding genes were predicted in the female *G. affinis* genome. Compared with other existing published poeciliid fish annotated information, a number of genes in female *G. affinis* was similar to those in *P. formosa* (23,615 genes) and *X. maculatus* (23,628 genes) (Table S3 and Figure S3). BUSCO gene prediction was carried out using the actinopterygii_odb9 single-copy homologous gene to predict the existing sequence of the genome. Approximately 97% of complete gene components could be found in this gene set, indicating that most of the conserved genes were well predicted and that the prediction results were relatively reliable (Table S4). Finally, 23,737 genes were annotated in at least one of the databases (KOG, KEGG, NR, SwissProt, GO), and up to 98.92% of *G. affinis* genes were functionally annotated (Table S5). Finally, 143 snRNAs, 220 rRNAs, 371 miRNAs, and 3885 tRNAs were also identified.

Genome evolution

In order to determine the evolutionary relationships between *G. affinis* and other vertebrates, a phylogenetic tree was reconstructed on the basis of 6,457 single-copy orthologous genes from 10 other vertebrate genomes (Figure ~~2a~~3a). *L. oculatus* and *O. niloticus* were used as outgroups. As a species of the family Poeciliidae, *G. affinis* clustered into one branch with other fish from Poeciliidae. Compared with six other sequenced members of the Poeciliidae family, *G. affinis* had a closer relationship with *X. couchianus*, consistent with previously published phylogenies [~~49~~67]. Next, a timetree was created based on the above 6,457 single-copy orthologous genes, and the estimated divergence time between *G. affinis* and *X. couchianus* was found to be approximately 16.57 million years ago (Mya; Figure ~~2~~3; Figure

S3). In addition, the divergence time between *G. affinis* and four other members of the Poeciliidae family was approximately 22.75 Mya.

In order to examine the evolutionary history of gene families, we performed gene family expansion and contraction analysis with the female *G. affinis* genes. We found 652 expansion gene families and 1,046 contraction gene families (Figure ~~2b~~3b). Expansion gene families were enriched in 44 GO (Table S6) categories and 34 KEGG pathways (Table S7), most of which were related to oxygen metabolism, olfactory pathways, and visual pathways. Next, codeml was used to calculate the average Ka/Ks values and conduct branch-site likelihood ratio analyses to detect positively selected genes in the female *G. affinis* genome. The results showed that there were 590 positively selected genes in the female *G. affinis* genome. The positively selected genes were enriched in 12 GO categories (aspartic-type endopeptidase activity, DNA repair, microtubule binding, insulin-like growth factor binding, tRNA aminoacylation for protein translation, microtubule motor activity, rRNA processing, microtubule-based movement, protein dephosphorylation, protein tyrosine phosphatase activity, chromatin binding, and nucleus) and three KEGG pathways (complement and coagulation cascades, peroxisome, and platelet activation).

Recognition and evolution of sex chromosomes

Genetic controlled sex determination systems in fish are variable, ranging from XX/XY to ZZ/ZW [~~50~~68]. Fish generally do not have highly morphologically differentiated sex chromosomes, making it difficult to distinguish between autosomes and sex chromosomes. Hence, there are only a few fish species for which there is known information on sex determination mechanisms and sex chromosome systems. Therefore, a suitable experimental model is required for the identification and elucidation of the mechanisms of fish sex chromosome evolution, and the female *G. affinis* is a suitable and consistent model.

Early karyotype analysis demonstrated that female *G. affinis* shows heterogamy of the ZW type, and its W chromosome is much longer than other chromosomes [10, ~~48~~66]. The longest sequence was selected from the assembly results at the chromosome level as the W candidate chromosome, and one female-specific DNA marker [~~51~~69] was used for confirmation. In the end, the marker has been aligned to the W candidate chromosome, but was not found in the genomic DNA sequences of the male *G. affinis*. Analysis of the synteny of the whole genomes of female and male mosquitofish by Mummer ~~[41]~~ demonstrated that the Z chromosome was also present in the male *G. affinis* genomic DNA sequences (Figure ~~1~~2). By comparing the Z and W chromosomes (Figure ~~3a~~4a), we found the length of the W and Z chromosome repeat sequences to be approximately 5.0 Mb and 8.5 Mb, respectively. Among them, the length and content of the *Helitron* superfamily of the two chromosomes (Z: 591,639 bp, 1.3%; W: 6,6868 bp, 0.24%) were significantly different (Results have been submitted to GigaDB). There were 1279 and 1027 genes on the W and Z chromosomes, respectively. Homologous analysis showed that there were 712 one-to-one pairs. There were 118 and 203 genes on the W and Z chromosomes unassigned to any gene groups, and the others were of the one-to-many and many-to-many types; these results provide research directions for our future analyses on functional genomics (Results have been submitted to GigaDB).

Some researchers have studied the role of transposons in sex chromosome differentiation, and they found that TEs seem to be play an important role in the evolution of sex chromosomes, with their accumulation and loss having huge effects on the lengths of sex chromosomes [~~52-54~~70-72]. However, differences in TE contents between Z and W chromosomes alone cannot determine the true course of differentiation, e.g., whether the increased length of the W chromosome compared with that of the Z chromosome is caused by extension of the W chromosome or by degeneration of the Z chromosome. There is no substantial evidence to explain this observation. Therefore, the introduction of the time factor is extremely important.

G. holbrooki and *G. affinis* are so closely related that for a long time, biologists thought they were the same species. Phylogenetic analyses estimated that their divergence time was approximately 2–7 Mya [~~51, 55, 56~~69, 73, 74], and other researchers showed that the XY and ZW sex determination mechanisms had independent origins in *G. holbrooki* and *G. affinis*, respectively [~~50~~68]. Therefore, we speculate that the differentiation of Z and W sex chromosomes is a very recent event. Additionally, previous studies suggested that this process may be enriched on the W chromosome by TEs, leading to an increase in the sex chromosome size during the early phase of differentiation and the subsequent reduction in size later during evolution [~~57~~75]. If this hypothesis is correct, then we should be able to observe a large number of transposons inserted in the W chromosome in the recent past (between 2–7 Mya). Indeed, our results indicated very recent mass insertion events of TEs into the W chromosome (Figure ~~3b~~4b), and the insertion time characteristics of the TEs into the W chromosome were specific because its insertion time trends were dramatically different from those of autosomal and Z-chromosomal TEs (Figure ~~3c~~4c and Figure S5). Moreover, we speculate that most of the long gaps (Figure ~~3a~~4a) on the W chromosome were also caused by the aggregation of too many highly similar TE sequences to form TE clusters through the recent activation of TEs. Thus, we expected that the TE content of the W chromosome of *G. affinis* should be much higher than that observed till date. Accordingly, our results showed that the cause of sex chromosome differentiation in female *G. affinis* was likely to be related to extension of the W chromosome.

Conclusions

In this study, we assembled the chromosome-level female western mosquitofish genome using the most mainstream technology available. In terms of parameters such as contig N50, scaffold N50, and gene annotation number, these are high-quality genomic data. Evolutionary analysis provides ideas for future work, for example, oxygen transport in mosquitofish deserves

attention. We conducted a preliminary study on W and Z sex chromosome differentiation based on the specificity of the sex chromosome in female western mosquitofish and provided data to support the previous hypothesis that a longer W chromosome is associated with the activity (insertion) of TEs. In conclusion, our high-quality genomic data lay the foundation for the study of chromosome evolution, reproductive characteristics, and sexual dimorphism in western mosquitofish.

Availability of supporting data

The raw genome and RNA sequencing data were deposited in the SRA under Bioproject number PRJNA599452. The chromosome-level genome, annotation, and other supporting data are also available via the GigaScience database, *GigaDB* [~~58~~76].

Abbreviations

BLAST: Basic Local Alignment Search Tool; bp: base pairs; BUSCO: Benchmarking Universal Single-Copy Orthologs; BWA: Burrows-Wheeler Aligner; CAFE: Computational Analysis of gene Family Evolution ; GeMoMa: Gene Model Mapper; Gb: gigabase pairs; GO: Gene Ontology; Hi-C: High-throughput chromosome conformation capture; KASS: KEGG Automatic Annotation Server; kb: kilobase pairs; KEGG: Kyoto Encyclopedia of Genes and Genomes; Mb: megabase pairs; MRCA: most recent common ancestor; LTR: long terminal repeat; NCBI: National Center for Biotechnology Information; PAML: Phylogenetic Analysis by Maximum Likelihood; PASA: Program to Assemble Spliced Alignments; RAxML: Randomized Accelerated Maximum Likelihood; RNA-seq: RNA sequencing; TE: transposable element.

Competing Interests

The authors declare that there have no competing interests.

Author Contributions

F.S. performed the major part of data analysis and drafted the manuscript. Y.M. and N.L. contributed to samples collection and drafted the manuscript. Z.P. and L.A. contributed to research design and final edits to the manuscript. All authors read and approved the final manuscript.

Acknowledgements

We are grateful to Drs. Aurélie Kapusta, Guojie Zhang, and Cai Li for help with estimating substitution rate and transposon insertion time. We ~~also~~ thank Drs. Wen Wang, Lei Chen, Kun Wang, Ru Zhang, Botong Zhou, Zeshan Lin, and Bao Wang for discussion. We also thank Tian Lu for his help with preparing the fish image. This work was supported by the grants from the National Key Research and Development Program of China (2018YFD0900805), the National Natural Science Foundation of China (31872204), the Fundamental Research Funds for the Central Universities (XDJK2020C043), and the Natural Science Foundation of Chongqing Postdoctoral Science Foundation (cstc2019jcyj-bshX0071).

References

1. Russo G, Chou A, Rettig JE and Smith GR. Foraging responses of mosquitofish (*Gambusia affinis*) to items of different sizes and colors. J Freshw Ecol. 2008;23 4:677-8. doi:10.1080/02705060.2008.9664256.
2. Cote J, Fogarty S, Weinersmith K, Brodin T and Sih A. Personality traits and dispersal tendency in the invasive mosquitofish (*Gambusia affinis*). Proc Biol Sci. 2010;277 1687:1571-9. doi:10.1098/rspb.2009.2128.
3. Smith GR and Smith LE. Effects of Western Mosquitofish (*Gambusia affinis*) on tadpole production of Gray Treefrogs (*Hyla versicolor*). Herpetol Conserv Biol. 2015;10 2:723-7.
4. Merkley SS, Rader RB and Schaalje GB. Introduced Western Mosquitofish (*Gambusia affinis*) reduce the emergence of aquatic insects in a desert spring. Freshw Sci. 2015;34 2:564-73. doi:10.1086/680381.
5. Raut SA and Angus RA. Triclosan has endocrine ‐ disrupting effects in male western mosquitofish, *Gambusia affinis*. Environ Toxicol Chem. 2010;29 6:1287-91. doi:10.1002/etc.150.
6. Brockmeier EK, Ogino Y, Iguchi T, Barber DS and Denslow ND. Effects of 17beta-trenbolone on Eastern and Western mosquitofish (*Gambusia holbrooki* and *G. affinis*) anal fin growth and gene expression patterns. Aquat Toxicol. 2013;128-129:163-70. doi:10.1016/j.aquatox.2012.12.007.
7. Vera M, Díez ‐ del ‐ Molino D and García ‐ Marín JL. Genomic survey provides insights into the evolutionary changes that occurred during European expansion of the invasive mosquitofish (*Gambusia holbrooki*). Mol Ecol. 2016;25 5:1089-105. doi:10.1111/mec.13545.

8. Diez-del-Molino D, Carmona-Catot G, Araguas RM, Vidal O, Sanz N, Garcia-Berthou E, et al. Gene flow and maintenance of genetic diversity in invasive mosquitofish (*Gambusia holbrooki*). PLoS ONE. 2013;8 12:e82501. doi:10.1371/journal.pone.0082501.

9. Vidal O, Sanz N, Araguas RM, Fernández‐Cebrian R, Diez‐del‐Molino D and García‐Marín JL. SNP diversity in introduced populations of the invasive *Gambusia holbrooki*. Ecol Freshw Fish. 2012;21 1:100-8. doi:10.1111/j.1600- 0633.2011.00527.x.

10. Black DA and Howell WM. The North American mosquitofish, *Gambusia affinis*: a unique case in sex chromosome evolution. Copeia. 1979; 1979:509-13. doi:10.2307/1443231.

11. Irwin DE. Sex chromosomes and speciation in birds and other ZW systems. Mol Ecol. 2018;27 19:3831-51. doi:10.1111/mec.14537.

12. Hoffberg SL, Troendle NJ, Glenn TC, Mahmud O, Louha S, Chalopin D, et al. A High-Quality Reference Genome for the Invasive Mosquitofish *Gambusia affinis* Using a Chicago Library. G3-Genes Genom Genet. 2018;8 6:1855-61. doi:10.1534/g3.118.200101.

~~13~~13. Albacore. https://community.nanoporetech.com/protocols/albacore-offline-basecalli/v/abec_2003_v1_revan_29nov2016/linux. Accessed May 27, 2019.

14. Marcais G and Kingsford C. A fast, lock-free approach for efficient parallel counting of occurrences of *k-mers*. Bioinformatics. 2011;27 6:764-70. doi:10.1093/bioinformatics/btr011.

~~14~~15. NextDenovo. https://github.com/Nextomics/NextDenovo/. Accessed July 21, 2019.

16. Ruan J and Li H. Fast and accurate long-read assembly with wtdbg2. Nat Methods. 2019; 17 2:155-8. doi:10.1038/s41592-019-0669-3.

~~15~~17. Li H and Durbin R. Fast and accurate short read alignment with Burrows-Wheeler transform. Bioinformatics. 2009;25 14:1754-60. doi:10.1093/bioinformatics/btp324.

~~16~~18. Walker BJ, Abeel T, Shea T, Priest M, Abouelliel A, Sakthikumar S, et al. Pilon: an integrated tool for comprehensive microbial variant detection and genome assembly improvement. PLoS ONE. 2014;9 11:e112963. doi:10.1371/journal.pone.0112963.

~~17~~19. Simao FA, Waterhouse RM, Ioannidis P, Kriventseva EV and Zdobnov EM. BUSCO: assessing genome assembly and annotation completeness with single-copy orthologs. Bioinformatics. 2015;31 19:3210-2. doi:10.1093/bioinformatics/btv351.

~~18~~20. BioNano Solve. http://bnxinstall.com/solve/BionanoSolveInstall.html. Accessed August 21, 2019.

21. RefAligner. https://bionanogenomics.com/wp-content/uploads/2017/03/RefAligner.msi_.zip. Accessed August 21, 2019.

22. Belton JM, McCord RP, Gibcus JH, Naumova N, Zhan Y and Dekker J. Hi-C: a comprehensive technique to capture the conformation of genomes. Methods. 2012;58 3:268-76. doi:10.1016/j.ymeth.2012.05.001.

~~19~~23. Chen S, Zhou Y, Chen Y and Gu J. fastp: an ultra-fast all-in-one FASTQ preprocessor. Bioinformatics. 2018;34 17:i884-i90. doi:10.1093/bioinformatics/bty560.

~~20~~24. Langmead B and Salzberg SL. Fast gapped-read alignment with Bowtie 2. Nat Methods. 2012;9 4:357-9. doi:10.1038/nmeth.1923.

~~21~~25. Burton JN, Adey A, Patwardhan RP, Qiu R, Kitzman JO and Shendure J. Chromosome-scale scaffolding of de novo genome assemblies based on chromatin interactions. Nat Biotechnol. 2013;31 12:1119-25. doi:10.1038/nbt.2727.

~~22~~26. Beier S, Thiel T, Munch T, Scholz U and Mascher M. MISA-web: a web server for microsatellite prediction. Bioinformatics. 2017;33 16:2583-5. doi:10.1093/bioinformatics/btx198.

~~23~~27. RepeatModeler. http://www.repeatmasker.org/RepeatModeler. Accessed July 27, 2019.

28. Xu Z and Wang H. LTR_FINDER: an efficient tool for the prediction of full-length LTR retrotransposons. Nucleic Acids Res. 2007;35:W265-8. doi:10.1093/nar/gkm286.

~~24~~29. Han Y and Wessler SR. MITE-Hunter: a program for discovering miniature inverted-repeat transposable elements from genomic sequences. Nucleic Acids Res. 2010;38 22:e199. doi:10.1093/nar/gkq862.

~~25~~30. Bao W, Kojima KK and Kohany O. Repbase Update, a database of repetitive elements in eukaryotic genomes. Mob DNA. 2015;6:11. doi:10.1186/s13100-015-0041-9.

~~26~~31. RepeatMasker. http://www.repeatmasker.org/RMDownload.html. Accessed July 27, 2019.

32. Kim D, Langmead B and Salzberg SL. HISAT: a fast spliced aligner with low memory requirements. Nat Methods. 2015;12 4:357-60. doi:10.1038/nmeth.3317.

~~27~~33. Keilwagen J, Wenk M, Erickson JL, Schattat MH, Grau J and Hartung F. Using intron position conservation for homology-based gene prediction. Nucleic Acids Res. 2016;44 9:e89. doi:10.1093/nar/gkw092.

~~28~~34. Stanke M, Waack S. Gene prediction with a hidden Markov model and a new intron submodel. Bioinformatics. 2003;19(suppl 2):215-25. doi:10.1093/bioinformatics/btg1080.

35. Pertea M, Pertea GM, Antonescu CM, Chang TC, Mendell JT and Salzberg SL. StringTie enables improved reconstruction of a transcriptome from RNA-seq reads. Nat Biotechnol. 2015;33 3:290-5. doi:10.1038/nbt.3122.

~~29~~36. IsoSeq3. https://github.com/PacificBiosciences/IsoSeq3. Accessed July 30, 2019.

37. Wu TD and Watanabe CK. GMAP: a genomic mapping and alignment program for mRNA and EST sequences. Bioinformatics. 2005;21 9:1859-75. doi:10.1093/bioinformatics/bti310.

~~30~~38. Haas BJ, Delcher AL, Mount SM, Wortman JR, Smith RK Jr, Hannick LI, et al. Improving the Arabidopsis genome annotation using maximal transcript alignment assemblies. Nucleic Acids Res. 2003;31 19:5654-66. doi: 10.1093/nar/gkg770.

39. Finn RD, Bateman A, Clements J, Coggill P, Eberhardt RY, Eddy SR, et al. Pfam: the protein families database. Nucleic Acids Res. 2014;42:D222-30. doi: 10.1093/nar/gkt1223.

40. TransposonPSI. http://transposonpsi.sourceforge.net/. Accessed August 30, 2019.

41. Apweiler R, Bairoch A, Wu CH, et al. UniProt: the universal protein knowledgebase. Nucleic Acids Res 2017;45:D158-69. doi:10.1093/nar/gkw1099.

42. The Gene Ontology Consortium. Expansion of the Gene Ontology knowledgebase and resources. Nucleic Acids Res 2017;45:D331-8. doi:10.1093/nar/gkw1108.

43. Kanehisa M, Goto S, Sato Y, et al. Data, information, knowledge and principle: back to metabolism in KEGG. Nucleic Acids Res 2014;42:D199-205. doi:10.1093/nar/gkt1076.

44. Moriya Y, Itoh M, Okuda S, Yoshizawa AC and Kanehisa M. KAAS: an automatic genome annotation and pathway reconstruction server. Nucleic Acids Res. 2007;35:W182-5. doi:10.1093/nar/gkm321.

~~31~~45. Jones P, Binns D, Chang HY, Fraser M, Li W, McAnulla C, et al. InterProScan 5: genome-scale protein function classification. Bioinformatics. 2014;30 9:1236-40. doi:10.1093/bioinformatics/btu031.

~~32~~46. Nawrocki EP and Eddy SR. Infernal 1.1: 100-fold faster RNA homology searches. Bioinformatics. 2013;29 22:2933-5. doi:10.1093/bioinformatics/btt509.

~~33~~47. Kalvari I, Argasinska J, Quinones-Olvera N, Nawrocki EP, Rivas E, Eddy SR, et al. Rfam 13.0: shifting to a genome-centric resource for non-coding RNA families. Nucleic Acids Res. 2018;46 D1:D335-42. doi:10.1093/nar/gkx1038.

~~34~~48. Camacho C, Coulouris G, Avagyan V, Ma N, Papadopoulos J, Bealer K, et al. BLAST+: architecture and applications. BMC Bioinformatics. 2009;10:421. doi:10.1186/1471-2105-10-421.

~~35~~49. Lowe TM and Eddy SR. tRNAscan-SE: a program for improved detection of transfer RNA genes in genomic sequence. Nucleic Acids Res. 1997;25 5:955-64. doi:10.1093/nar/25.5.955.

~~36~~50. Lagesen K, Hallin P, Rodland EA, Staerfeldt HH, Rognes T and Ussery DW. RNAmmer: consistent and rapid annotation of ribosomal RNA genes. Nucleic Acids Res. 2007;35 9:3100-8. doi:10.1093/nar/gkm160.

~~37~~51. Li L, Stoeckert CJ, Jr. and Roos DS. OrthoMCL: identification of ortholog groups for eukaryotic genomes. Genome Res. 2003;13 9:2178-89. doi:10.1101/gr.1224503.

~~38~~52. De Bie T, Cristianini N, Demuth JP and Hahn MW. CAFE: a computational tool for the study of gene family evolution. Bioinformatics. 2006;22 10:1269-71. doi:10.1093/bioinformatics/btl097.

~~39~~53. Yamada KD, Tomii K, Katoh K. Application of the MAFFT sequence alignment program to large data-reexamination of the usefulness of chained guide trees. Bioinformatics. 2016;32 21:3246-51. doi: 10.1093/bioinformatics/btw412.

54. Gblocks. http://molevol.cmima.csic.es/castresana/Gblocks.html. Accessed September 5, 2019.

55. Stamatakis A. RAxML version 8: a tool for phylogenetic analysis and post-analysis of large phylogenies. Bioinformatics. 2014;30 9:1312-3. doi:10.1093/bioinformatics/btu033.

~~40~~56. Yang Z. PAML: a program package for phylogenetic analysis by maximum likelihood. Comput Appl Biosci. 1997;13 5:555-6. doi:10.1093/bioinformatics/13.5.555.

~~41~~57. TIMETREE. http://www.timetree.org. Accessed September 10, 2019.

58. Kurtz S, Phillippy A, Delcher AL, Smoot M, Shumway M, Antonescu C, et al. Versatile and open software for comparing large genomes. Genome Biol. 2004;5 2:R12. doi:10.1186/gb-2004-5-2-r12.

~~42~~59. Krzywinski M, Schein J, Birol I, Connors J, Gascoyne R, Horsman D, et al. Circos: an information aesthetic for comparative genomics. Genome Res. 2009;19 9:1639-45. doi:10.1101/gr.092759.109.

~~43~~60. Emms DM and Kelly S. OrthoFinder: phylogenetic orthology inference for comparative genomics. Genome Biol. 2019;20 1:238. doi:10.1186/s13059-019-1832-y.

~~44~~61. Kent WJ, Baertsch R, Hinrichs A, Miller W and Haussler D. Evolution's cauldron: duplication, deletion, and rearrangement in the mouse and human genomes. Proc Natl Acad Sci U S A. 2003;100 20:11484-9. doi:10.1073/pnas.1932072100.

~~45~~62. LASTZ. http://www.bx.psu.edu/~rsharris/lastz/. Accessed September 20, 2019.

63. Blanchette M, Kent WJ, Riemer C, Elnitski L, Smit AF, Roskin KM, et al. Aligning multiple genomic sequences with the threaded blockset aligner. Genome Res. 2004;14 4:708-15. doi:10.1101/gr.1933104.

~~46~~64. Hubisz MJ, Pollard KS and Siepel A. PHAST and RPHAST: phylogenetic analysis with space/time models. Brief Bioinform. 2011;12 1:41-51. doi:10.1093/bib/bbq072.

~~47~~65. Tiersch TR, Chandler RW, Wachtel SS and Elias S. Reference-Standards for Flow-Cytometry and Application in Comparative Studies of Nuclear-DNA Content. Cytometry. 1989;10 6:706-10. doi:10.1002/cyto.990100606.

~~48~~66. Chen T and Ebeling A. Karyological evidence of female heterogamety in the mosquitofish, *Gambusia affinis*. Copeia. 1968; 1968:70-5. doi:10.2307/1441552.

~~49~~67. Furness AI, Pollux BJA, Meredith RW, Springer MS and Reznick DN. How conflict shapes evolution in poeciliid fishes. Nat Commun. 2019;10 1:3335. doi:10.1038/s41467-019-11307-5.

~~50~~68. Kottler VA, Feron R, Nanda I, Klopp C, Du K, Kneitz S, et al. Independent Origin of XY and ZW Sex Determination Mechanisms in Mosquitofish Sister Species. Genetics. 2020;214 1:193-209; doi:10.1534/genetics.119.302698.

~~51~~69. Lamatsch DK, Adolfsson S, Senior AM, Christiansen G, Pichler M, Ozaki Y, et al. A transcriptome derived female-specific marker from the invasive Western mosquitofish (*Gambusia affinis*). PLoS ONE. 2015;10 2:e0118214. doi:10.1371/journal.pone.0118214.

~~52~~70. Rosolen LAM, Vicari MR and Almeida MC. Accumulation of Transposable Elements in Autosomes and Giant Sex Chromosomes of *Omophoita* (Chrysomelidae: Alticinae). Cytogenet Genome Res. 2018;156 4:215-22. doi:10.1159/000495199.

~~53~~71. Erlandsson R, Wilson JF and Paabo S. Sex chromosomal transposable element accumulation and male-driven substitutional evolution in humans. Mol Biol Evol. 2000;17 5:804-12. doi:10.1093/oxfordjournals.molbev.a026359.

~~54~~72. Dechaud C, Volff JN, Schartl M and Naville M. Sex and the TEs: transposable elements in sexual development and function in animals. Mob DNA. 2019;10:42. doi:10.1186/s13100-019-0185-0.

~~55~~73. Lydeard C, Wooten MC and Meyer A. Molecules, morphology, and area cladograms: a cladistic and biogeographic analysis of *Gambusia* (Teleostei: Poeciliidae). Syst Biol. 1995;44 2:221-36. doi:10.1093/sysbio/44.2.221.

~~56~~74. Helmstetter AJ, Papadopulos AS, Igea J, Van Dooren TJ, Leroi AM and Savolainen V. Viviparity stimulates diversification in an order of fish. Nat Commun. 2016;7:11271. doi:10.1038/ncomms11271.

~~57~~75. Schartl M, Schmid M and Nanda I. Dynamics of vertebrate sex chromosome evolution: from equal size to giants and dwarfs. Chromosoma. 2016;125 3:553-71. doi:10.1007/s00412-015-0569-y.

5876. Shao F, Ludwig A, Mao Y, Liu N and Peng Z. Supporting data for "Chromosome-level genome assembly of the female western mosquitofish (*Gambusia affinis*)". GigaScience Database. 2020. http://doi.org/10.5524/100778

Table 1. Genome assembly statistics of *Gambusia affinis*.

	Nanopore	BioNano	Hi-C (♀)[#]	Hi-C (♂)[##]
Total assembly size of contig/scaffold/chromosome (bp)	662,579,534	680,140,492	679,423,294[*]	592,666,412[*]
Number of contig/scaffold/chromosome	217	125	24	24
N50 contig/scaffold/chromosome length (bp)	12,906,370	26,455,434	29,761,488	25,946,590
N90 contig/scaffold/chromosome length (bp)	1,629,223	18,394,109	23,709,503	21,272,223
Longest contig/scaffold/chromosome (bp)	28,665,999	31,542,956	45,125,082	30,583,032

* The length of 24 chromosomes, excluding the length of the unanchored sequences.

[#] Based on the data generated in this study.

[##] Based on published data [12].

Figure Legends

Figure 1. Image of female (top) and male (bottom) *G. affinis*.

Figure 2. Genomic synteny of *X. couchianus*, female *G. affinis*, and male *G. affinis*. Female *G. affinis* LG01 represents the W chromosome and male *G. affinis* LG01 represents the Z chromosome.

Figure 23. Phylogenetic and evolutionary analysis of *G. affinis*. (a) Divergence time estimates and gene clusters in *G. affinis* and other species. (b) Expansion and contraction of *G. affinis* gene families. MRCA represents most recent common ancestor, and circle diagrams and numbers below represent the proportion and specific values of the gene families of expansion and contraction, respectively (red represents contraction, green represents expansion).

Figure 34. Comparative genomic analysis of the Z and W chromosomes. (a) Circos plot of Z and W chromosome alignment; the red region represents the repeat sequence density, and the green region represents the GC density. (b) Distribution of the transposon activity time for the W chromosome. (c) Distribution of transposon activity time for the Z chromosome.

Additional files

Table S1: Result of female and male *Gambusia affinis* genomic assembly at chromosome-level.

Table S2: Transposable elements (TEs) annotation in the female *Gambusia affinis* genome.

Table S3: Comparative analysis of the annotated gene set of female *Gambusia affinis* with those of five teleosts.

Table S4: Assessment of female *Gambusia affinis* genome completeness by BUSCO.

Table S5: Statistics for gene function annotation in female *Gambusia affinis* genome.

Table S6: Expansion gene families of female *Gambusia affinis* were enriched in 44 GO categories.

Table S7: Expansion gene families of female *Gambusia affinis* were enriched in 34 KEGG pathways.

Figure S1: Frequency distribution of the 17-mer graph analysis used to estimate the size of female *Gambusia affinis*.

Figure S2: Western mosquitofish genome scaffold contact matrix using Hi-C data. (a) Female western mosquitofish. (b) Male western mosquitofish. The color bar indicates the contact density from red (high) to white (low).

Figure S3: The comparisons of CDS length, exon length, exon-number, gene length, intro length, and intron number in the genomes of female *Gambusia affinis* and other teleosts.

Figure S4: Divergence time of *Gambusia affinis* and other fish species.

Figure S5: Distribution of transposon activity time for different autosomes of female *Gambusia affinis*.

Additional File: All genes located on the Z and W sex chromosomes with their locations.

Figure

Click here to access/download;Figure;Figure_1.JPG

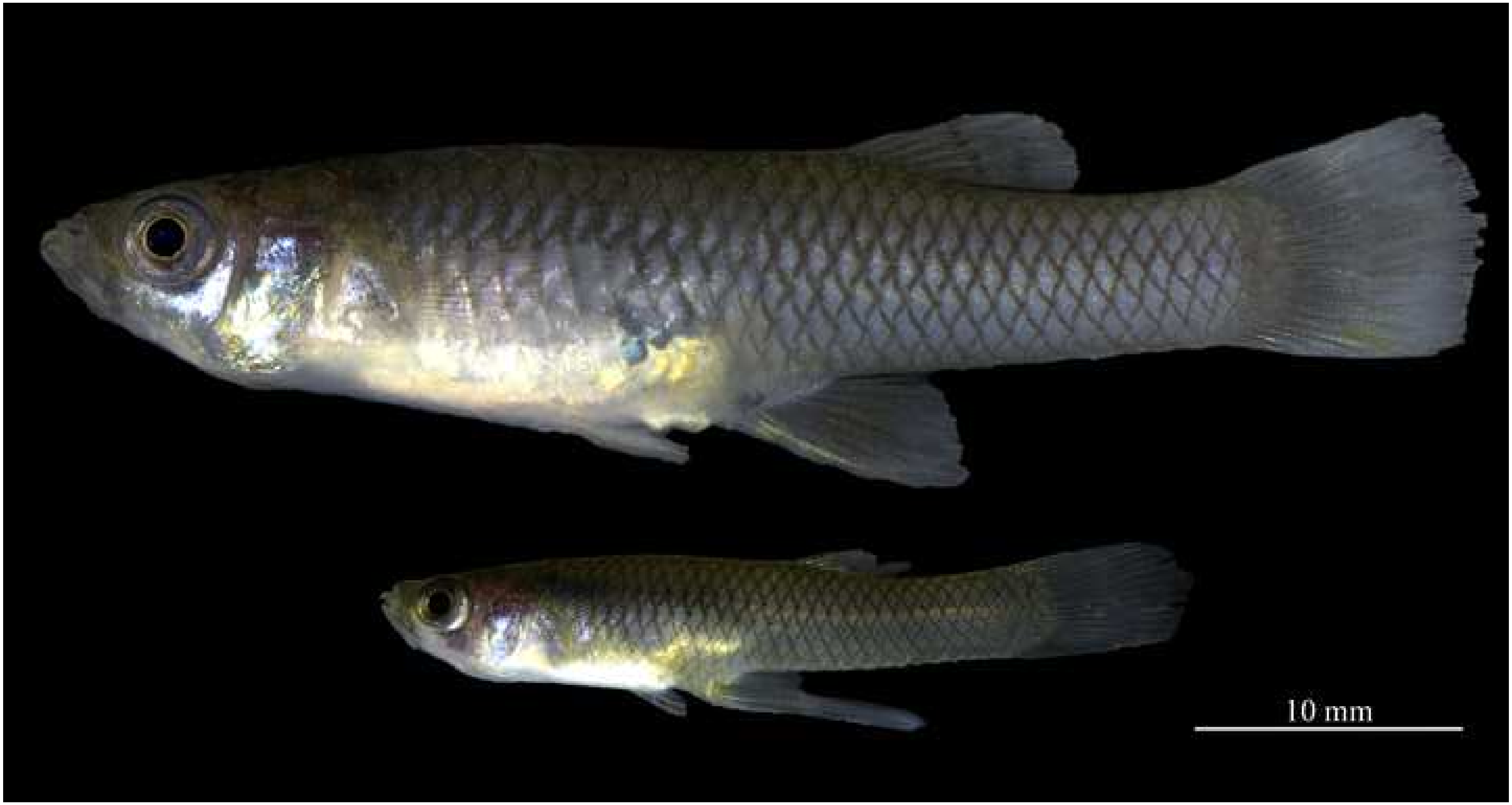

Figure

Click here to access/download;Figure;Figure_2.png

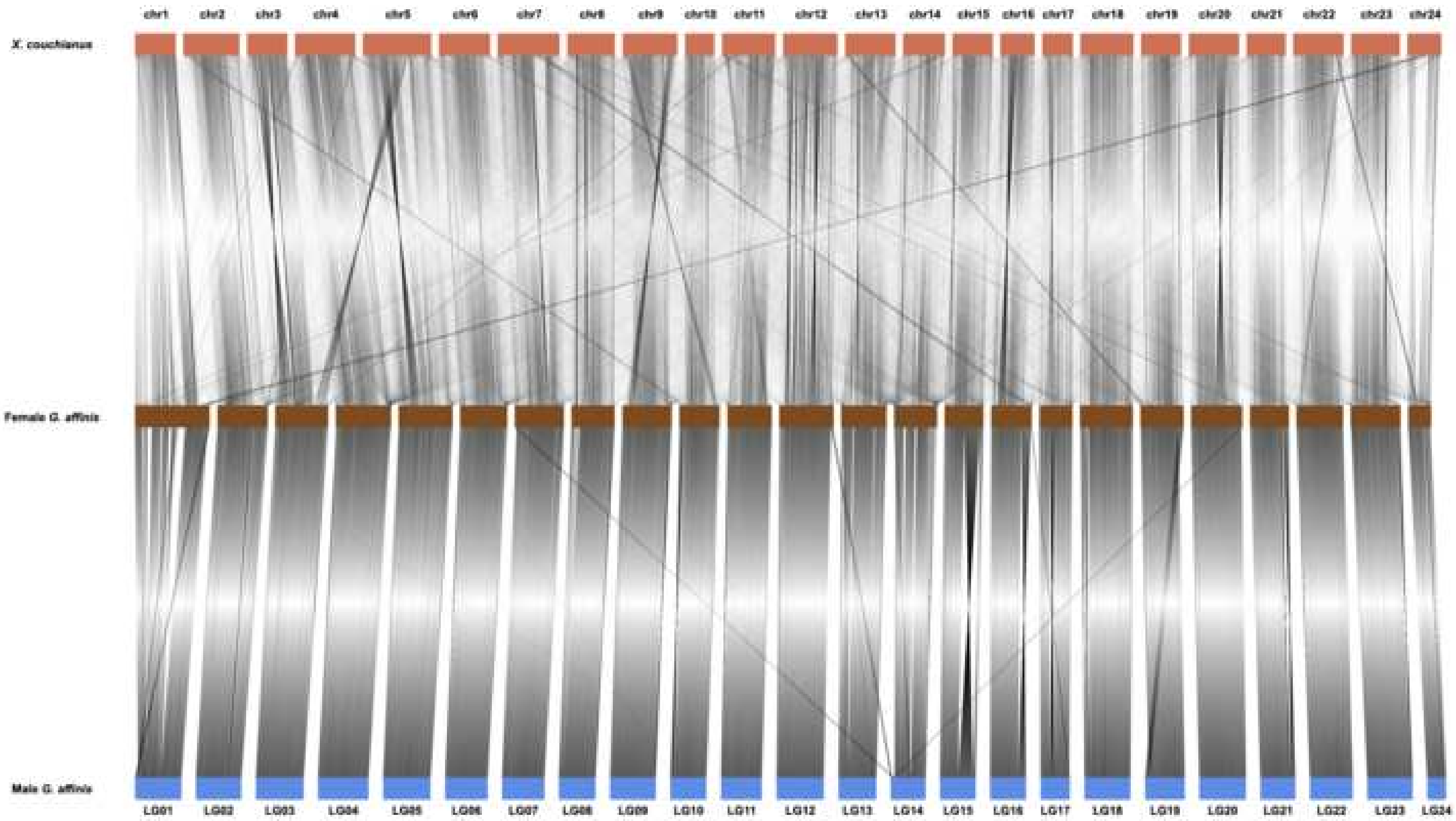

Figure
Click here to access/download;Figure;Figure_3.pdf

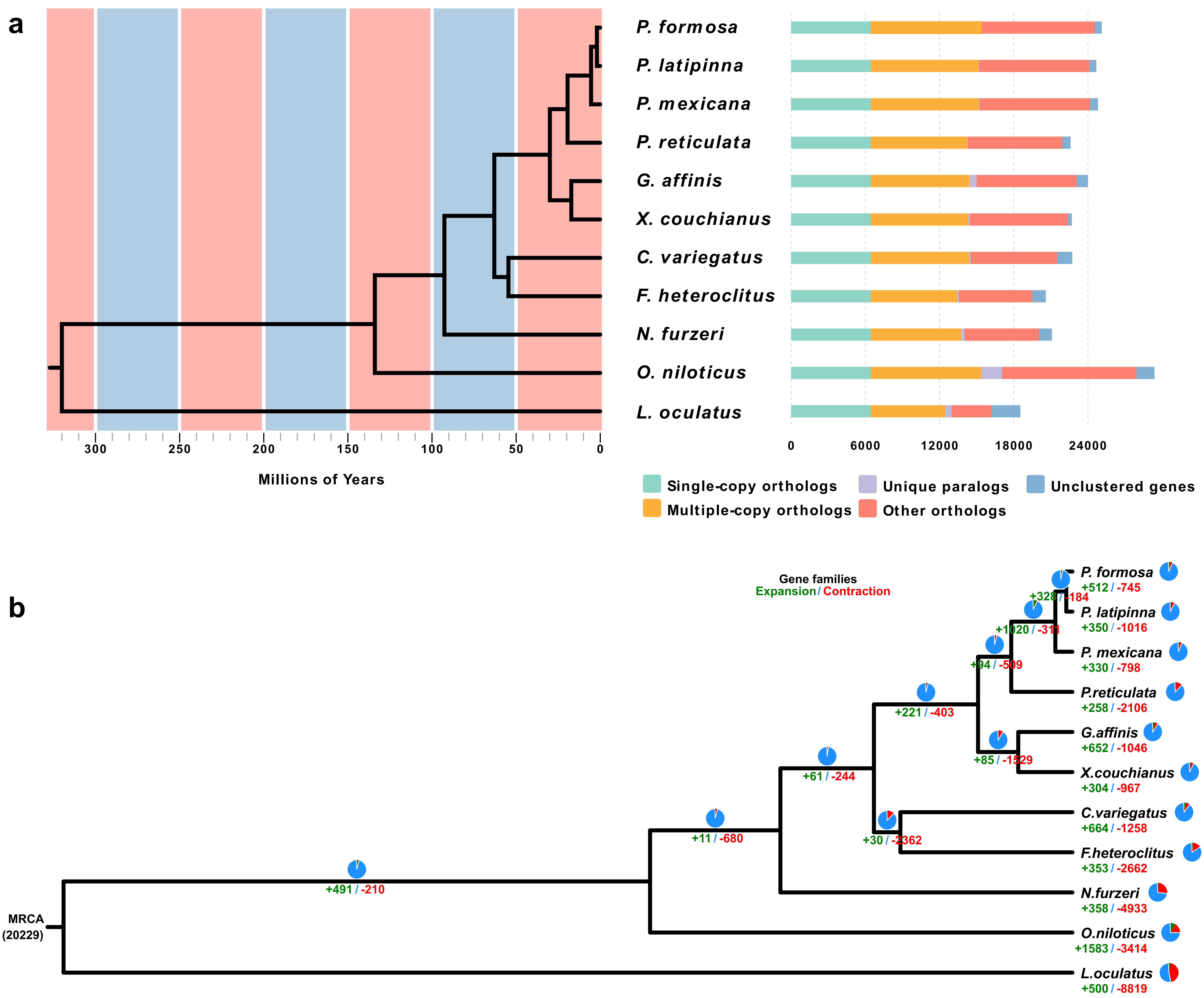

Figure
Click here to access/download;Figure;Figure_4.pdf

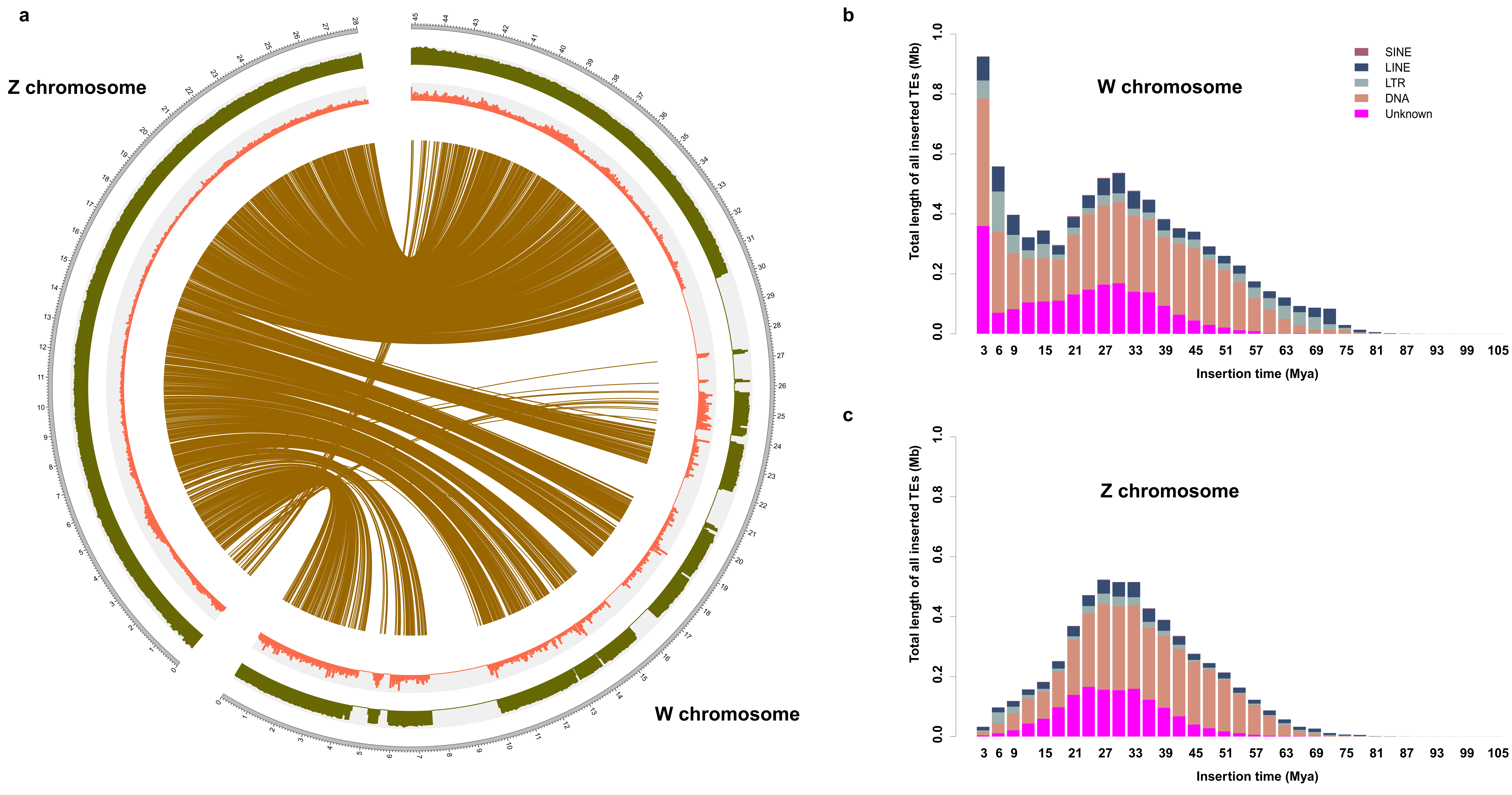

Click here to access/download
Supplementary Material
Supplemental_Information.docx

Click here to access/download
Supplementary Material
Additional_File.xlsx
