# Supplementary material for: Chromosome-level genome assembly of the female western mosquitofish (Gambusia affinis)
Source: Gigascience. 2020 Aug 27;9(8):giaa092. doi: 10.1093/gigascience/giaa092 (PMC7450667; doi:10.1093/gigascience/giaa092)
Supplement: giaa092_Supplemental_Files [file giaa092_supplemental_files.zip › Supplemental_Information.docx]

Supplementary Information for

Chromosome-level genome assembly of the female western mosquitofish (*Gambusia affinis*)

Feng Shao^1^, Arne Ludwig^2,3^, Yang Mao^1^, Ni Liu^1^, Zuogang Peng^1, *^

^1^ Key Laboratory of Freshwater Fish Reproduction and Development (Ministry of Education), Southwest University School of Life Sciences, Chongqing 400715, China

^2^ Department of Evolutionary Genetics, Leibniz-Institute for Zoo and Wildlife Research, 10315 Berlin, Germany

^3^ Albrecht Daniel Thaer-Institute, Faculty of Life Sciences, Humboldt University Berlin, 10115 Berlin, Germany

*Correspondence: pzg@swu.edu.cn

Table S1. Result of female and male *Gambusia affinis* genomic assembly at chromosome-level.

| Chr | Female (bp) | Male (bp) |
| --- | --- | --- |
| LG01 | 45,125,782 (W) | 28,011,121 (Z) |
| LG02 | 30,503,261 | 27,520,686 |
| LG03 | 31,977,068 | 28,940,732 |
| LG04 | 33,515,161 | 30,583,032 |
| LG05 | 32,673,047 | 28,852,092 |
| LG06 | 28,514,379 | 25,760,841 |
| LG07 | 29,761,488 | 25,694,439 |
| LG08 | 26,185,198 | 22,157,256 |
| LG09 | 29,879,072 | 27,775,116 |
| LG10 | 24,493,518 | 21,877,606 |
| LG11 | 26,918,255 | 24,845,306 |
| LG12 | 32,887,360 | 28,792,414 |
| LG13 | 27,482,798 | 23,023,579 |
| LG14 | 26,063,569 | 21,121,403 |
| LG15 | 23,540,282 | 21,557,523 |
| LG16 | 24,670,963 | 21,780,934 |
| LG17 | 19,955,793 | 17,818,601 |
| LG18 | 31,970,598 | 27,969,973 |
| LG19 | 26,195,410 | 24,091,954 |
| LG20 | 31,012,137 | 28,277,414 |
| LG21 | 23,709,503 | 21,272,223 |
| LG22 | 28,174,217 | 25,946,590 |
| LG23 | 30,252,746 | 27,552,808 |
| LG24 | 13,961,689 | 11,442,769 |
| Total | 679,423,294 | 592,666,412 |

Table S2. Transposable elements (TEs) annotation in the female *Gambusia affinis* genome.

| Class | Order | Super family | Number of | Length of | Percentage of |
| --- | --- | --- | --- | --- | --- |
|  |  |  | elements | sequence (bp) | sequence (%) |
| Class I |  |  | 174,996 | 35,050,740 | 5.15 |
|  | SINE |  | 23,922 | 3,455,238 | 0.51 |
|  |  | tRNA-L1 | 7,965 | 1,312,505 | 0.19 |
|  |  | tRNA-V | 9,615 | 1,291,083 | 0.19 |
|  |  | Other | 6,342 | 851,650 | 0.13 |
|  | LINE |  | 94,219 | 19,010,842 | 2.8 |
|  |  | L2 | 43,817 | 8,272,982 | 1.22 |
|  |  | Rex-Babar | 17,563 | 2,695,572 | 0.4 |
|  |  | L1 | 5,735 | 1,949,106 | 0.29 |
|  |  | RTE-BovB | 10,208 | 2,788,261 | 0.41 |
|  |  | Dong-R4 | 1,512 | 755,541 | 0.11 |
|  |  | Other | 15,384 | 2,549,380 | 0.37 |
|  | LTR |  | 56,855 | 12,584,660 | 1.85 |
|  |  | Gypsy | 21,839 | 5,482,617 | 0.81 |
|  |  | Ngaro | 15,519 | 2,875,294 | 0.42 |
|  |  | Pao | 2,930 | 1,300,316 | 0.19 |
|  |  | ERV1 | 7,531 | 844,716 | 0.12 |
|  |  | Unknown | 2,196 | 907,441 | 0.13 |
|  |  | Other | 6,840 | 1,174,276 | 0.17 |
| Class II |  |  | 547,959 | 80,444,161 | 11.83 |
|  | DNA |  | 531,209 | 76,737,912 | 11.28 |
|  |  | hAT-Charlie | 46,450 | 7,912,620 | 1.16 |
|  |  | TcMar-Tc1 | 203,534 | 30,549,842 | 4.49 |
|  |  | hAT-Tip100 | 14,626 | 1,958,810 | 0.29 |
|  |  | hAT-Ac | 89,384 | 15,516,258 | 2.28 |
|  |  | PIF-Harbinger | 31,819 | 5,942,177 | 0.87 |
|  |  | hAT-Blackjack | 9,081 | 1,413,604 | 0.21 |
|  |  | CMC-EnSpm | 28,512 | 3,169,994 | 0.47 |
|  |  | Unknown | 12,427 | 980,066 | 0.14 |
|  |  | hAT | 6,548 | 1,141,405 | 0.17 |
|  |  | hAT-hAT5 | 7,981 | 1,204,580 | 0.18 |
|  |  | Other | 80,847 | 6,948,556 | 1.02 |
|  | RC |  | 16,750 | 3,706,249 | 0.54 |
|  |  | Helitron | 16,750 | 3,706,249 | 0.54 |
| Unknown |  |  | 210,444 | 35,668,101 | 5.24 |
| Total TEs |  |  | 947,200 | 153,336,415 | 22.54 |

Table S3. Comparative analysis of the annotated gene set of female *Gambusia affinis* with those of five teleosts.

| Species | Total number of gene | Average transcript length(bp) | Average CDS length(bp) | Average exons number per gene | Average exon length(bp) | Average intron length(bp) |
| --- | --- | --- | --- | --- | --- | --- |
| *Danio rerio* | 25,122 | 26,634 | 1,597 | 9.31 | 171 | 3,011 |
| *G. affinis* | 23,997 | 13,586 | 1,623 | 9.56 | 169 | 1,397 |
| *Nothobranchius furzeri* | 26,141 | 17,117 | 1,516 | 8.66 | 175 | 2,037 |
| *Oryzias latipes* | 22,121 | 15,403 | 1,618 | 9.26 | 174 | 1,668 |
| *Poecilia formosa* | 23,615 | 14,815 | 1,663 | 10.4 | 159 | 1,398 |
| *Xiphophorus maculatus* | 23,628 | 15,599 | 1,629 | 9.41 | 173 | 1,660 |

Table S4. Assessment of female *Gambusia affinis* genome completeness by BUSCO.

| Type | Number | Percent (%) |
| --- | --- | --- |
| Complete BUSCOs (C) | 4,447 | 97.0 |
| Complete and single-copy BUSCOs (S) | 4,295 | 93.7 |
| Complete and duplicated BUSCOs (D) | 152 | 3.3 |
| Fragmented BUSCOs (F) | 93 | 2.0 |
| Missing BUSCOs (M) | 44 | 1.0 |
| Total BUSCO groups searched | 4,584 | 100 |

Table S5. Statistics for gene function annotation in female *Gambusia affinis* genome.

| Type | | Number | Percent (%) |
| --- | --- | --- | --- |
| Annotation | KOG | 15,858 | 66.08 |
|  | KEGG | 15,275 | 63.65 |
|  | NR | 23,559 | 98.17 |
|  | SwissProt | 22,282 | 92.85 |
|  | GO | 13,604 | 56.69 |
| Total | Annotated | 23,737 | 98.92 |
|  | Gene | 23,997 | - |

Table S6. Expansion gene families of female *Gambusia affinis* were enriched in 44 GO categories.

| ID | Description | GO_Class |
| --- | --- | --- |
| GO:0016758 | transferase activity, transferring hexosyl groups | MF |
| GO:0005044 | scavenger receptor activity | MF |
| GO:0004984 | olfactory receptor activity | MF |
| GO:0030286 | dynein complex | CC |
| GO:0000786 | nucleosome | CC |
| GO:0016705 | oxidoreductase activity, acting on paired donors, with incorporation or reduction of molecular oxygen | MF |
| GO:0007156 | homophilic cell adhesion via plasma membrane adhesion molecules | BP |
| GO:0003777 | microtubule motor activity | MF |
| GO:0051260 | protein homooligomerization | BP |
| GO:0020037 | heme binding | MF |
| GO:0003956 | NAD(P)+-protein-arginine ADP-ribosyltransferase activity | MF |
| GO:0007018 | microtubule-based movement | BP |
| GO:0006471 | protein ADP-ribosylation | BP |
| GO:0042981 | regulation of apoptotic process | BP |
| GO:0005506 | iron ion binding | MF |
| GO:0004675 | transmembrane receptor protein serine/threonine kinase activity | MF |
| GO:0004842 | ubiquitin-protein transferase activity | MF |
| GO:0008199 | ferric iron binding | MF |
| GO:0005741 | mitochondrial outer membrane | CC |
| GO:0016641 | oxidoreductase activity, acting on the CH-NH2 group of donors, oxygen as acceptor | MF |
| GO:0006879 | cellular iron ion homeostasis | BP |
| GO:0003887 | DNA-directed DNA polymerase activity | MF |
| GO:0003774 | motor activity | MF |
| GO:0016459 | myosin complex | CC |
| GO:0004602 | glutathione peroxidase activity | MF |
| GO:0006979 | response to oxidative stress | BP |
| GO:0005856 | cytoskeleton | CC |
| GO:0006334 | nucleosome assembly | BP |
| GO:0005201 | extracellular matrix structural constituent | MF |
| GO:0016887 | ATPase activity | MF |
| GO:0007219 | Notch signaling pathway | BP |
| GO:0015299 | solute:proton antiporter activity | MF |
| GO:0006811 | ion transport | BP |
| GO:0005507 | copper ion binding | MF |
| GO:0005216 | ion channel activity | MF |
| GO:0004252 | serine-type endopeptidase activity | MF |
| GO:0000166 | nucleotide binding | MF |
| GO:0004298 | threonine-type endopeptidase activity | MF |
| GO:0005839 | proteasome core complex | CC |
| GO:0051603 | proteolysis involved in cellular protein catabolic process | BP |
| GO:0042157 | lipoprotein metabolic process | BP |
| GO:0006260 | DNA replication | BP |
| GO:0006869 | lipid transport | BP |
| GO:0055114 | oxidation-reduction process | BP |

MF: molecular function, BP: biological process, CC: cellular component.

Table S7. Expansion gene families of female *Gambusia affinis* were enriched in 34 KEGG pathways.

| ID | Description |
| --- | --- |
| map00053 | Ascorbate and aldarate metabolism |
| map00140 | Steroid hormone biosynthesis |
| map04621 | NOD-like receptor signaling pathway |
| map00040 | Pentose and glucuronate interconversions |
| map04514 | Cell adhesion molecules (CAMs) |
| map00860 | Porphyrin and chlorophyll metabolism |
| map00980 | Metabolism of xenobiotics by cytochrome P450 |
| map00982 | Drug metabolism - cytochrome P450 |
| map04740 | Olfactory transduction |
| map00830 | Retinol metabolism |
| map04145 | Phagosome |
| map04650 | Natural killer cell mediated cytotoxicity |
| map00983 | Drug metabolism - other enzymes |
| map04974 | Protein digestion and absorption |
| map04210 | Apoptosis |
| map04612 | Antigen processing and presentation |
| map04360 | Axon guidance |
| map04750 | Inflammatory mediator regulation of TRP channels |
| map04138 | Autophagy - yeast |
| map02010 | ABC transporters |
| map00591 | Linoleic acid metabolism |
| map04978 | Mineral absorption |
| map04711 | Circadian rhythm - fly |
| map04392 | Hippo signaling pathway - multiple species |
| map04217 | Necroptosis |
| map04320 | Dorso-ventral axis formation |
| map00590 | Arachidonic acid metabolism |
| map04928 | Parathyroid hormone synthesis, secretion and action |
| map04975 | Fat digestion and absorption |
| map04960 | Aldosterone-regulated sodium reabsorption |
| map04215 | Apoptosis - multiple species |
| map04972 | Pancreatic secretion |
| map04216 | Ferroptosis |
| map04614 | Renin-angiotensin system |

Figure S1. Frequency distribution of the 17-mer graph analysis used to estimate the size of female *Gambusia affinis.*

Figure S2: Western mosquitofish genome scaffold contact matrix using Hi-C data. (a) Female western mosquitofish. (b) Male western mosquitofish. The color bar indicates the contact density from red (high) to white (low).


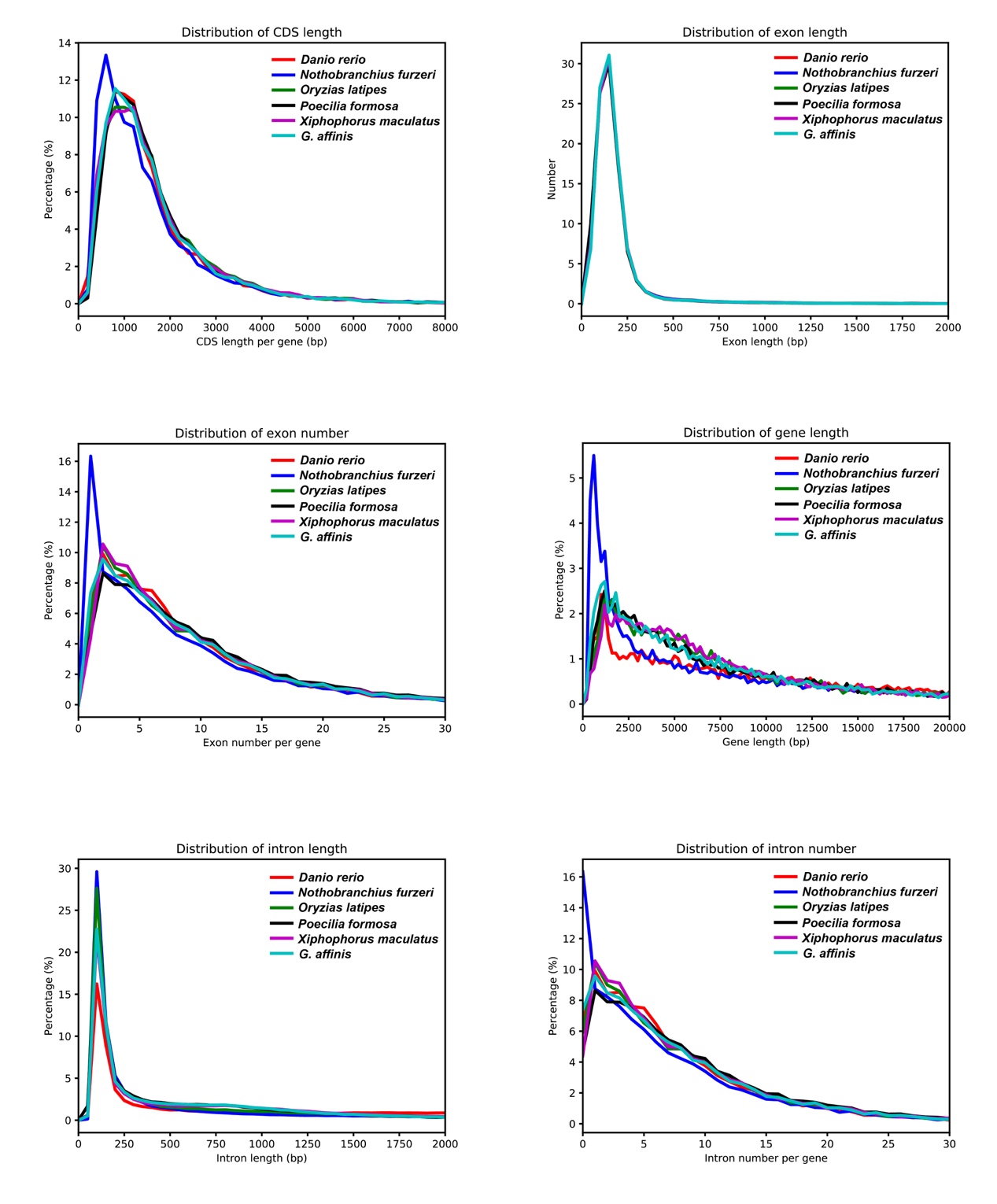


Figure S3. The comparisons of CDS length, exon length, exon-number, gene length, intro length, and intron number in genomes of female *Gambusia affinis* and other teleosts.

Figure S4. Divergence time of *Gambusia affinis* and other fish species.

Figure S5. Distribution of transposon activity time for different autosomes of female *Gambusia affinis*.
