# Supplementary material for: Chromosome-level genome assembly of the female western mosquitofish (Gambusia affinis)
Source: Gigascience. 2020 Aug 27;9(8):giaa092. doi: 10.1093/gigascience/giaa092 (PMC7450667; doi:10.1093/gigascience/giaa092)
Supplement: giaa092_Response_to_Reviewer_Comments_Original_Submission [file giaa092_response_to_reviewer_comments_original_submission.pdf]

Author's Response To Reviewer Comments

Close

Response to the Reviewers

Reviewer reports:
Reviewer #1: The authors of this manuscript performed a whole genome sequencing and assembly for Gambusia affinis, and report their product as the only chromosome-scale assembly for this species available for the moment. As an item to be published in the Data Note section of the journal, I propose the changes below.
I have a concern about the fact that the authors readily call their product sequences 'chromosomes'. I often see more qualified designation 'pseudochromosome' or 'Hi-C scaffold'.
Response: Thanks for your insightful comments. We had added a description in the MS as fellow "G. affinis genomic DNA sequences containing 217 contigs with an N50 length of 12.9 Mb and 125 scaffolds with an N50 length of 26.5 Mb was obtained by Oxford nanopore and BioNano, respectively, and the 113 scaffolds (90.4% of scaffolds containing 97.9% nucleotide bases) were assembled into 24 chromosomes (pseudo-chromosomes) by Hi-C." and "Accordingly, in this research, a chromosome (pseudo-chromosomes)-level genome assembly of female G. affinis was generated using Oxford nanopore, BioNano, and Hi-C technology." to reflect the chromosomes produced in this study were 'pseudo-chromosomes'.

I suggest the authors to more explicitly write 'DNA sequences', as what they produced is not a genome, but a set of genomic DNA sequences. This suggestion applies to other parts of the manuscript.
Response: Thanks. Again, very insightful comments. We had changed the word 'genome' to 'reference genome' or 'genomic DNA sequences' in the MS to reflect genome produced in this study was 'reference genome' or 'genomic DNA sequences'.

The detail of Hi-C library sequencing should be included in the Methods, including the length of reads. If there was any amendment process (manual curation usually called 'review') after running LACHESIS, the details of that step should also be included.
Response: Thanks for your insightful comments. We had added the data corresponding to the read length as "Finally, the Hi-C libraries were quantified and sequenced using an Illumina Hiseq platform (150-bp paired-end reads)." and "the clean paired-end reads (549 million paired-end reads; 81,713,239,521 bp) were then mapped to the draft assembled sequence" The detail amendment process description also had been added in MS as "The interaction heat map of the initial assembly results of LACHESIS was drawn, according to the interaction between different scaffolds, the position and direction of the scaffolds that obviously did not meet the chromosome interaction characteristics in the figure were adjusted. Of note, if there were situations in a scaffold itself that did not meet the chromosome interaction characteristics, the scaffold was interrupted. Next, the scaffolds were adjusted separately until the overall heat map conformed to the characteristics of chromosome interaction."

More information in Table 1 should substantiate the quality difference of the assemblies. What about including the number of gaps, number of ambiguous bases (N), and the percentage of bases contained in chromosome-sized sequences.
Response: Thanks. We had added the number of gaps and ambiguous bases data to the table. Since the assembly of male mosquitofish genome was based on published contig level results, most gaps in their results were generated in the process of Hi-C assisted assembly, so this was not comparable to gaps in female mosquitofish genome. According to female mosquitofish genome assembly result of Nanopore data, the length of its assembly is longer than that of male mosquitofish with Hi-C, so the assembly results of female mosquitofish is better than that of male mosquitofish genome in terms of completeness.
Nanopore BioNano Hi-C (♀) Hi-C (♂)
Total assembly size of contig/scaffold/chromosome (bp) 662,579,534 680,140,492 679,423,294* 592,666,412*
Number of contig/scaffold/chromosome 217 125 24 24
N50 contig/scaffold/chromosome length (bp) 12,906,370 26,455,434 29,761,488 25,946,590

N90 contig/scaffold/chromosome length (bp) 1,629,223 18,394,109 23,709,503 21,272,223
Longest contig/scaffold/chromosome (bp) 28,665,999 31,542,956 45,125,082 30,583,032
Number of gaps - 92 181 73657
Number of ambiguous bases (N) - 17560958 17570558 8312008

We use OrthoMCL -> We used OrthoMCL
Response: Thanks. We had revised it.

Reviewer #2: The authors have revised their manuscript based on the recommendation of reviewers and replied to the queries as well. Although the quality of the revised MS has improved substantially, compared to that of the original submission, several aspects of the revisions performed appear to be incomplete and new errors have been introduced during the corrections. All in all, the referee is on the opinion that further improvements would be needed, before the manuscript could be considered for publications (see below for details).

Comments on and/or criticisms of the corrections requested:

* In the previous round, this referee requested that gene sets of the two sex chromosomes (all genes residing on one or both sex chromosomes must be also listed in a new Supplementary Table). In their reply, the authors state that "we also submitted the results to GigaDB".
Here, this referee repeatedly requests, that a new Supplementary Excel Table showing all genes located on the two sex chromosomes with their location(s) indicated (i.e. Z, W or Z&W) must be added to the second revised version.
Response: Thanks for your comments. We had added these information in "Additional File".

* In the previous round, this referee was asking the following: "Was the individual used for sequencing a mature adult? How was the sex of the fish verified? Are there signs of obvious sexual dimorphism in Western mosquitofish?"
In their response, the authors confirmed that mature adult was used for sequencing, and western mosquitofish has obvious sexual dimorphism. This information must be added to the M&M section of the revised version.
Response: Thanks. We had added this information to Methods as fellow "sexual dimorphism is pronounced in G. affinis: the anal fin of adult females resembles the dorsal fins, while the anal fin of adult males is pointy and specialized for gonopodium."

* In the previous round, this referee has requested that various sets of data should be made available. In their replies, the authors typically stated that they submitted that data to GigaDB.
This referee requests that for all those datasets the exact location should be indicated in the text with a link.
Response: Thanks. We had added the reference in MS as fellow "Shao F, Ludwig A, Mao Y, Liu N and Peng Z. Supporting data for "Chromosome-level genome assembly of the female western mosquitofish (Gambusia affinis)". GigaScience Database. 2020. http://doi.org/10.5524/100778."

* In Results section of the original submission the authors claimed that "… the insertion time characteristics of the TEs on the W chromosome were specific because insertion time trends of autosomal TEs differed dramatically (Figure S4)". The referee disagrees with that statement, and requested detailed explanation and perhaps a revised discussion of this whole phenomenon in the revised version.
In their response, the authors stated that they have re-drawn this part in Figure 3 and Figure S5. The results show that the insertion time trends of autosomal and Z-chromosomal TEs are dramatically different from that of W chromosome.
This referee requests that the authors should provide a detailed explanation on how and why the procedure leading to the different insertion profiles was modified compared to that used in the earlier version.
Response: We are sorry for making the confusions. In fact, during the drawing process, we unified the length of the Y-axis (both changed to 1Mb) to make the difference easier to read. No other data has been changed. And, We had rephrased the sentence as "Indeed, our results indicated very recent mass insertion events of TEs into the W chromosome (Figure 3b), and the insertion time characteristics of the TEs into the W chromosome were specific because its insertion time trends were dramatically different from those of autosomal and Z-chromosomal TEs (Figure 3c and Figure S5)."

* The referee maintains that 'longer length' is not a proper English term. 'Increased length' should be

used.
Response: Thanks. We had revised it.

* Reference list: The formatting is still not entirely uniform.
Response: Thanks. We have carefully formatted the references.

Errors present in the original submission, but discovered by this referee only in this round:

* OE1: Conclusion, 3rd sentence: What exactly do the authors mean by 'preliminary study on the sex chromosome based on the specificity of the sex chromosome in female western mosquitofish'? This looks circular and vague…
Thanks. We had rephrased the sentence as "preliminary study on the W and Z sex chromosome differentiation based on the specificity of the sex chromosome in female western mosquitofish".

* Table 1: The source of data must be indicated for every column by inserting a reference or the term 'This study'.
Response: Thanks. We had added # and ## to distinguish the data source from our study or published data.

New problems introduced by the authors during the revision (pointers refer to the revised MsWord file with Tracked Changes):

* Page 2, Line 3: Please correct 'sexual dimorphism' to 'sexually dimorphic'.
Response: Thanks. We had revised it.

* P2, L7-9: Please eliminate the redundancy (i.e. female /G. affinis/).
Response: Thanks. We had rephrased the sentence as "However, there is a lack of high-quality genomic data for the female G. affinis; hence, this study aimed to generate a chromosome-level genome assembly for it."

* P4, L6-11: This sentence is way too long, and as such, it must be broken into 2-3 shorter ones.
Response: Thanks. We had rephrased the sentence as "Combined with comparative genomic analysis of the Z and W chromosomes, the high-quality genome produced in this study is expected to provide the foundation for research on the differentiation of sex chromosomes. These data may also help explore the molecular basis of the morphological differences between the G. affinis females and males and the characteristics of ovoviviparous reproduction and may aid further studies on functional genomics."

* P12, L11-15: Another long sentence that must be broken up.
Response: Thanks. We had rephrased the sentence as "A total of 30.5 Gb Illumina clean reads were used for analyzing female G. affinis genome size and heterozygosity using K-mer analysis. Based on 26,474,864,304 17-mers and a peak 17-mer depth of 37 (Figure S1), the estimated heterozygosity rate was approximately 0.42%, and the estimated genome size of female G. affinis was about 715 Mb. Of note, the estimated genome size is similar to that of the nuclear DNA content estimated in a previous study using flow cytometry (0.75 pg, approximately 733 Mb) [47]."

* P17, L4: What do the authors mean by 'chromosome in the recent past (later than 2-7 Mya)'? Shouldn't it be 'between 2-7 Mya'?
Response: Thanks. We had changed 'later than 2-7 Mya' to 'between 2-7 Mya'.

* P18, Availability of supporting data: A link to the exact location must be provided here as well.
Response: Thanks. We had added the reference in MS as fellow "Shao F, Ludwig A, Mao Y, Liu N and Peng Z. Supporting data for "Chromosome-level genome assembly of the female western mosquitofish (Gambusia affinis)". GigaScience Database. 2020. http://doi.org/10.5524/100778."

* Figure 3, legend: The last sentence is unnecessary and should be removed.
Response: Thanks. We had removed it.

* Supp. Figure S5, legend: The last sentence is unnecessary and imprecise and therefore should be removed.
Response: Thanks. We had removed it.

Close
