# Supplementary material for: Chromosome-level genome assembly of the female western mosquitofish (Gambusia affinis)
Source: Gigascience. 2020 Aug 27;9(8):giaa092. doi: 10.1093/gigascience/giaa092 (PMC7450667; doi:10.1093/gigascience/giaa092)
Supplement: giaa092_Response_to_Reviewer_Comments_Revision_1 [file giaa092_response_to_reviewer_comments_revision_1.pdf]

Author's Response To Reviewer Comments

Close

1) Please include ORCIDs for all authors on the title page, if available.
Response: Thanks. We had added it on the title page.

2) In line 186, instead of the URL (https://www.ncbi.nlm.nih.gov/assembly/GCA_003097735.1) please cite just the permanent NCBI accession number (URLs are not stable and may change over time)
Response: Thanks. We had changed it to 'GCA_003097735.1'.

3) line 200 ff, Danio rerio and other fish genomes: Please move citations of third-party data sources (e.g. (ftp://ftp.ensembl.org/pub/release-96/fasta/danio_rerio/dna/) to the bibliography, and cite them by number in the text. Please refer to our citation guidelines for the format.
https://academic.oup.com/gigascience/pages/instructions_to_authors#Preparing%20Main%20Manuscript%20Text
(example format to cite an FTP site: [xx] Doe, J: Trivial HTTP, RFC2169. ftp://ftp.isi.edu/in-notes/rfc2169.txt (1999). Accessed 12 Nov 1999.)
If available, please also mention BioProject IDs or other permanent (NCBI) accession numbers for these data sources.
Response: Thanks. We had rephrased the sentence as 'Danio rerio (GCA_000002035.4), Oryzias latipes (GCA_002234715.1), Nothobranchius furzeri (GCA_001465895.2), Xiphophorus maculatus (GCA_002775205.2), and Poecilia formosa (GCA_000485575.1) were used to predict G. affinis genes by GeMoMa.'

4) citation of software tools used: For all software tools you mention, rather than mentioning the URL in the main text, please cite the relevant published literature (or the project home page, if no publication is available that describes the tool) from the bibliography. Also if you mention other URLs in the main text, please move them to the bibliography and treat them as a citation.
Response: Thanks. We had changed all software tools URL to citations.

5) Please also add RRIDs (Research Resource Identifiers) for the software tools you used, if available. You can find RRIDs here: https://dknet.org/data/source/nlx_144509-1/search. For example the RRID for Augustus is (RRID:SCR_008417).
Response: Thanks. We had added RRIDs for the software tools we used.

6) Please also include the fishbase ID for G. affinis.
Response: Thanks. We had added 'Fishbase ID: 3215' in MS.

7) If you have one, please also include an image of a female (or maybe female and male, for comparison) G. affinis specimen as Figure 1.
Response: Thanks. We had added image of G. affinis as Figure 1.

Close
