# Supplementary material for: Chromosome-level genome assembly of the female western mosquitofish (Gambusia affinis)
Source: Gigascience. 2020 Aug 27;9(8):giaa092. doi: 10.1093/gigascience/giaa092 (PMC7450667; doi:10.1093/gigascience/giaa092)
Supplement: giaa092_Reviewer_2_Report_Original_Submission — LÃ¡szlÃ3 Orban, Ph.D. -- 7/13/2020 Reviewed [file giaa092_reviewer_2_report_original_submission.pdf]

Reviewer Report

Title: Chromosome-level genome assembly of the female western mosquitofish (Gambusia affinis)

Version: Original Submission **Date:** 7/13/2020

Reviewer name: LÃ¡szlÃ³ Orban, Ph.D.

Reviewer Comments to Author:

The authors have revised their manuscript based on the recommendation of reviewers and replied to the queries as well. Although the quality of the revised MS has improved substantially, compared to that of the original submission, several aspects of the revisions performed appear to be incomplete and new errors have been introduced during the corrections. All in all, the referee is on the opinion that further improvements would be needed, before the manuscript could be considered for publications (see below for details).

Comments on and/or criticisms of the corrections requested (:

* In the previous round, this referee requested that gene sets of the two sex chromosomes (all genes residing on one or both sex chromosomes must be also listed in a new Supplementary Table). In their reply, the authors state that "we also submitted the results to GigaDB".

Here, this referee repeatedly requests, that a new Supplementary Excel Table showing all genes located on the two sex chromosomes with their location(s) indicated (i.e. Z, W or Z&W) must be added to the second revised version.

* In the previous round, this referee was asking the following: "Was the individual used for sequencing a mature adult? How was the sex of the fish verified? Are there signs of obvious sexual dimorphism in Western mosquitofish?"

In their response, the authors confirmed that mature adult was used for sequencing, and western mosquitofish has obvious sexual dimorphism. This information must be added to the M&M section of the revised version.

* In the previous round, this referee has requested that various sets of data should be made available. In their replies, the authors typically stated that they submitted that data to GigaDB.

This referee requests that for all those datasets the exact location should be indicated in the text with a link.

* In Results section of the original submission the authors claimed that "… the insertion time characteristics of the TEs on the W chromosome were specific because insertion time trends of autosomal TEs differed dramatically (Figure S4)". The referee disagrees with that statement, and requested detailed explanation and perhaps a revised discussion of this whole phenomenon in the revised version.

In their response, the authors stated that they have re-drawn this part in Figure 3 and Figure S5. The results show that the insertion time trends of autosomal and Z-chromosomal TEs are dramatically different from that of W chromosome.

This referee requests that the authors should provide a detailed explanation on how and why the procedure leading to the different insertion profiles was modified compared to that used in the earlier

version.
* The referee maintains that 'longer length' is not a proper English term. 'Increased length' should be used.
* Reference list: The formatting is still not entirely uniform.
Errors present in the original submission, but discovered by this referee only in this round:
* OE1: Conclusion, 3rd sentence: What exactly do the authors mean by 'preliminary study on the sex chromosome based on the specificity of the sex chromosome in female western mosquitofish'? This looks circular and vague…
* Table 1: The source of data must be indicated for every column by inserting a reference or the term 'This study'.
New problems introduced by the authors during the revision (pointers refer to the revised MsWord file with Tracked Changes):
* Page 2, Line 3: Please correct 'sexual dimorphism' to 'sexually dimorphic'.
* P2, L7-9: Please eliminate the redundancy (i.e. female /G. affinis/).
* P4, L6-11: This sentence is way too long, and as such, it must be broken into 2-3 shorter ones.
* P12, L11-15: Another long sentence that must be broken up.
* P17, L4: What do the authors mean by 'chromosome in the recent past (later than 2-7 Mya)'? Shouldn't it be 'between 2-7 Mya'?
* P18, Availability of supporting data: A link to the exact location must be provided here as well.
* Figure 3, legend: The last sentence is unnecessary and should be removed.
* Supp. Figure S5, legend: The last sentence is unnecessary and imprecise and therefore should be removed

Level of Interest

Please indicate how interesting you found the manuscript: Choose an item.

Quality of Written English

Please indicate the quality of language in the manuscript: Choose an item.

Declaration of Competing Interests

Please complete a declaration of competing interests, considering the following questions:

- Have you in the past five years received reimbursements, fees, funding, or salary from an organisation that may in any way gain or lose financially from the publication of this manuscript, either now or in the future?
- Do you hold any stocks or shares in an organisation that may in any way gain or lose financially from the publication of this manuscript, either now or in the future?

- Do you hold or are you currently applying for any patents relating to the content of the manuscript?
- Have you received reimbursements, fees, funding, or salary from an organization that holds or has applied for patents relating to the content of the manuscript?
- Do you have any other financial competing interests?
- Do you have any non-financial competing interests in relation to this paper?

If you can answer no to all of the above, write 'I declare that I have no competing interests' below. If your reply is yes to any, please give details below.

No competing interests

I agree to the open peer review policy of the journal. I understand that my name will be included on my report to the authors and, if the manuscript is accepted for publication, my named report including any attachments I upload will be posted on the website along with the authors' responses. I agree for my report to be made available under an Open Access Creative Commons CC-BY license (http://creativecommons.org/licenses/by/4.0/). I understand that any comments which I do not wish to be included in my named report can be included as confidential comments to the editors, which will not be published.

Choose an item.

To further support our reviewers, we have joined with Publons, where you can gain additional credit to further highlight your hard work (see: https://publons.com/journal/530/gigascience). On publication of this paper, your review will be automatically added to Publons, you can then choose whether or not to claim your Publons credit. I understand this statement.

Yes Choose an item.
